# A Pliocene–Pleistocene continental biota from Venezuela

**DOI:** 10.1186/s13358-020-00216-6

**Published:** 2021-04-23

**Authors:** Jorge D. Carrillo-Briceño, Rodolfo Sánchez, Torsten M. Scheyer, Juan D. Carrillo, Massimo Delfino, Georgios L. Georgalis, Leonardo Kerber, Damián Ruiz-Ramoni, José L. O. Birindelli, Edwin-Alberto Cadena, Aldo F. Rincón, Martin Chavez-Hoffmeister, Alfredo A. Carlini, Mónica R. Carvalho, Raúl Trejos-Tamayo, Felipe Vallejo, Carlos Jaramillo, Douglas S. Jones, Marcelo R. Sánchez-Villagra

**Affiliations:** 1 Universität Zürich, Paläontologisches Institut und Museum, Karl-Schmid-Straße 4, 8006 Zurich, Switzerland; 2 Museo Paleontológico de Urumaco, Calle Bolívar s/n, Urumaco, Estado Falcón Venezuela; 3 CR2P, Muséum National d’Histoire Naturelle, CNRS, Sorbonne Université, 8 Rue Buffon, 75005 Paris, France; 4 Gothenburg Global Biodiversity Centre, Carl Skottsbergs gata 22B, 41319 Gothenburg, Sweden; 5 Dipartimento di Scienze della Terra, Università di Torino, Via Valperga Caluso 35, 10125 Torino, Italy; 6 Institut Català de Paleontologia Miquel Crusafont, Universitat Autònoma de Barcelona, Edifici ICTA/ICP, c/Columnes s/n, Campus de la UAB, 08193 Cerdanyola del Vallès, Barcelona Spain; 7 Centro de Apoio à Pesquisa Paleontológica da Quarta Colônia (CAPPA), Universidade Federal de Santa Maria (UFSM), São João do Polêsine, Rio Grande do Sul Brazil; 8 Museu Paraense Emílio Goeldi, Coordenação de Ciências da Terra e Ecologia, Belém, PA Brazil; 9 Centro Regional de Investigaciones Científicas y Transferencia Tecnológica de La Rioja (CRILAR), Provincia de La Rioja, CONICET, UNLaR, SEGEMAR, UNCa, Entre Ríos y Mendoza s/n, 5301 Anillaco, La Rioja, Argentina; 10 Departamento de Biologia Animal e Vegetal, Universidade Estadual de Londrina, Londrina, Brazil; 11 Grupo de Investigación Paleontología Neotropical Tradicional y Molecular (PaleoNeo), Facultad de Ciencias Naturales, Universidad del Rosario, Bogotá, Colombia; 12 Departamento de Física y Geociencias, Universidad del Norte, Km. 5 Vía Puerto Colombia, Barranquilla, Colombia; 13 Laboratorio de Paleontología, Instituto de Ciencias de La Tierra, Universidad Austral de Chile, Valdivia, Chile; 14 Lab. Morfología Evolutiva Desarrollo (MORPHOS), and División Paleontología de Vertebrados, Museo de La Plata, Facultad de Ciencias Naturales y Museo, Universidad Nacional de La Plata, Paseo del Bosque s/n, B1900FWA La Plata, Argentina; 15 Smithsonian Tropical Research Institute, Apartado, 0843-03092 Balboa, Ancón Panama; 16 Instituto de Investigaciones en Estratigrafía (IIES), Universidad de Caldas, Calle 65 #26-10, Manizales, Colombia; 17 Departamento de Geología, Universidad de Salamanca, 37008 Salamanca, Spain; 18 ISEM, U. Montpellier, CNRS, EPHE, IRD, Montpellier, France; 19 Florida Museum of Natural History, University of Florida, Gainesville, FL 32611 USA

**Keywords:** Neogene, Neotropics, Northern South America, Urumaco sequence, Paleodiversity, *Megaleporinus*, *Amblydoras*, *Anilius*, Camelidae, *Chapalmalania*

## Abstract

The Pliocene–Pleistocene transition in the Neotropics is poorly understood despite the major climatic changes that occurred at the onset of the Quaternary. The San Gregorio Formation, the younger unit of the Urumaco Sequence, preserves a fauna that documents this critical transition. We report stingrays, freshwater bony fishes, amphibians, crocodiles, lizards, snakes, aquatic and terrestrial turtles, and mammals. A total of 49 taxa are reported from the Vergel Member (late Pliocene) and nine taxa from the Cocuiza Member (Early Pleistocene), with 28 and 18 taxa reported for the first time in the Urumaco sequence and Venezuela, respectively. Our findings include the first fossil record of the freshwater fishes *Megaleporinus*, *Schizodon*, *Amblydoras*, *Scorpiodoras*, and the pipesnake *Anilius scytale*, all from Pliocene strata. The late Pliocene and Early Pleistocene ages proposed here for the Vergel and Cocuiza members, respectively, are supported by their stratigraphic position, palynology, nannoplankton, and ^86^Sr/^88^Sr dating. Mammals from the Vergel Member are associated with the first major pulse of the Great American Biotic Interchange. In contrast to the dry conditions prevailing today, the San Gregorio Formation documents mixed open grassland/forest areas surrounding permanent freshwater systems, following the isolation of the northern South American basin from western Amazonia. These findings support the hypothesis that range contraction of many taxa to their current distribution in northern South America occurred rapidly during at least the last 1.5 million years.

## Introduction

During the Miocene, the coastal marine areas of northern South America arid regions of northern Colombia and northwestern Venezuela today were influenced by a complex hydrographic system that flowed mostly from western Amazonia into the Proto-Caribbean Sea (Díaz de Gamero 1996; Hoorn et al. 2010; Aguilera et al. 2013). Some of the best-known terrestrial and aquatic vertebrate faunas that document the changing biodiversity during that time are preserved in the middle–late Miocene Socorro and Urumaco formations, in northwestern Venezuela (e.g., Sánchez-Villagra et al. 2010). Diverse assemblages of aquatic and terrestrial vertebrates, such as fishes, amphibians, turtles, crocodylians, snakes, and mammals, have been reported from these sedimentary units that accumulated in a coastal plain-delta system (Lundberg and Aguilera 2003; Aguilera 2004; Linares 2004; Aguilera et al. 2006; Hsiou and Albino 2010; Lundberg et al. 2010; Quiroz and Jaramillo 2010; Sánchez-Villagra et al. 2010; Aguilera et al. 2013; Scheyer et al. 2013, 2019; Forasiepi et al. 2014; Rincón et al. 2015; Scheyer and Delfino 2016; Aguirre-Fernández et al. 2017a, b; Carrillo-Briceño et al. 2018b; Delfino and Sánchez-Villagra 2018; Cadena et al. 2020; and references therein).

The hydrographic connections between western Amazonia and the Proto-Caribbean Sea were home for many freshwater species, with a continuum of the biota from the Amazonian forest into northwestern Venezuela during the Miocene (Jaramillo et al. 2010). By the late Miocene to early Pliocene, extreme environmental changes took place in the region (Jaramillo et al. 2010; Sánchez-Villagra et al. 2010; Scheyer et al. 2013). These changes were linked to major hydrographic processes that occurred as a consequence of the northern Andes uplift (Mora et al. 2010; Albert et al. 2018), which may have led to the separation between the northern peripheral drainages of western Amazonia, creating habitat partitioning that drove vicariance in many groups including fishes, crocodylians, turtles, and aquatic snake communities (Lundberg et al. 1998, 2010; Schargel et al. 2007; Sánchez-Villagra et al. 2010; Scheyer et al. 2013; Cadena et al. 2020). The transition between the Urumaco Formation and the overlying latest Miocene–early Pliocene Codore Formation (Quiroz and Jaramillo 2010) documents a major turn in the dynamics of the sedimentary and environmental conditions of the region. The continental facies of the Codore Formation (El Jebe and Algodones members) were deposited in floodplain environments (exposed during long periods to subaerial conditions) (Quiroz and Jaramillo 2010).

Although the environmental and faunal changes that occurred during the Miocene–Pliocene transition in the Urumaco region and adjacent areas are unmistakable (e.g., Jaramillo et al. 2010; Lundberg et al. 2010; Scheyer et al. 2013), the record of Pliocene–Pleistocene terrestrial and freshwater vertebrates in the Urumaco sequence still consists of isolated reports, such as from the Codore and San Gregorio formations, that resulted from occasional prospecting in the area. Some terrestrial vertebrates have been described from the continental facies of the Codore Formation (El Jebe and Algodones members), including a Jabiru stork (Walsh and Sánchez 2008), sloths (Carlini et al. 2006a, b), glyptodontids (Carlini et al. 2008a, b), and meridiungulates (Carrillo et al. 2018). A skull and an isolated ear bone of iniid dolphins (Aguirre-Fernández et al. 2017a, b) were recovered from the Chiguaje Member of the Codore Formation, representing a transgressive event associated with a low-energy coastal lagoon or bay (Quiroz and Jaramillo 2010; Carrillo-Briceño et al. 2015).

The Vergel Member is the lowest member of the San Gregorio Formation and overlies the Algodones Member of the Codore Formation (Quiroz and Jaramillo 2010). The fossil record of this Member includes thorny catfish (Aguilera et al. 2013), crocodylians (Scheyer et al. 2013), terrestrial sloths, glyptodontids, pampatheriids and dasypodid armadillos (Carlini et al. 2008c, 2018; Carlini and Zurita 2010; Vucetich et al. 2010; Zurita et al. 2011; Castro et al. 2014), notoungulates (Carrillo et al. 2018), a procyonid (Forasiepi et al. 2014), and caviomorph rodents (Vucetich et al. 2010).

Based on thorny catfish remains from the Vergel Member as well as other freshwater Siluriformes from the Pliocene of the Cocinetas Basin in the Guajira Peninsula (northern Colombia), Aguilera et al. (2013) suggested the existence of a probable “last hydrographic connection” between northern South American and western Amazonian basins between 3.2 and 1.7 Ma. Diverse reports (e.g., Carlini and Zurita 2010; Vucetich et al. 2010; Aguilera et al. 2013; Scheyer et al. 2013; Castro et al. 2014; Forasiepi et al. 2014; Carlini et al. 2018; Carrillo et al. 2018) demonstrated the potential of the San Gregorio Formation to preserve evidence of the biota at the otherwise poorly sampled Pliocene–Pleistocene strata in this region of South America. Thus, our ongoing efforts continued throughout the 2007–2020 period in the search for microvertebrates and other fossils we report in this work.

Here, we present new terrestrial and aquatic vertebrates from the late Pliocene–Pleistocene San Gregorio Formation. Two different assemblages are described from the Vergel (lower) and Cocuiza (middle) members. A new paleoenvironmental interpretation, as well as new dating for the San Gregorio Formation, is proposed. Based on the taxonomic affinities of this fauna with those from the Orinoco/Amazonian systems, and Caribbean basins of Colombia and Venezuela, we discuss its paleogeographic and biochronologic significance. Our results shed light on the evolutionary history of the terrestrial and freshwater vertebrate faunas of the region, particularly during the transitional stage that preceded the major climatic shift of the Quaternary.

## Geological and stratigraphical settings

**Fig. 1:**
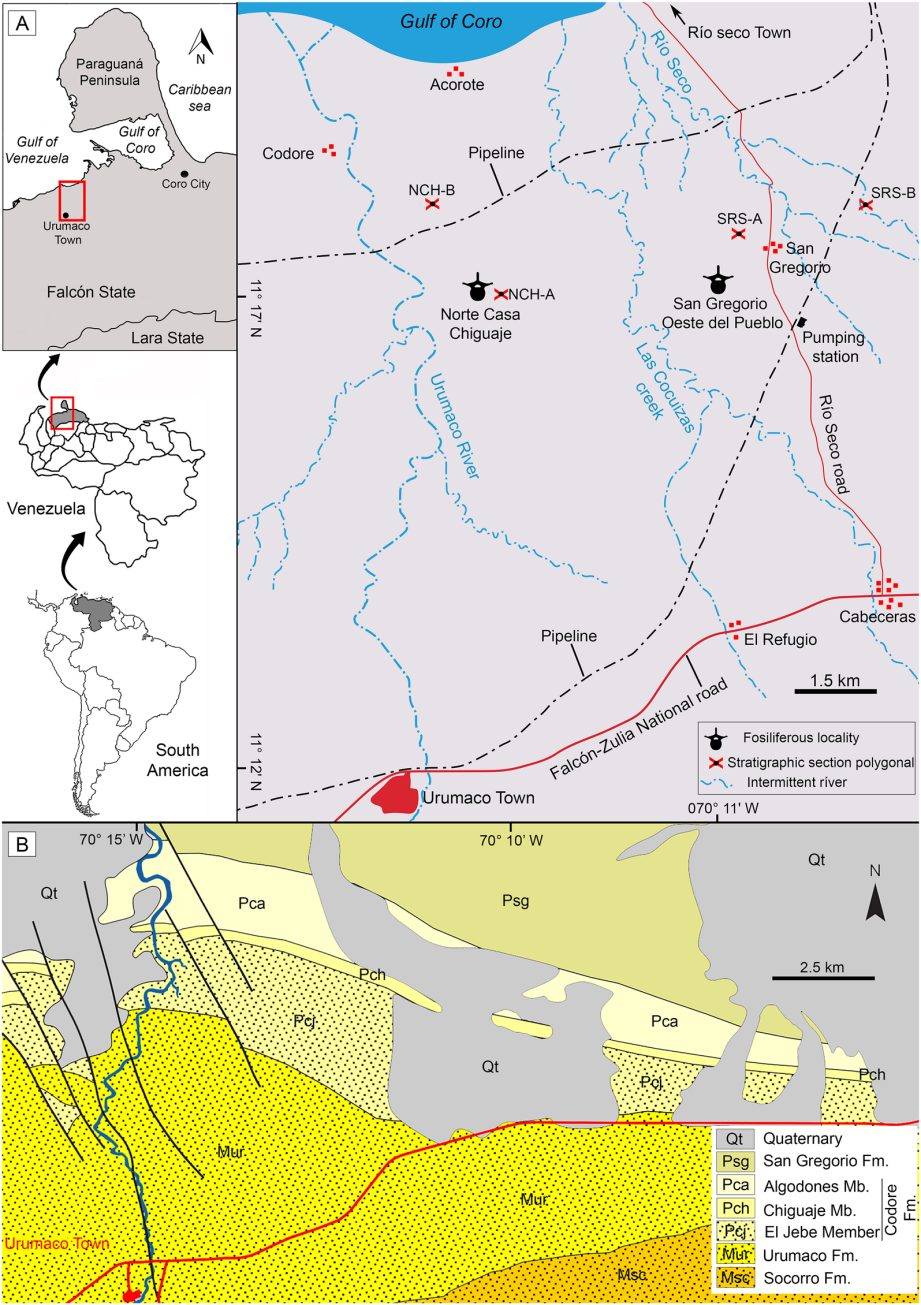
Geographical and geological location. **A** Location map of the fossiliferous localities of the San Gregorio Formation. Stratigraphic section polygonal: Northward Chiguaje Hill “NCH” (see Additional file 1) and San Gregorio Río Seco “SGRS” (see Additional file 2). **B** Generalized geological map near the town of Urumaco showing the outcropping geological units in the area (modified after Quiroz and Jaramillo 2010). *Fm.* Formation, *Mb.* Member

The San Gregorio Formation is the youngest unit of the Urumaco stratigraphic section (see Quiroz and Jaramillo 2010). This unit overlies unconformably the Algodones Member of the Codore Formation (Fig. 1B) and crops out northwest of the town of Urumaco, Falcón State, in northwestern Venezuela (Fig. 1A). The San Gregorio Formation is 570 m thick at its type section (see Stainforth 1962; Rey 1990; Hambalek et al. 1994), which is located 2 km east of the village of San Gregorio and one km east of the Ulé-Amuay pipeline. The age of the San Gregorio Formation has been considered either Pliocene or Early Pleistocene based on its stratigraphic position and palynological data (e.g., Rey 1990; Hambalek et al. 1994). Recently, an age of approximately 1.8 Ma was suggested for the boundary between the top of the Algodones Member and the base of the San Gregorio Formation and supported by multiple foraminiferal, nannoplankton, and magnetic stratigraphic studies (Carrillo et al. 2018, table 12, fig. 28).

Stainforth (1962) suggested the division of the San Gregorio Formation into three formal members: Vergel (lower), Cocuiza (middle), and Río Seco (upper). The Vergel Member is a ~ 260 m thick sequence (Hambalek et al. 1994) composed of interbedding mudstones, sandstones, and sparse conglomeratic beds (Stainforth 1962) that denote fluvial environments (e.g., braided rivers) and alluvial fans (Rey 1990; Hambalek et al. 1994; Quiroz and Jaramillo 2010). Overlying conformably the Vergel Member is the Cocuiza Member, an 80-m thick sequence (in its type section), dominated by marine deposits with low to moderate energy coastline, and local presence of terrigenous sediments (Rey 1990; Hambalek et al. 1994; Mihaljević et al. 2010). The Río Seco Member (142 m thick in its type section) overlies the Cocuiza Member and is characterized by interbedding of mudstones, sandstones, and conglomerates representing a sedimentary accumulation in fluvial environments and alluvial fans (Rey 1990).

**Fig. 2:**
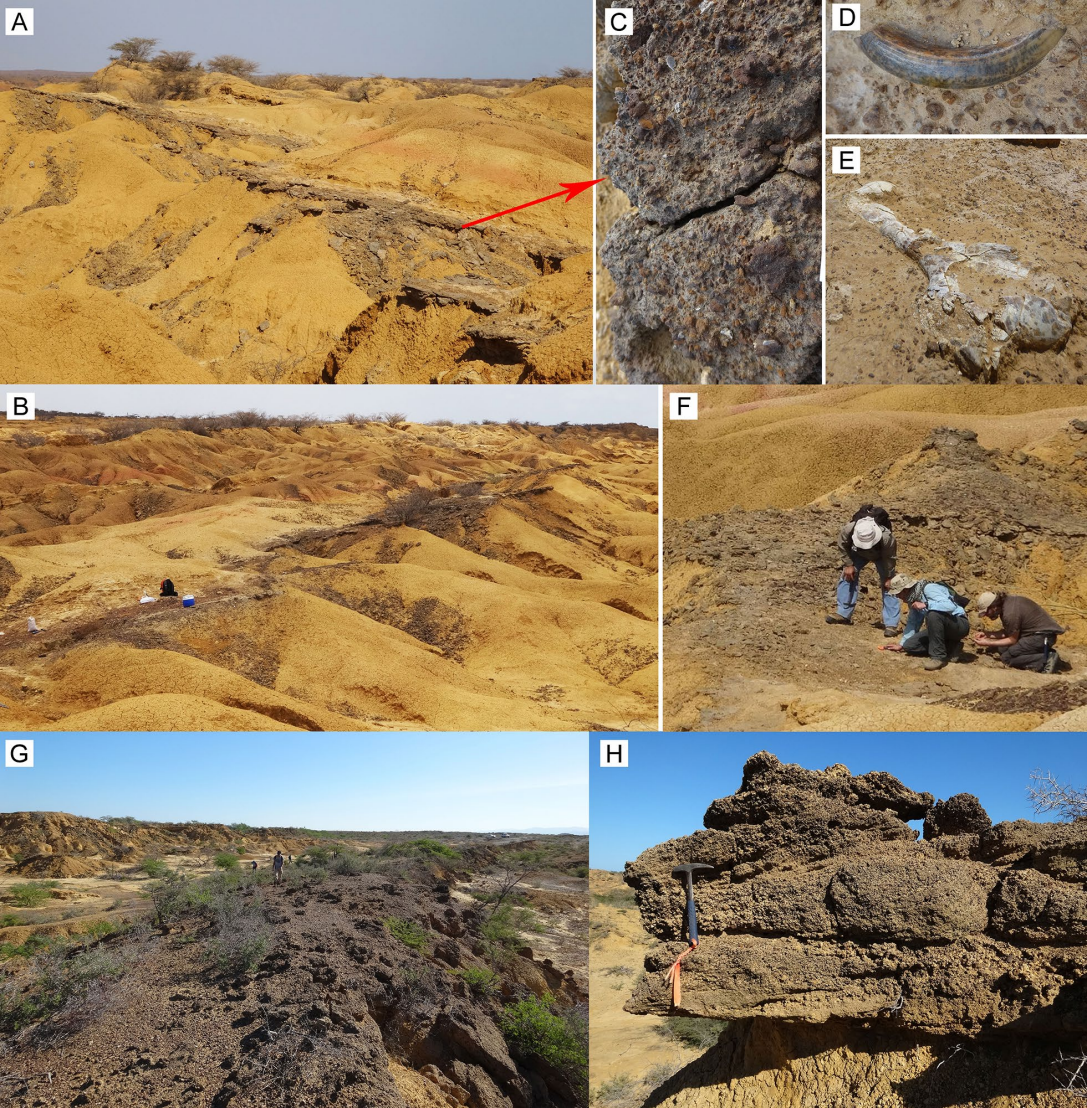
Fossiliferous localities of the San Gregorio Formation outcrops. **A**–**F** Norte Casa Chiguaje locality. **C** Close-up of the conglomeratic-fossiliferous layer. **D**, **E** Tooth (**D**) and humerus (**E** AMU-CURS-62) of mammals in situ. **G**, **H** San Gregorio Oeste del Pueblo locality. **H** Example of the conglomeratic layers were disarticulated continental vertebrates remains were found. Pictures from authors JDCB (**A**–**D**, **G**, **H**) and EAC (**F**)

**Fig. 3:**
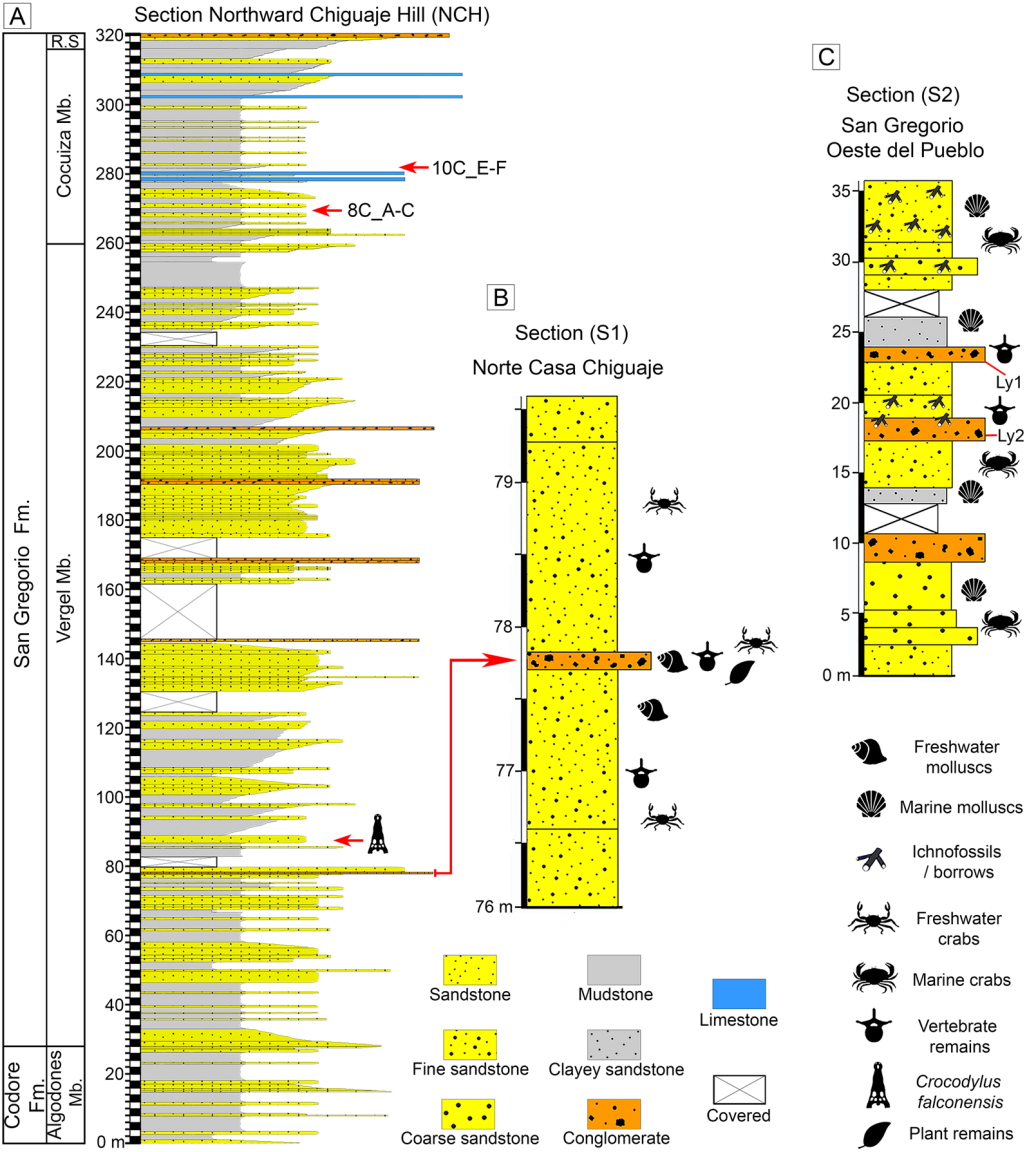
Stratigraphic context of the San Gregorio Formation. **A** Generalized section of Northward Chiguaje Hill polygonal (NCH) (see Fig. 1); a section with more detail of the NCH is presented in Additional file 1. **B** Norte Casa Chiguaje locality section. **C** San Gregorio Oeste del Pueblo locality section. The “8C_A-C” and “10C_E-F” referred in the NCH section correspond molluscan shell samples used for ^87^Sr/^86^Sr analyses (see Additional file 5). *Fm.* Formation, *Mb.* Member, *R.S* Río Seco Member

The fossiliferous content of the “Norte Casa Chiguaje” locality (NCC) in the Vergel Member (Figs. 1A, 2A–F and 3A, B) and the “San Gregorio Oeste del Pueblo” locality (SGOP) in the Cocuiza Member (Figs. 1A, 2G, H and 3C) are described. Both localities crop out in badlands, offering excellent exposures that allowed the identification of fossil-bearing layers in the field. The NCC locality and its section “S1” (Fig. 3B) is part of the “Section Northward Chiguaje Hill” (NCH) (Figs. 1A and 3A), which is presented in more detail in Additional file 1. The “S1” section is characterized by unconsolidated yellowish-orange to light-brown fine sandstones with a thin (~ 30 cm) consolidated conglomeratic layer of a light–dark gray matrix with well-rounded to sub-rounded clasts of up to 25 mm in diameter (Fig. 2A–C). This conglomeratic layer is well exposed along 180 m with a direction of N 55° W (Fig. 2A, B). Some cranial and postcranial elements were collected from the sandstones underlying/overlying the conglomerate layer. Most of the fossils collected in the conglomeratic layer were small/micro elements, and in many cases were fractured and incomplete, suggesting a taphonomic effect of transport. The mammalian remains described in Carlini et al. (2008c, 2018), Vucetich et al. (2010), Zurita et al. (2011), Castro et al. (2014), Forasiepi et al. (2014), and Carrillo et al. (2018) were collected in this locality. A late Pliocene age has been estimated for the Vergel Member based on its stratigraphic position and mammalian association (Carlini and Zurita 2010; Zurita et al. 2011; Castro et al. 2014).

The SGOP locality section “S2” (Figs. 1A and 3C) is a fossiliferous locality discovered in 2019. A detailed stratigraphic section for locality “S2” is presented (Fig. 3C); however, we have not been able to correlate it to either the NCH (Figs. 1A and 3A; Additional file 1) or the San Gregorio Río Seco (SGRS) (Fig. 1; Additional file 2) sections. Field observations and its marine fossil content suggest that the SGOP locality might belong to the middle section of the Cocuiza Member. The SGOP locality is characterized by an interbedding of clay-rich to coarse sandstones with abundant marine invertebrates, including sirenian remains, and some conglomeratic sandstones layers (Fig. 2G, H). The conglomerates are consolidated and dark-brown in color with well-rounded to sub-rounded clasts, bearing disarticulated terrestrial vertebrates (mammals and reptiles), suggesting intermittent flows of terrigenous sediments to the littoral environment. A Pliocene age was estimated for the Cocuiza Member based on palynomorphs (Stainforth 1962; Hambalek et al. 1994).

## Materials and methods

**Fig. 4:**
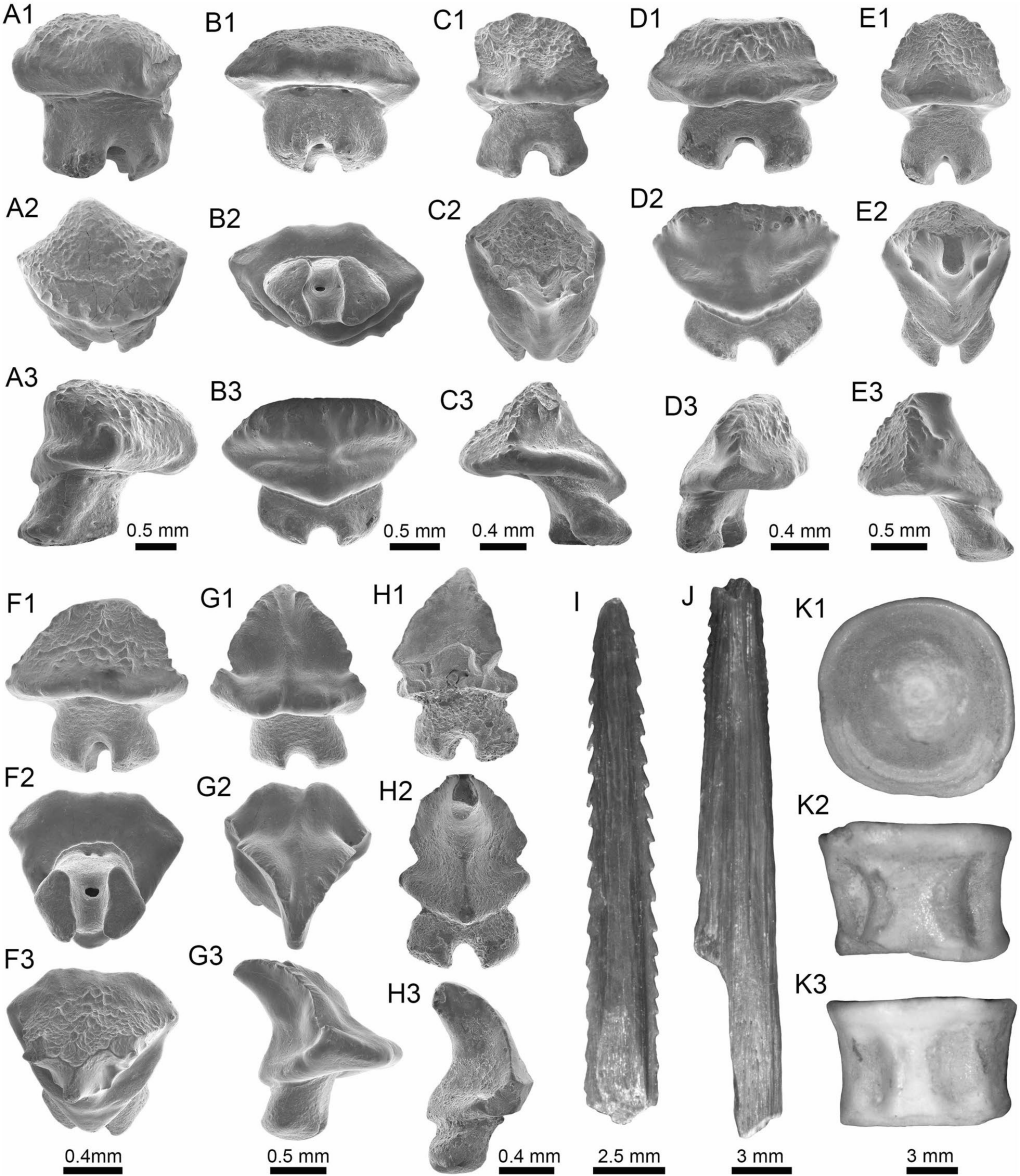
Stingrays from the Vergel Member. **A1**–**J** *Potamotrygon* sp. (teeth **A1**–**B3** AMU-CURS-868; **C1**–**D3** AMU-CURS-869; **E1**–**F3** AMU-CURS-870; **G1**–**H3** AMU-CURS-871; and caudal spines **I**, **J** AMU-CURS-865). **K1**–**K3** Myliobatiformes indet. Views: labial (**A1**, **B1**, **C1**, **D1**, **E1**, **F1**, **G1**, **H1**), lingual (**B3**, **D2**, **H2**), occlusal (**A2**, **C2**, **E2**, **F3**, **G2**), lateral (**A3**, **C3**, **D3**, **E3**, **G3**, **H3**), basal (**B2**, **F2**), ventral (**I**, **J**), and indet. (**K1**–**K3**)

**Fig. 5:**
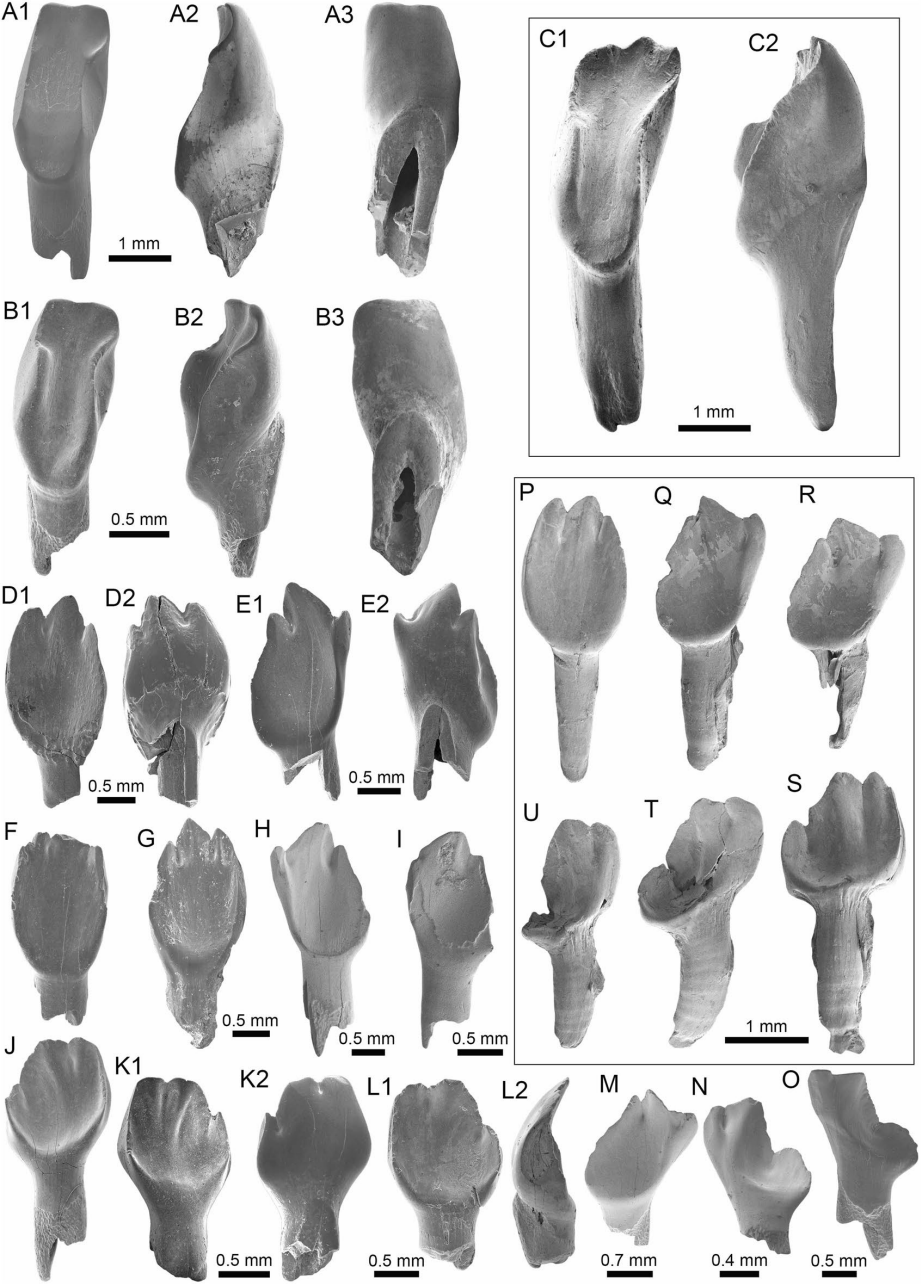
Characiformes (Anostomidae) from the Vergel Member. **A1**–**B3** Premaxillary symphyseal teeth of cf. *Megaleporinus* sp. (AMU-CURS-851). **C1**, **C2** Recent premaxillary symphyseal tooth of *Megaleporinus muyscorum* (PIMUZ A/I 4856). **D1**–**O** *Schizodon* cf. *S*. *corti* teeth (**D1**–**G**, **J**–**L** AMU-CURS-849, and **H**, **I**, **M**–**O** AMU-CURS-849), of both premaxillary (**D1**–**I**) and dentary position (**J**–**O**). **P**–**U** Recent teeth of *Schizodon corti* (PIMUZ A/I 4869) [premaxillary (**P**–**R**) and dentary teeth (**S**–**U**), symphyseal teeth (**P** and **S**)]. Views: labial (**A3**, **B3**, **D2**, **E2**, **K2**), lingual (**A1**, **B1**, **C1**, **D1**, **E1**, **F-K1**, **L1**, **M-U**), and lateral (**A2**, **B2**, **C2**, **L2**)

**Fig. 6:**
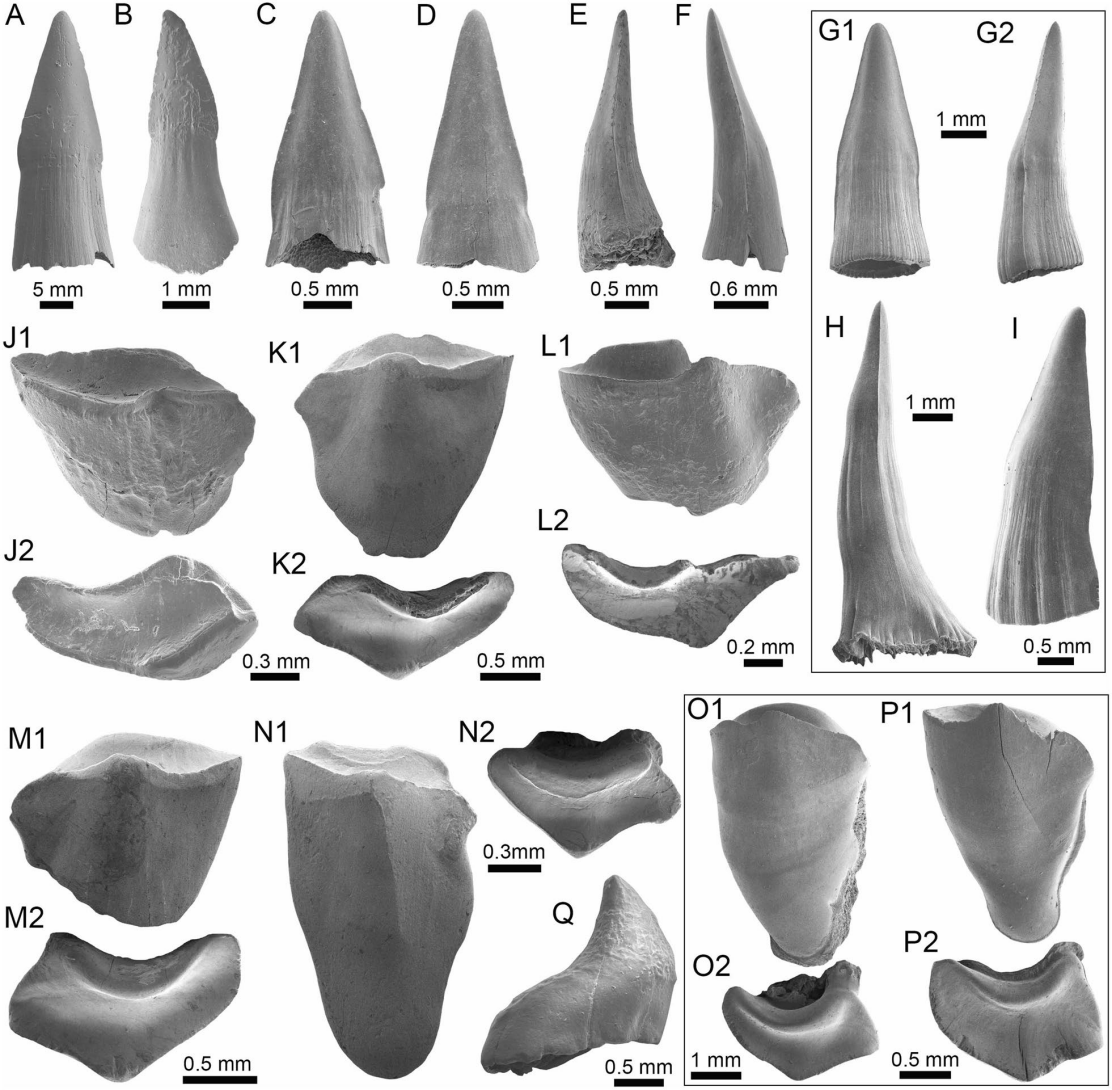
Characiformes (Erythrinidae and Serrasalmidae) from the Vergel Member. **A**–**F** *Hoplias* sp. teeth of indeterminate position (**A**, **B** AMU-CURS-860 and **C**–**F** AMU-CURS-824). **G1**–**I** Recent teeth of *Hoplias malabaricus* (PIMUZ A/I 4875). **J1**–**N2** *Mylossoma* sp. symphyseal dentary teeth (AMU-CURS-1216). **O1**–**P2** Recent symphyseal dentary teeth of *Mylossoma albiscopum* (PIMUZ A/I 4860). **Q** Serrasalmidae indet. (? “pacu clade”) dentary tooth (AMU-CURS-859). Views: labial (**A**, **D**, **I**, **J1**, **K1**, **L1**, **M1**, **N1**, **O1**, **P1**), lingual (**B**, **C**, **G1**), occlusal (**J2**, **K2**, **L2**, **M2**, **N2**, **O2**, **P2**), and lateral (**E**, **F**, **G2**, **H**, **Q**)

**Fig. 7:**
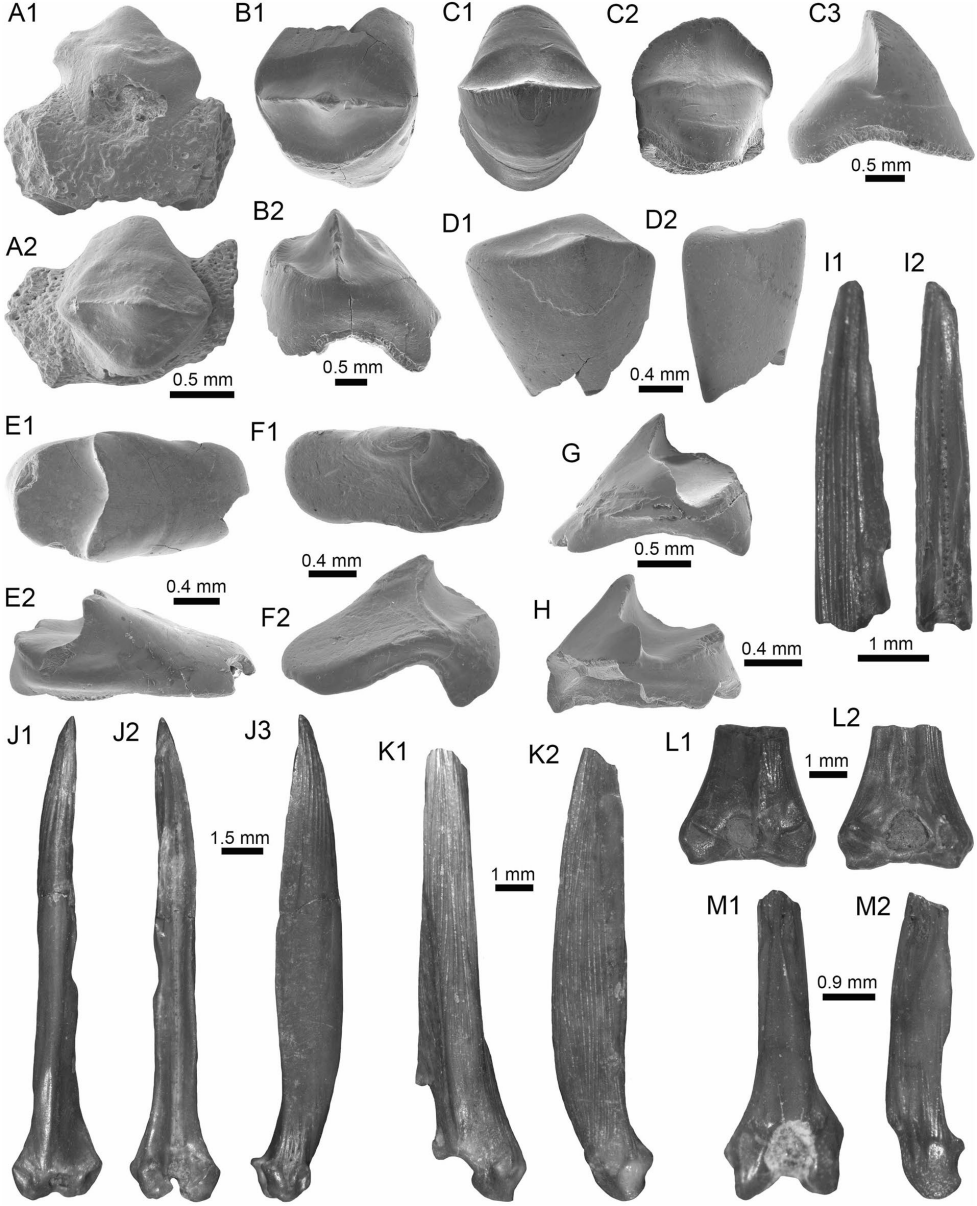
Characiformes (Serrasalmidae) and Cichliformes from the Vergel Member. **A1**–**H** Serrasalmidae indet. (? “pacu clade”). **A1**–**D2** Molariform-like teeth from the outer row and **E1**–**H** from the inner row of indeterminate (dentary/premaxilla) position (**A1**, **A2** AMU-CURS-859 and **B1**–**H** AMU-CURS-858). **I1**–**M2** Dorsal (**J1**–**J3**, **M1**, **M2**), anal (**K1**, **K2**) and indeterminate position (**I1**, **I2**, **L1**, **L2**) fin spines of Cichlidae indet. (**I1**, **I2**, **L1**–**M2** AMU-CURS-1224 and **J1**–**K2** AMU-CURS-1223). Views: labial (**A1**, **D1**), lingual (**C2**), occlusal (**A2**, **B1**, **C1**, **E1**, **F1**), lateral (**B2**, **C3**, **D2**, **E2**, **F2**, **G**–**I1**, **J3**, **K2**, **M2**), anterior (**J1**, **K1**, **L1**, **M1**), and posterior (**I2**, **J2**, **L2**)

**Fig. 8:**
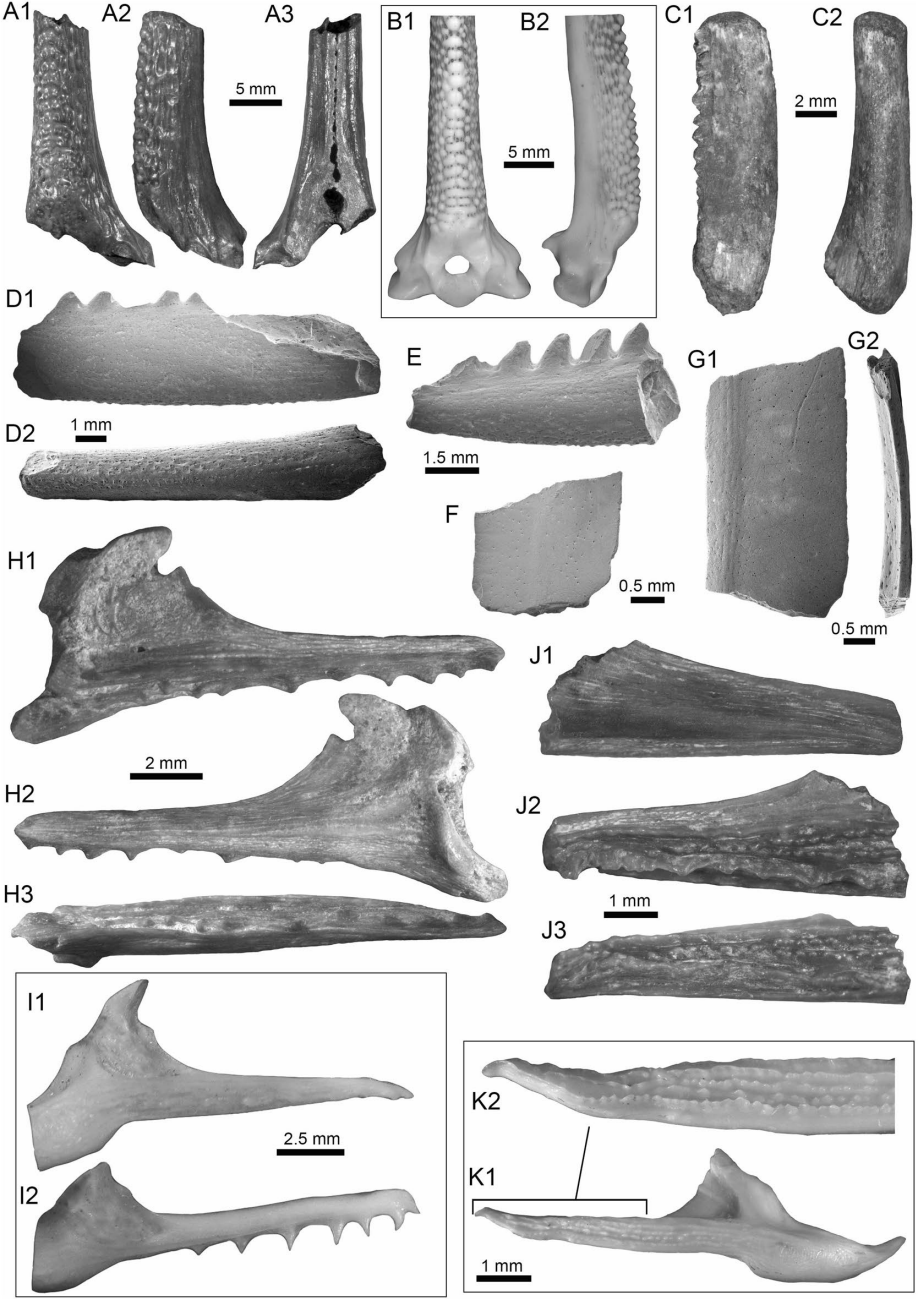
Siluriformes (Ariidae, Callichthyidae, and Doradidae) from the Vergel Member. **A1**–**A3** Dorsal spine of cf. *Sciades* sp. (AMU-CURS-1058). **B1**, **B2** Recent dorsal spine of *Sciades proops* (PIMUZ A/I 4874). **C1**–**E** Pectoral-fin spines and **F**–**G2** bony plate fragments of Callichthyidae indet. (**C1**, **C2** left spine, AMU-CURS-1167a; **D1**–**E** AMU-CURS-1168 and **F**–**G2** AMU-CURS-1169). **H1**–**H3** Partial left cleithrum cf. *Amblydoras* sp. (AMU-CURS-1225). **I1**, **I2** Recent left cleithrum of *Amblydoras affinis* (CAS 66236). **J1**–**J3** Partial right cleithrum of cf. *Scorpiodoras* sp. (AMU-CURS-1226). **K1**, **K2** Recent left cleithrum of *Scorpiodoras heckelii* (MHNLS-17959). Views: anterior (**A1**, **B1**, **C2**, **D2**), dorsal (**C1**, **I2**), dorsolateral (**H1**, **J2**), lateral (**A2**, **B2**, **F**–**G1**, **H3**, **J3**, **K1**, **K2**), mesial (**J1**), posterior (**A3**), ventral (**H2**), and indet. (**D1**, **E**, **G2**)

**Fig. 9:**
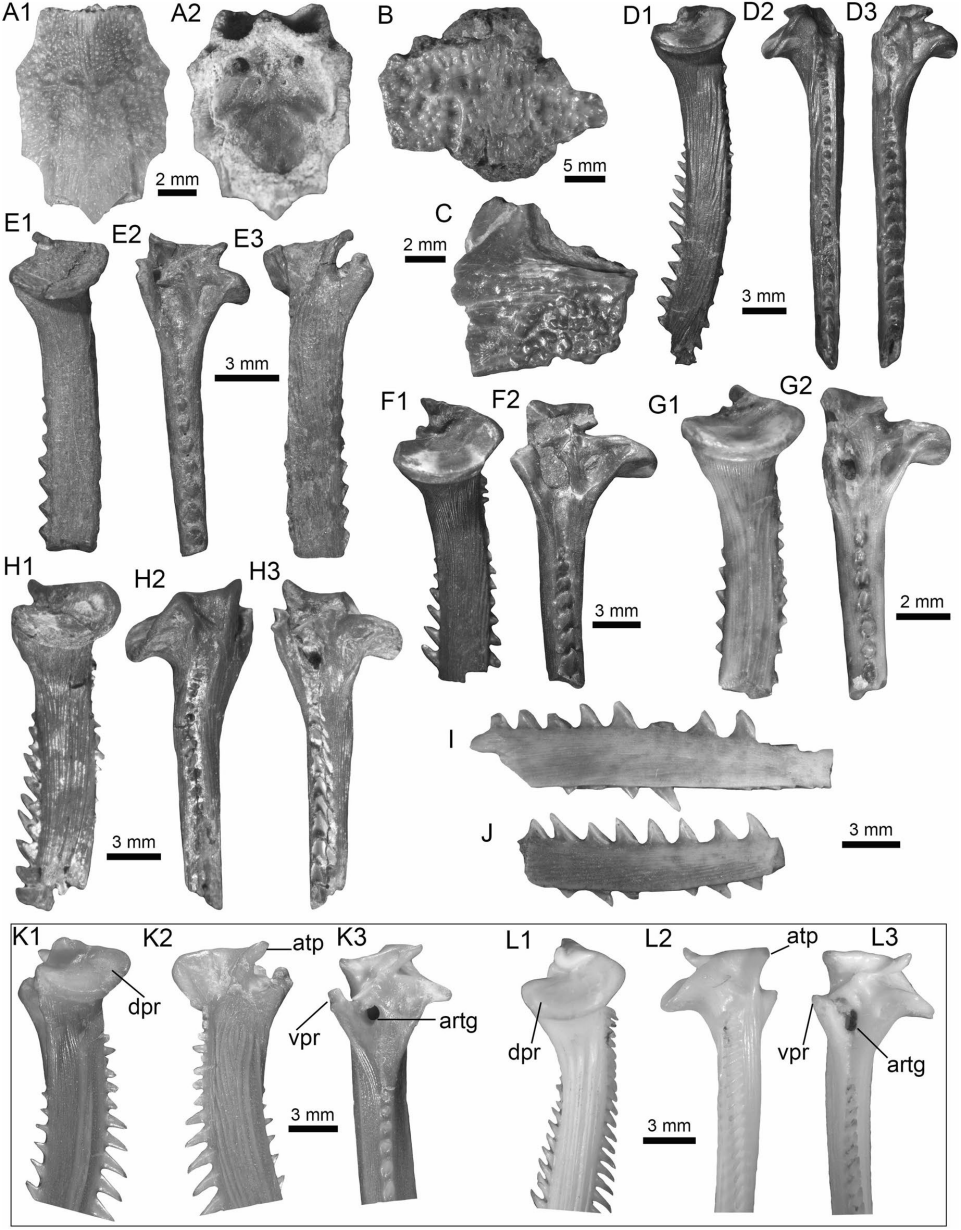
Siluriformes (Doradidae) from the Vergel Member. **A1**–**J** Doradidae indet. **A1**, **A2** Parieto-supraoccipital (AMU-CURS-1227). **B** Partial parieto-supraoccipital (AMU-CURS-1170). **C** Partial left cleithrum (AMU-CURS-1175). **D1**–**J** Pectoral-fin spines (**D1**–**H3** AMU-CURS-1234 and **I**, **J** AMU-CURS-1233), position: right (**D1**–**H3**) and indet. (**I**, **J**). **K1**–**L3** Right pectoral-fin spines of extant *Anadoras wedellii* (**K1**–**K3** AUM-45441) and *Scorpiodoras heckelii* (**L1**–**L3** MHNLS-17959). Views: anterior (**D2**, **H2**, **L2**), dorsal (**A1**, **B**, **C**, **D1**, **E1**, **F1**, **G1**, **H1**, **K1**, **L1**), posterior (**D3**, **E2**, **F2**, **G2**, **H3**, **K3**, **L3**), ventral (**A2**, **E3**, **K2**), and indet. (**I**, **J**). *atp* anterior process, *artg* articular grove, *dpr* dorsal process, *vpr* ventral process

**Fig. 10:**
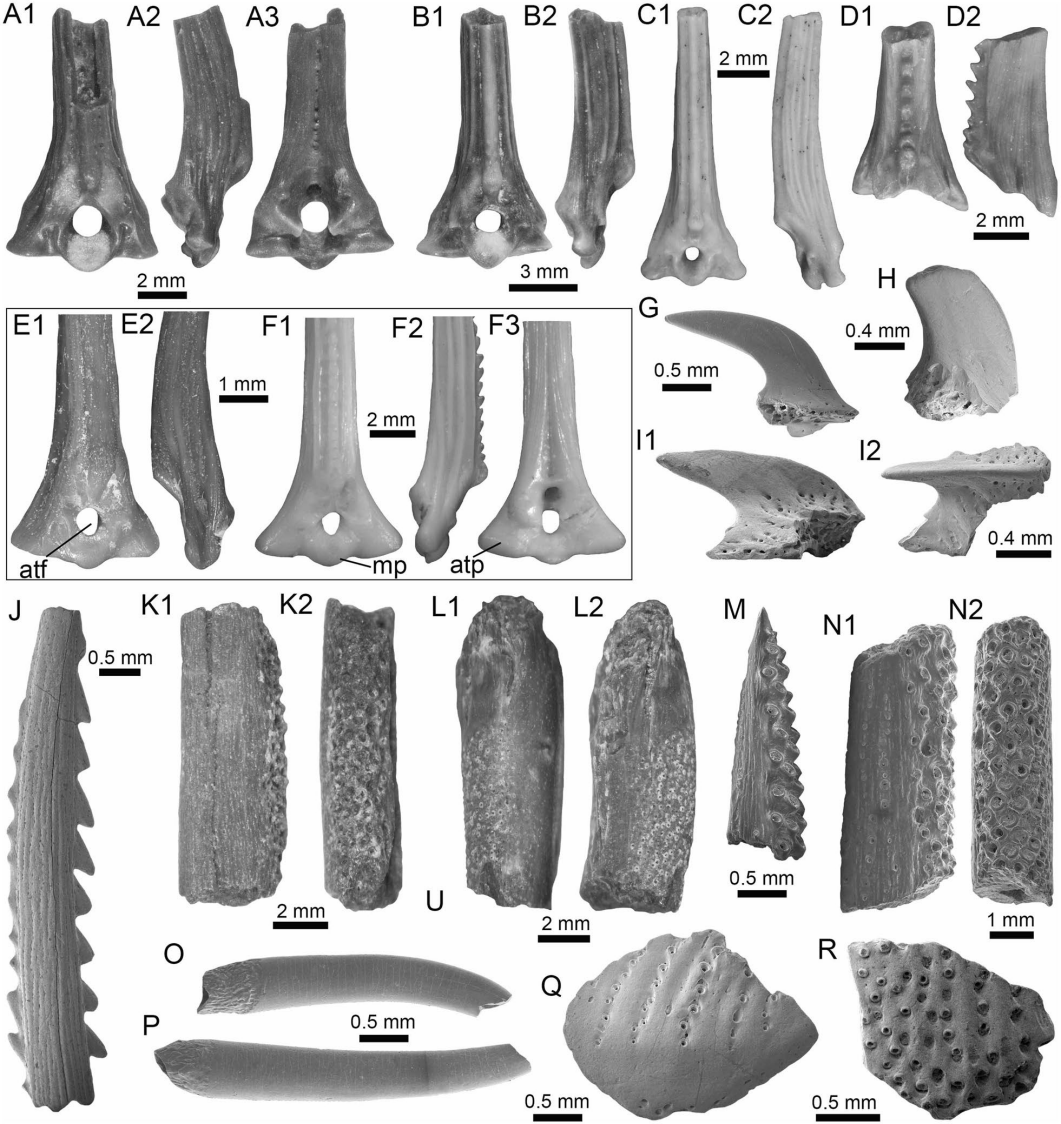
Siluriformes (Doradidae, Heptapteridae, and Loricariidae) from the Vergel Member. **A1**–**D2**, **G**–**I2** Dorsal-fin spines (**A1**–**D2** AMU-CURS-1180) and mid-lateral scutes (**G**, **H** AMU-CURS-864a-b and **I1**, **I2** AMU-CURS-1232a) of Doradidae indet. **E1**–**F3** Dorsal spines of extant *Anadoras wedellii* (**E1**, **E2** AUM-45441) and *Scorpiodoras heckelii* (**F1**–**F3** MHNLS-17959). **J** Pectoral spine of cf. *Pimelodella* sp. (AMU-CURS-1183). **K1**–**P** Pectoral spine fragments (**K1**–**l2** AMU-CURS-1173 and **M**–**N2** AMU-CURS-817), and odontodes (**O**, **P** AMU-CURS-876) of Hypostominae indet. **Q**, **R** Body plates of Loricariidae indet. (AMU-CURS-1230). Views: anterior (**A1**, **B1**, **C1**, **D1**, **E1**, **F1**, **K2**, **L1**, **N2**), lateral (**A2**, **B2**, **C2**, **D2**, **E2**, **F2**, **I2**, **O**–**P**) posterior (**A3**, **F3**), and indet. (**G**–**I1**, **J**, **K1**, **L2**, **M**, **N1**, **Q**–**R**). *atp* anterior process, *atf* articular foramen, *mp* medial process

**Fig. 11:**
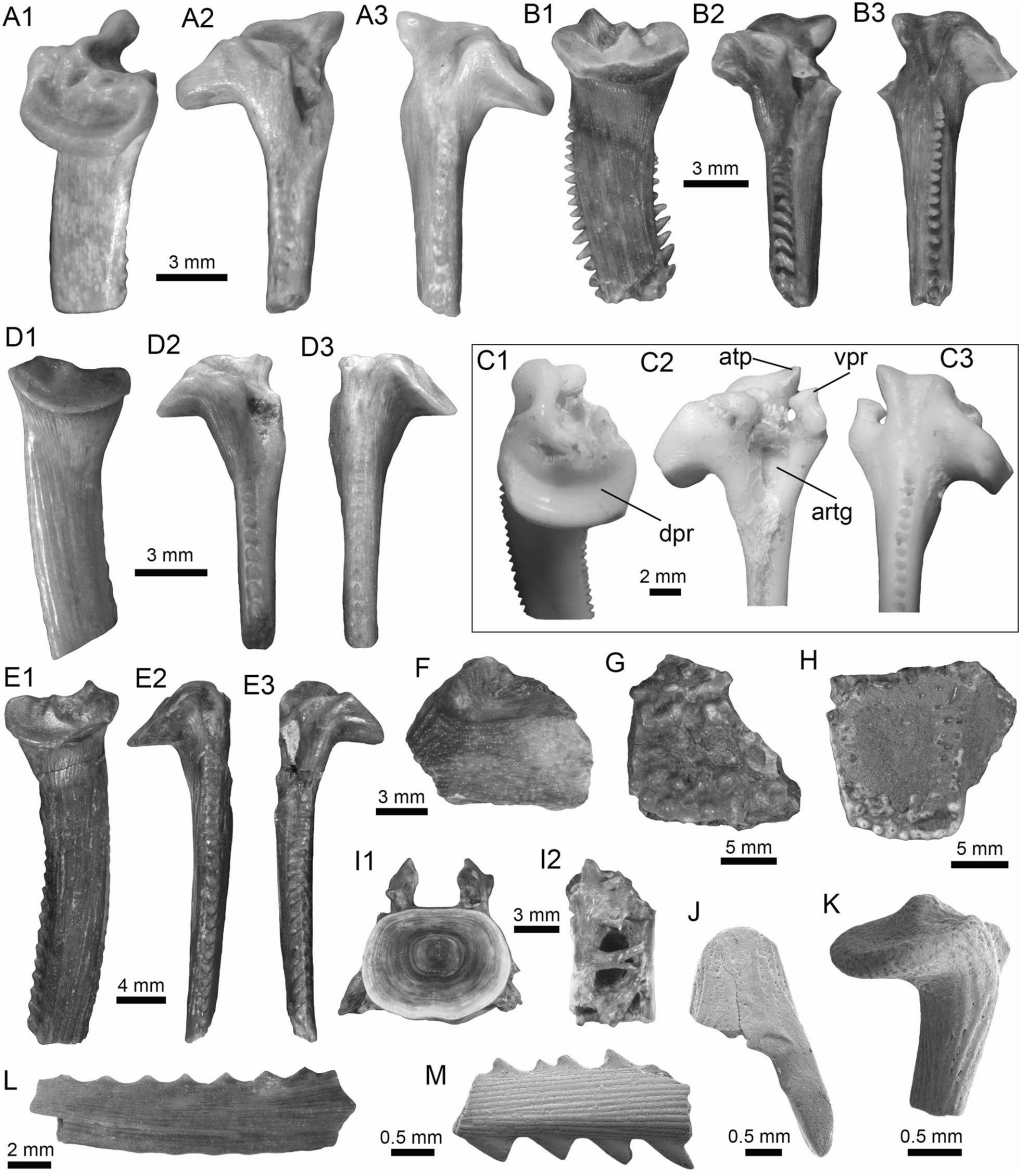
Siluriformes (Pimelodidae and indet.) from the Vergel Member. **A1**–**B3** Left pectoral spines of cf. *Platysilurus* sp. (AMU-CURS-1172). **C1**–**C3** Left pectoral spine of extant *Platysilurus malarmo* (ANSP 187009). **D1**–**E3** Left (**D1**–**D3**) and right (**E1**–**E3**) pectoral spines of Pimelodidae indet. (AMU-CURS-1228). **F**–**M** Partial left cleithrum (**F** AMU-CURS-1237), skull fragments (**G**, **H** AMU-CURS-1175), precaudal vertebra (**I1**, **I2** AMU-CURS-1174), first dorsal spine (**J** AMU-CURS-1181), right pectoral spines (**K** AMU-CURS-867), and pectoral spine fragments (**L** AMU-CURS-867 and **M** AMU-CURS-1229) of Siluriformes indet. Views: anterior (**A3**, **B3**, **C3**, **D3**, **E2**, **I1**, **J**), lateral (**I2**), posterior (**A2, B2**, **C2**, **D2**, **E3**), dorsal (**A1**, **B1**, **C1**, **D1**, **E1**, **F**), anterodorsal (**K**), and indet. (**G**, **H**, **L**, **M**). *atp* anterior process, *artg* articular grove, *dpr* dorsal process, *vpr* ventral process

**Fig. 12:**
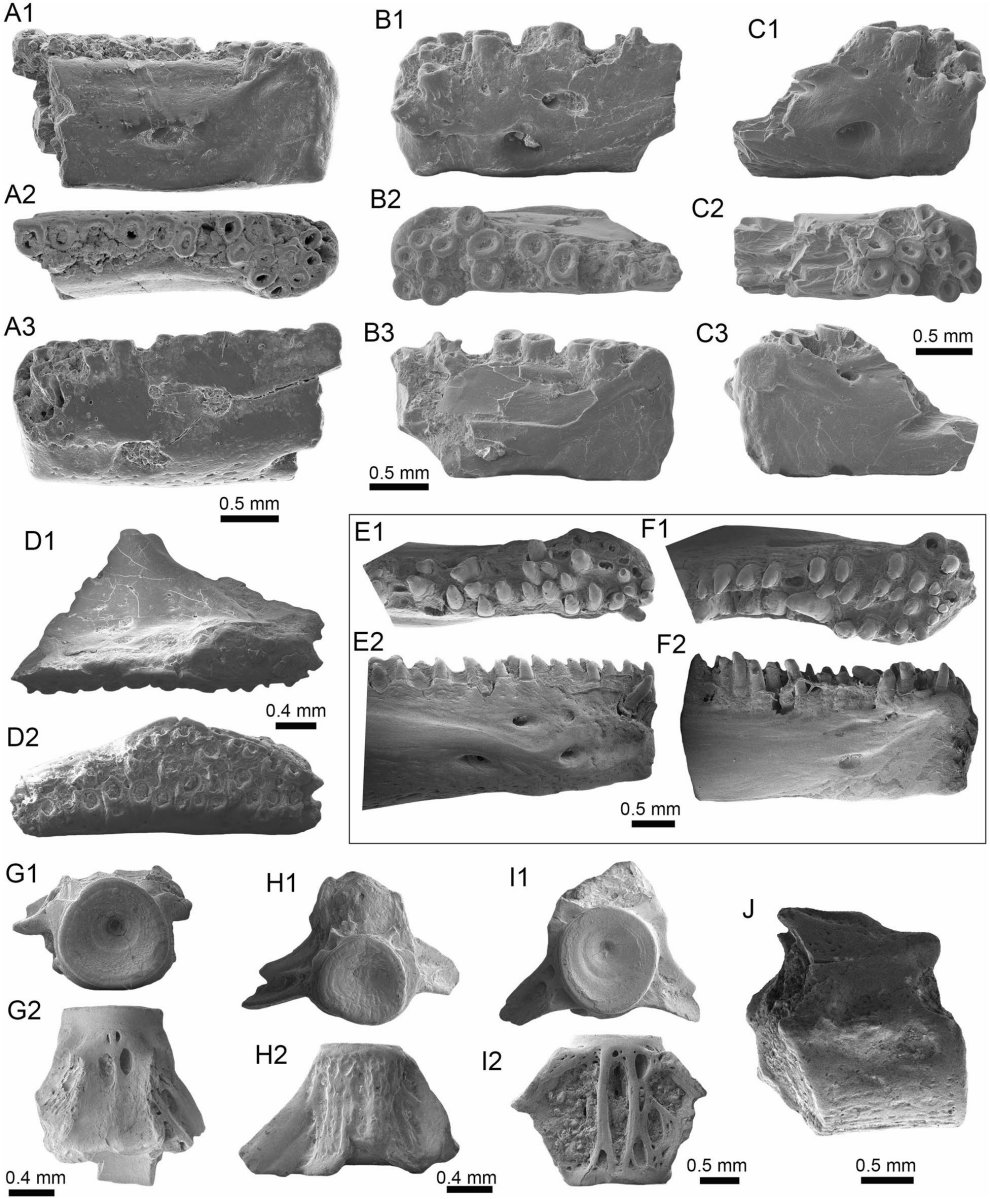
*Synbranchus* sp. from the Vergel Member. **A1**–**C3** Dentaries (right: **A1**–**A3**, **C1**–**C3**, and left: **B1**–**B3**; AMU-CURS-1146-a-c) and branchial bones (**D1**, **D2** AMU-CURS-1146-d). **E1**–**F2** Right (**E1**, **E2**) and left (**F1**, **F2**) dentaries of extant *Synbranchus marmoratus* (PIMUZ A/I 4873). **G1**–**J** Isolated vertebrae of *Synbranchus* sp. (AMU-CURS-1171). Views: anterior (**G1**, **H1**, **I1**), left lateral (**B1**), right lateral (**A1**, **C1**, **E2**), lateral (**D1**, **J**), medial (**A3**, **B3**, **C3**, **F2**), occlusal (**A2**, **B2**, **C2**, **E1**, **F1**), and ventral (**D2**, **G2**, **H2**, **I2**)

**Fig. 13:**
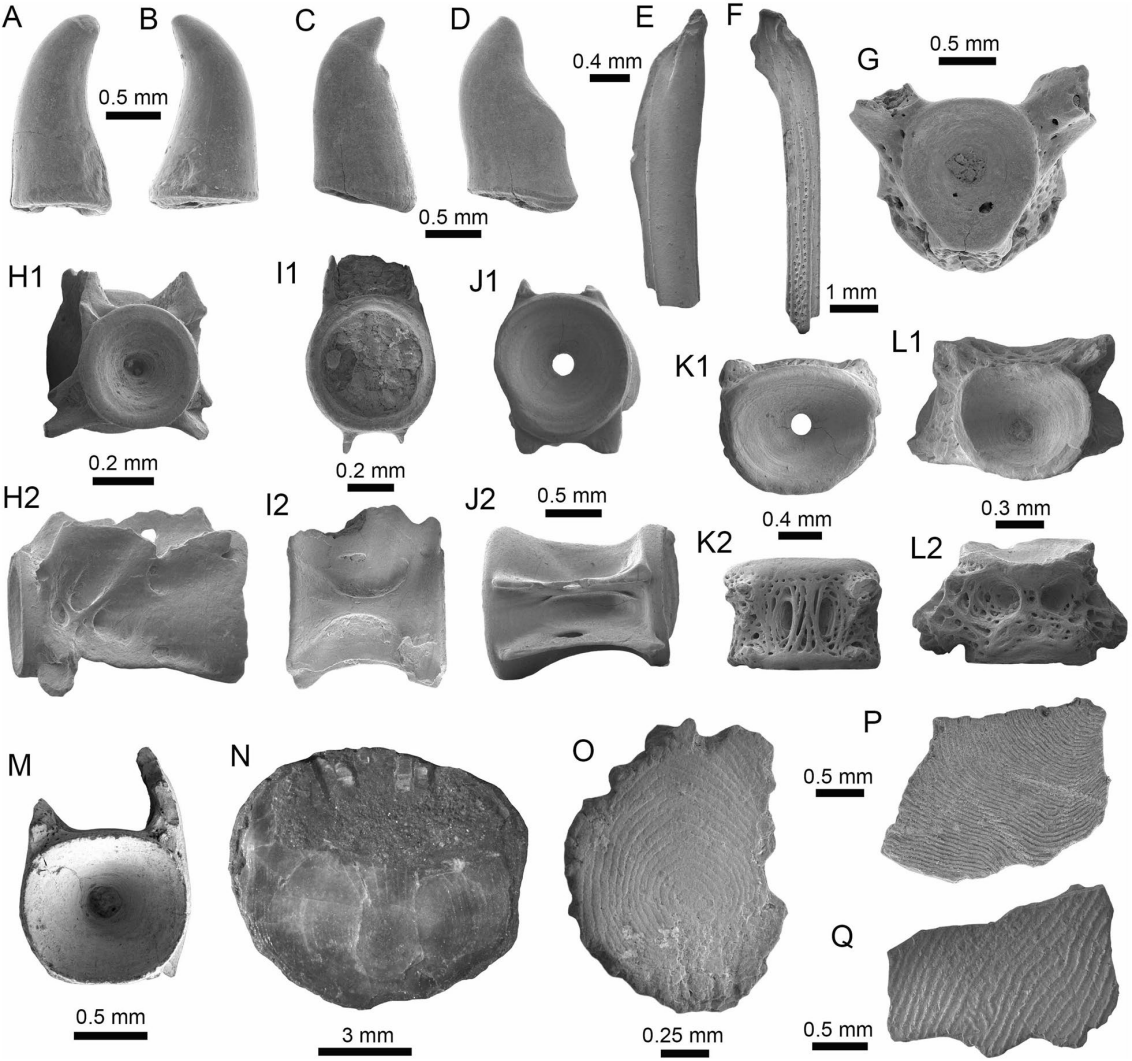
Actinopterygii indet from the Vergel Member. **A**–**D** Pharyngeal (**C**, **D** AMU-CURS-1145) and other teeth of indeterminate position (**A**, **B** AMU-CURS-1178). **E**, **F** Ceratobranchial bones (AMU-CURS-1238). **G**–**M** Isolated vertebrae (AMU-CURS-1177). **N**–**Q** Isolated scales (AMU-CURS-863). Views: anterior (**G**, **H1**, **I1**, **J1**, **K1**, **L1**, **M**), Dorsal (**J2**, **K2**), lateral (**A**–**D**, **H2**, **I2**), ventral (**L2**), and indet. (**N**–**Q**)

**Fig. 14:**
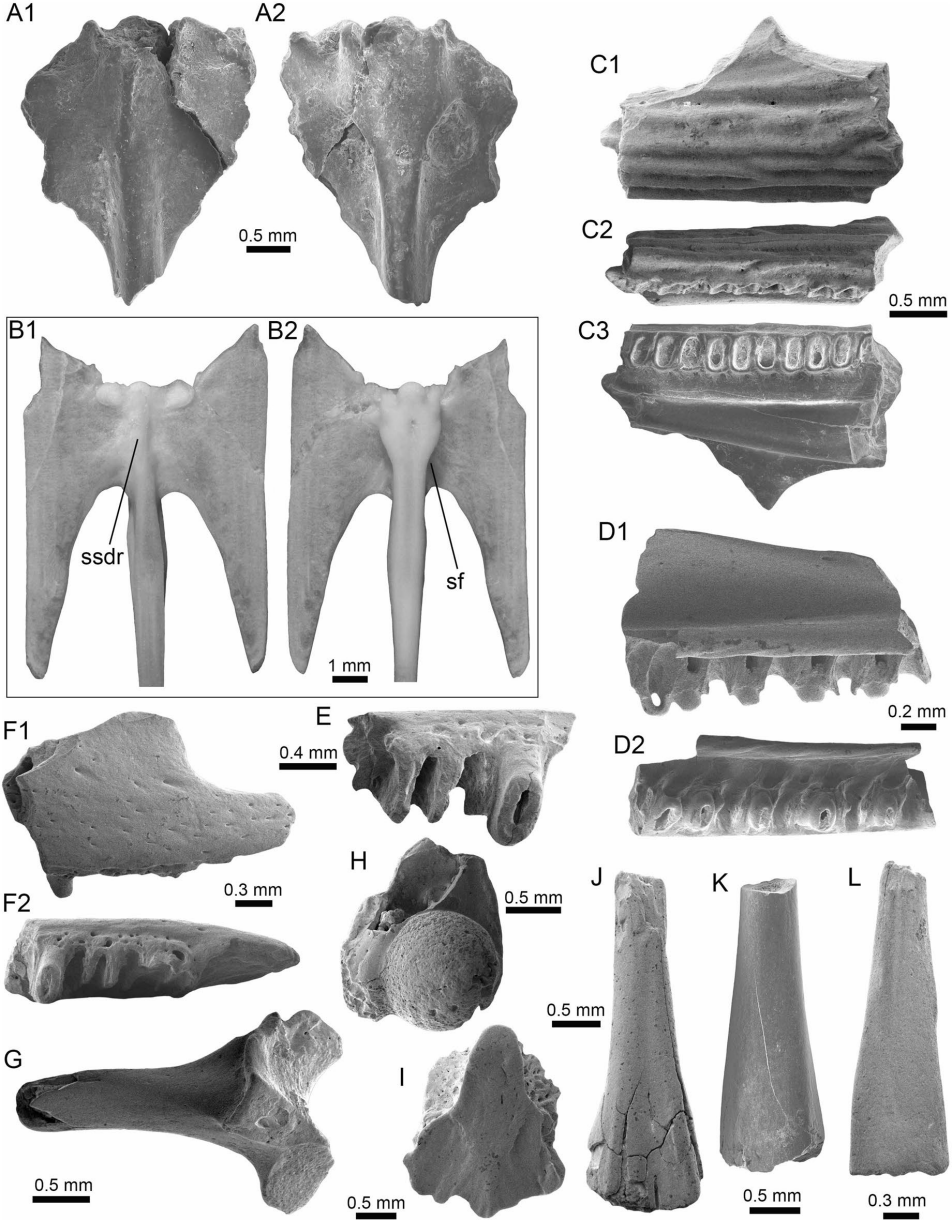
Anura from the Vergel Member. **A1**, **A2** Incomplete fused sacral vertebra of cf. *Pipa* sp. (AMU-CURS-722). **B1**, **B2** Sacral vertebra of recent *Pipa parva* (PIMUZ A/II 118). **C1**–**L** Anura indet. **C1**–**F2** Maxilla fragments (**C1**–**C3** AMU-CURS-1177, **D1**, **D2** AMU-CURS-1149, **E** AMU-CURS-1166 and **F1**, **F2** AMU-CURS-1165). **G** Distal fragment of a left ilium (AMU-CURS-1150). **H** Distal portion of left humerus (AMU-CURS-1152). **I** Spinous process of and incomplete trunk vertebra (AMU-CURS-1153). **J** Distal fragment of a right radioulna (AMU-CURS-1151). **K**, **L** Indeterminate limb bone fragments (AMU-CURS-807). Views: dorsal (**A1**, **B1**, **I**, ?**J**), left lateral (**G**), right lateral (**F1**), lateral (**C1**, **D1**), lateroventral (**C2**), ventral (**A2**, **B2**, **C3**, **D2**, **E**, **F2**, **H**), and indet. (**K**, **L**). *ssdr* sacral sagittal dorsal ridge, *sf* spinal foramen

**Fig. 15:**
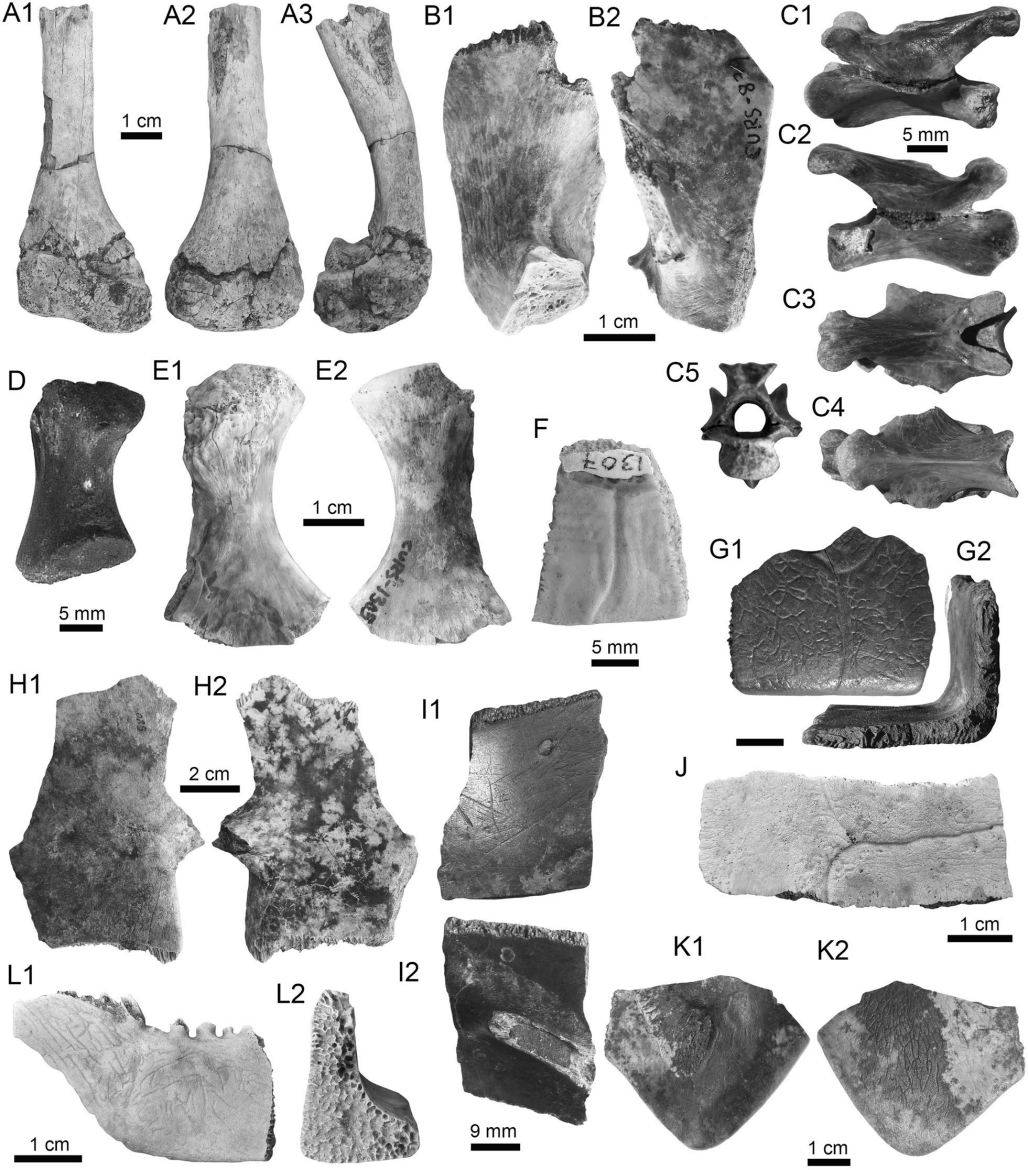
Testudines (Testudinidae, Chelidae, and Podocnemididae) from the Vergel (**A1**–**D**, **G1**–**I2**, **K1**–**L2**) and Cocuiza (**E1**–**F**, **J**) members. **A1**–**A3** Incomplete right femur of *Chelonoidis* sp. (AMU-CURS-584). **B1**, **B2** Anterolateral portion of a left xiphiplastron of *Chelus* sp. (AMU-CURS-839). **C1**–**K2** Podocnemididae indet. **C1**–**C5** Cervical vertebra (AMU-CURS-571). **D** Left ilium (AMU-CURS-675). **E1**, **E2** Left ischium (AMU-CURS-1305). **F** Peripheral bone from the posterior margin of the carapace (AMU-CURS-1307). **G** Peripheral bone from the bridge region of the shell (AMU-CURS-838). **H1**, **H2** Partial right hypoplastron (AMU-CURS-580). **I1**, **I2** Lateral portion of a costal bone, potentially left costal 5 (AMU-CURS-547). **J** Costal bone potentially the right costal 6 (AMU-CURS-1306). **K1**, **K2** Posterior tip of a right xiphiplastron (AMU-CURS-79). **L1**, **L2** Peripheral bone from the carapace-plastron bridge region (AMU-CURS-579). Views: anterior (**A2**), dorsal (**C3**, **E1**, **F**), cross‐sectional (**G2**, **L2**), internal (**B1**, **H2**, **I2**, **K1**), external (**B2**, **H1**, **I1**, **J**, **K2**, **L1**), left lateral (**C1**), right lateral (**A3**, **C2**), lateral (**D**, **G1**), posterior (**A1**, **C5**), and ventral (**C4**, **E2**)

**Fig. 16:**
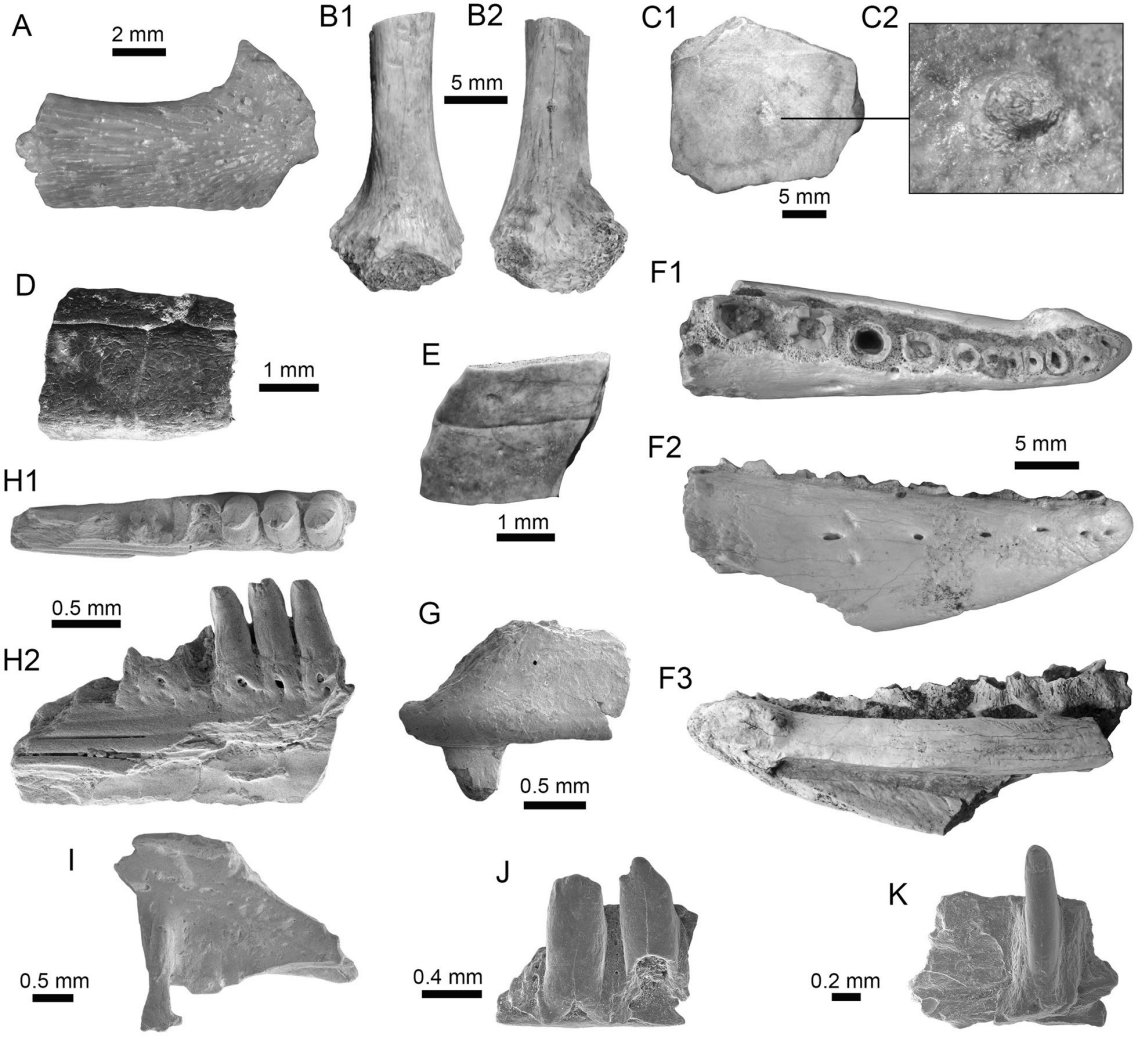
Testudines indet. and (non-snake) Squamata from the Vergel Member. **A**–**E** Testudines indet. **A** Fragment of a right premaxilla (AMU-CURS-1154). **B1**, **B2** Distal section of a left fibula (AMU-CURS-1160). **C1**, **C2** Plastron fragment with a bite mark (AMU-CURS-862). **D**, **E** Shell fragments (**D** AMU-CURS-842 and **E** AMU-CURS-842). **F1**–**F3** Fragmentary right dentary of *Tupinambis* s.l. (AMU-CURS-721). **G**–**K** (Non-snake) Squamata indet. **G** Anterior section of a left maxilla (AMU-CURS-1148). **H1**, **H2** Right jaw fragment preserving three complete teeth (AMU-CURS-1164). **I** Premaxilla fragment with an incomplete tooth (AMU-CURS-1163). **J**, **K** Two maxilla fragments (**J** AMU-CURS-725 and **K** AMU-CURS-797). Views: anterior (**B2**), external (**C1**–**E**), left lateral (**G**), right lateral (**A**, **F2**), mesial (**F3**, **H2**, **I**–**K**), occlusal (**F1**, **H1**), and posterior (**B1**)

**Fig. 17:**
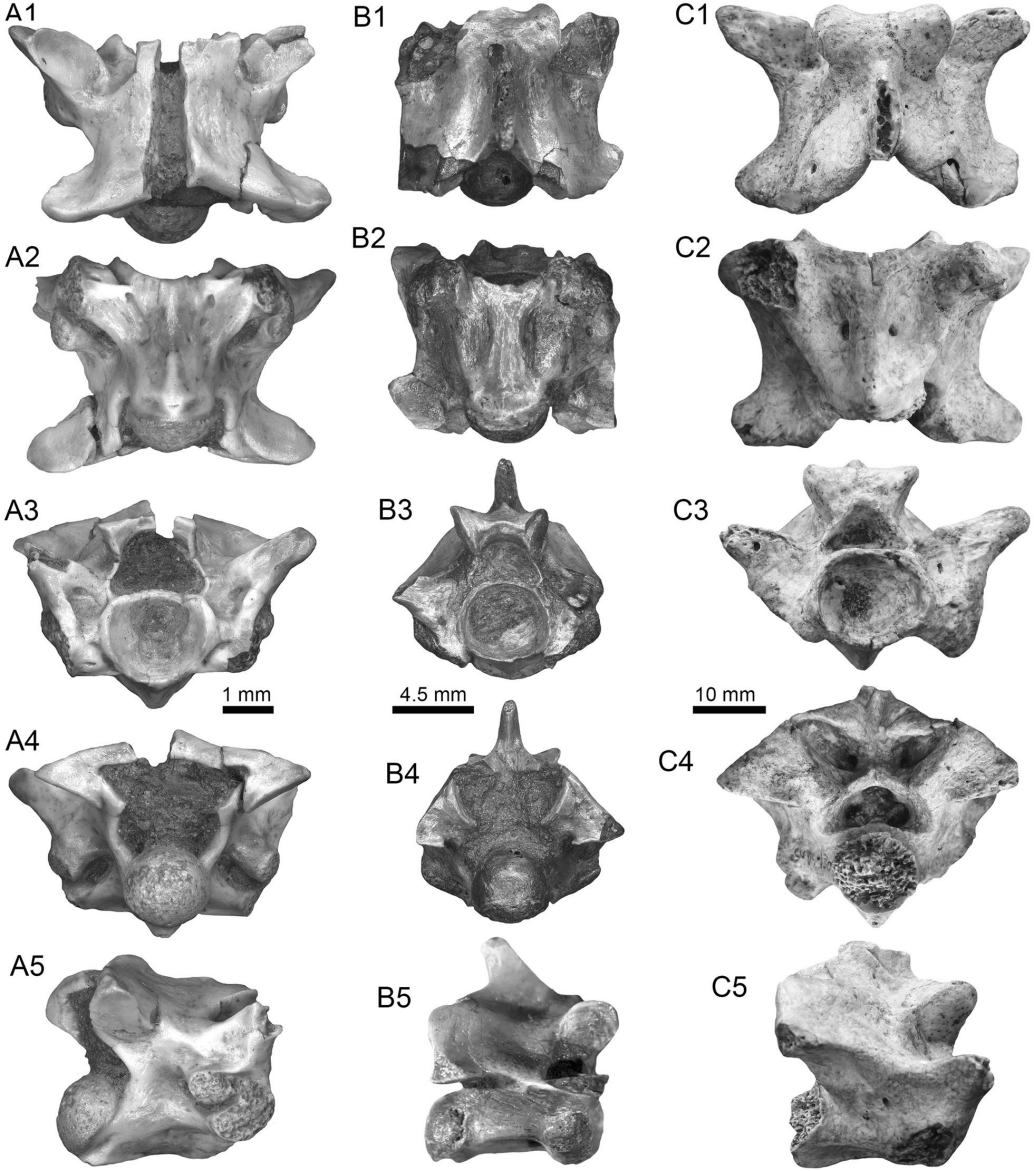
Serpentes (Aniliidae and Boidae) from the Vergel Member. **A1**–**A5** Trunk vertebra of *Anilius scytale* (AMU-CURS-1159). **B1**–**B5** Trunk vertebra of *Corallus* sp. (AMU-CURS-1157). **C1**–**C5** Trunk vertebra of *Eunectes* sp. (AMU-CURS-1304). Views: anterior (**A3**, **B3**, **C3**), dorsal (**A1**, **B1**, **C1**), right lateral (**A5**, **B5**, **C5**), posterior (**A4**, **B4**, **C4**), and ventral (**A2**, **B2**, **C2**)

**Fig. 18:**
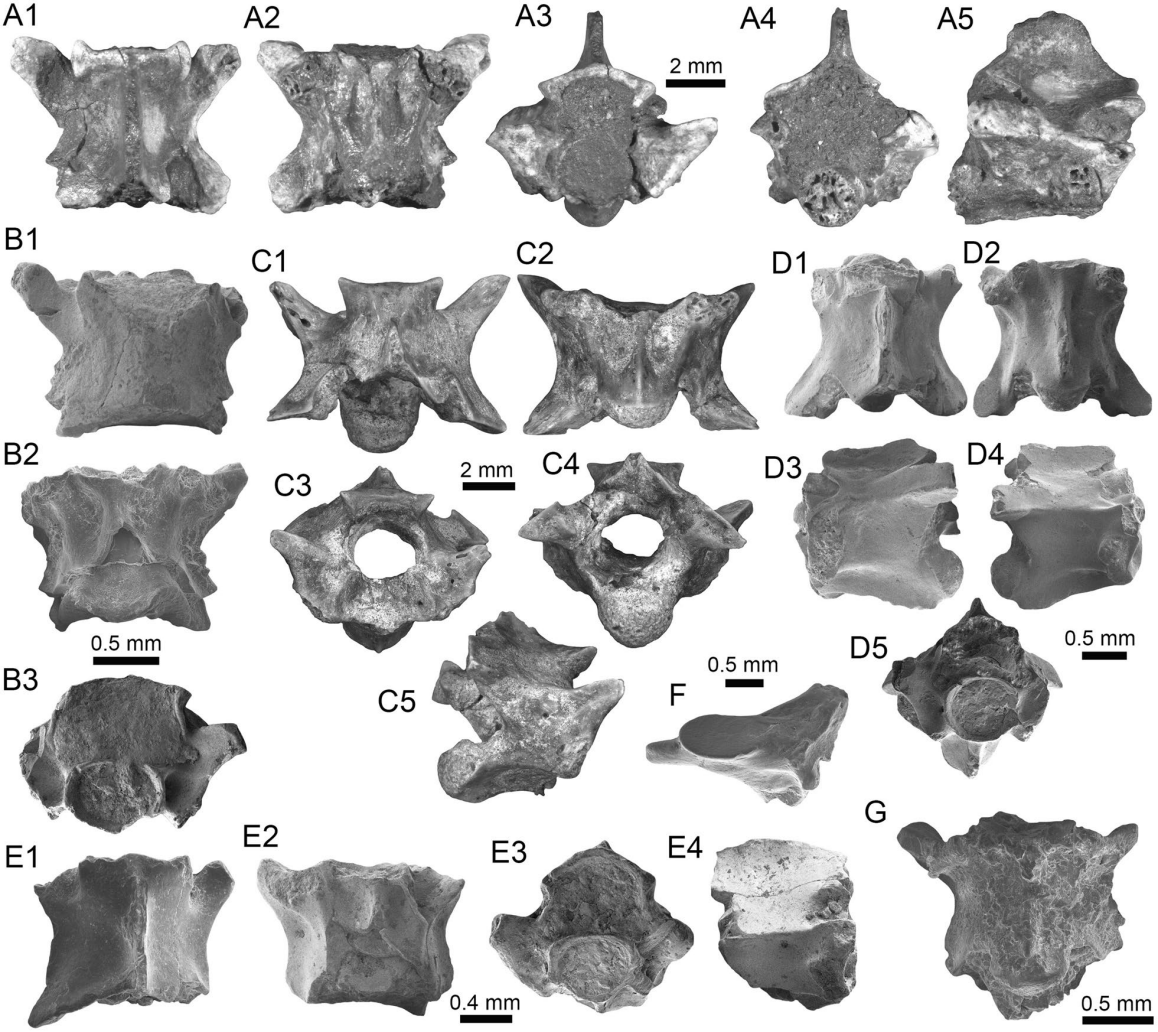
Serpentes (Boidae and Colubroides) from the Vergel Member. **A1**–**B3** Trunk vertebra (**A1**–**A5** AMU-CURS-1147) and specimen of indeterminate position (**B1**–**B3** AMU-CURS-804) of Boidae indet. **C1**–**C5** Trunk vertebra of ?Boidae or ?Aniliidae indet. (AMU-CURS-1158). **D1**–**D5** Trunk vertebra of Colubroides indet. (AMU-CURS-1161). **E1**–**G** Trunk vertebra (**E1**–**E4** AMU-CURS-803), fragmented prezygapophysis (**F** AMU-CURS-724), and a trunk vertebra (**G** AMU-CURS-805) of Serpentes indet. Views: anterior (**A3**, **B3**, **C3**, **D5**, **E3**), dorsal (**A1**, **B1**, **C1**, **D1**, **E1**), left lateral (**D3**), right lateral (**A5**, **C5**, **D4**, **E4**), posterior (**A4**, **C4**), and ventral (**A2**, **B2**, **C2**, **D2**, **E2**, **G**)

**Fig. 19:**
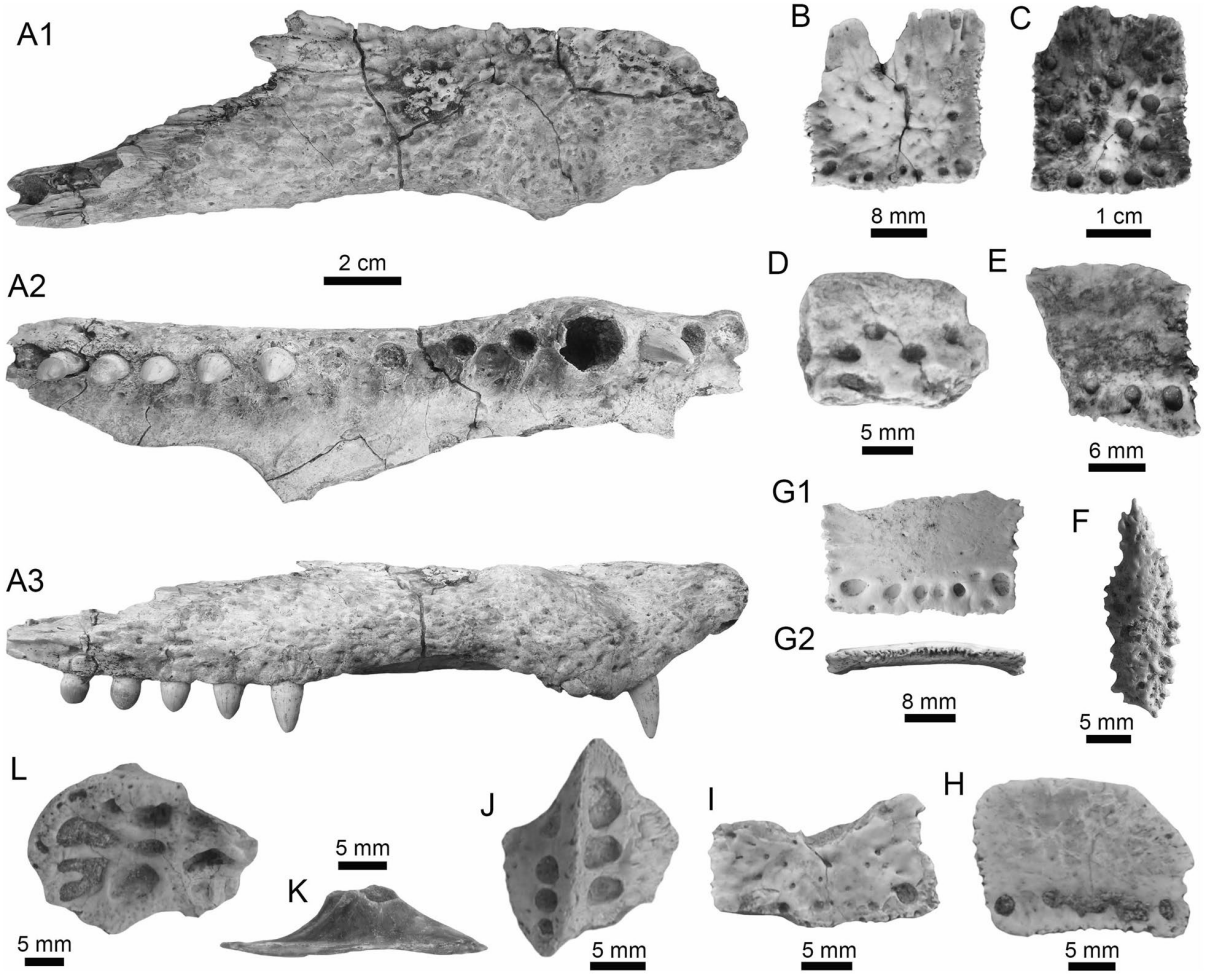
Crocodylia (Caimaninae) from the Vergel (**B**–**F**, **H**–**L**) and Cocuiza (**A1**–**A3**, **G1**, **G2**) members. **A1**–**A3** Right maxilla fragment of *Caiman* aff. *C*. *yacare* (AMU-CURS-1328). **B**–**L** Caimaninae indet. **B**–**J** Osteoderms (**B**–**D** AMU-CURS-553, **E**–**F** AMU-CURS-711, **G1**, **G2** AMU-CURS-1315 and **H**–**J** AMU-CURS-1060a), skull fragment (**L**), and one cervical rib (**K**) (AMU-CURS-1060b). Views: cross sectional (**G2**), dorsal (**A1**, **L**), external (**B**–**G1**, **H**–**J**), lateral (**K**), right lateral (**A3**), and ventral (**A2**)

**Fig. 20:**
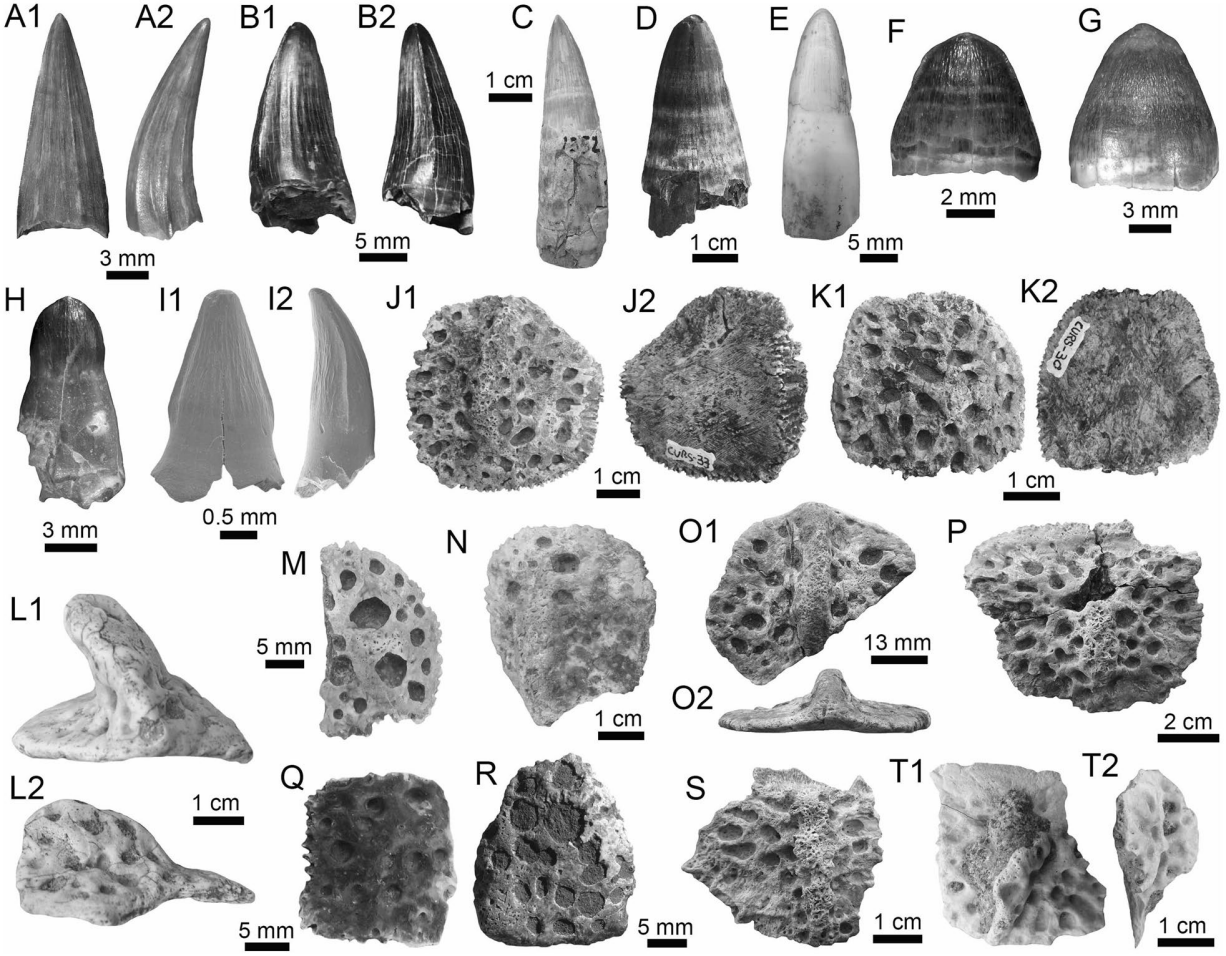
Crocodylia indet. from the Vergel (**A1**, **B2**, **D**, **F**–**N**, **P**–**R**) and Cocuiza (**C**, **E**, **O1**, **O2**, **S**–**T2**) members. **A1**–**I2** Teeth (**A1**, **A2**, **F**–**H** AMU-CURS-861, **B1**, **B2** AMU-CURS-167, **C** AMU-CURS-1352, **D** AMU-CURS-574, **E** AMU-CURS-1322 and **I** AMU-CURS-1095). **J1**–**T2** Osteoderms (**J1**, **J2** AMU-CURS-033, **K1**, **K2** AMU-CURS-030, **L1**, **L2** AMU-CURS-593, **M**, **N**, **Q** AMU-CURS-594, **O1**, **O2** AMU-CURS-1311, **P** AMU-CURS-1184, **R** AMU-CURS-830, **S** AMU-CURS-1313, and **T1**, **T2**-1312). Views: cross sectional (**L1**, **O2**), external (**J1**, **K1**, **M**–**O1**, **P**–**T1**), internal (**J2**, **K2**), labial (**A1**, **C**, **E**–**G**), lateral (**A2**, **B2**, **I2**, **L2**, **T2**), and lingual (**B1**, **D**, **H**–**I1**)

**Fig. 21:**
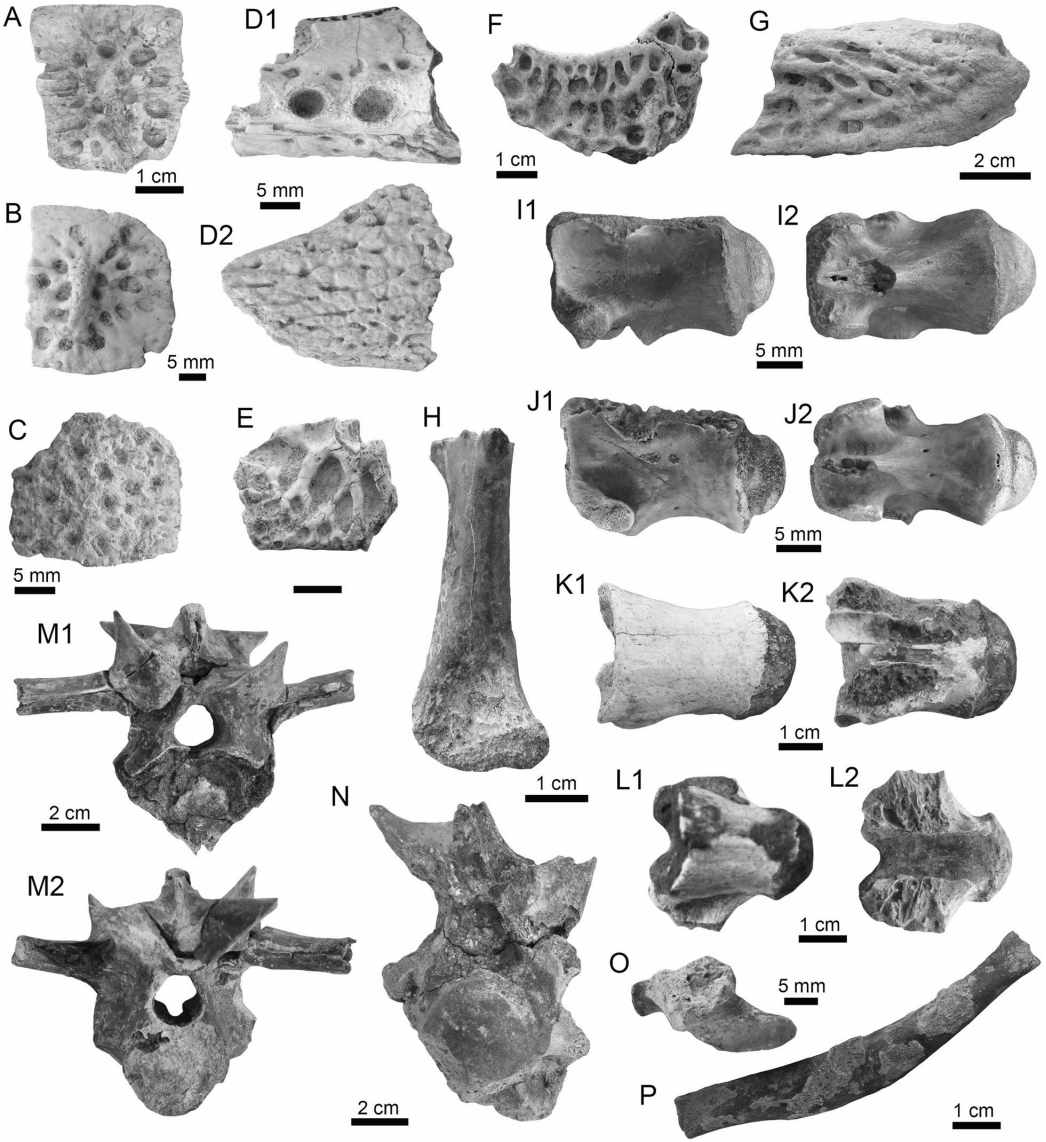
Crocodylia indet. from the Vergel (**A**, **F**, **H**–**P**) and Cocuiza (**B**, **C**, **D1**–**E**, **G**) members. **A**–**C** Osteoderms (**A** AMU-CURS-1029, **B** AMU-CURS-1314, and **C** AMU-CURS-1316). **D1**, **D2** Portion of a left dentary (AMU-CURS-1320). **E** Portion of either skull or lower jaw (AMU-CURS-1321). **F** Fragment from the skull roof (AMU-CURS-1200). **G** Posterior part of a surangular (AMU-CURS-1309). **H** Humeral shaft (AMU-CURS-743). **I1**–**N** Vertebrae (**I1**, **I2** AMU-CURS-21, **J1**, **J2** AMU-CURS-826, **K1**, **K2** AMU-CURS-1030, **L1**, **L2** AMU-CURS-578a and **M1**–**N** AMU-CURS-1062). **O** Isolated prezygapophysis (AMU-CURS-1236). **P** Rib fragment (AMU-CURS-578b). Views: anterior (**M1**), dorsal (**D1**, **F**, **K2**, **L2**, **O**), external (**A**–**C**), left lateral (**D2**, **I1**, **J1**, **G**), posterior (**M2**–**N**), ventral (**H**, **I2**, **J2**–**K1**, **L1**), and indet. (**E**, **P**)

**Fig. 22:**
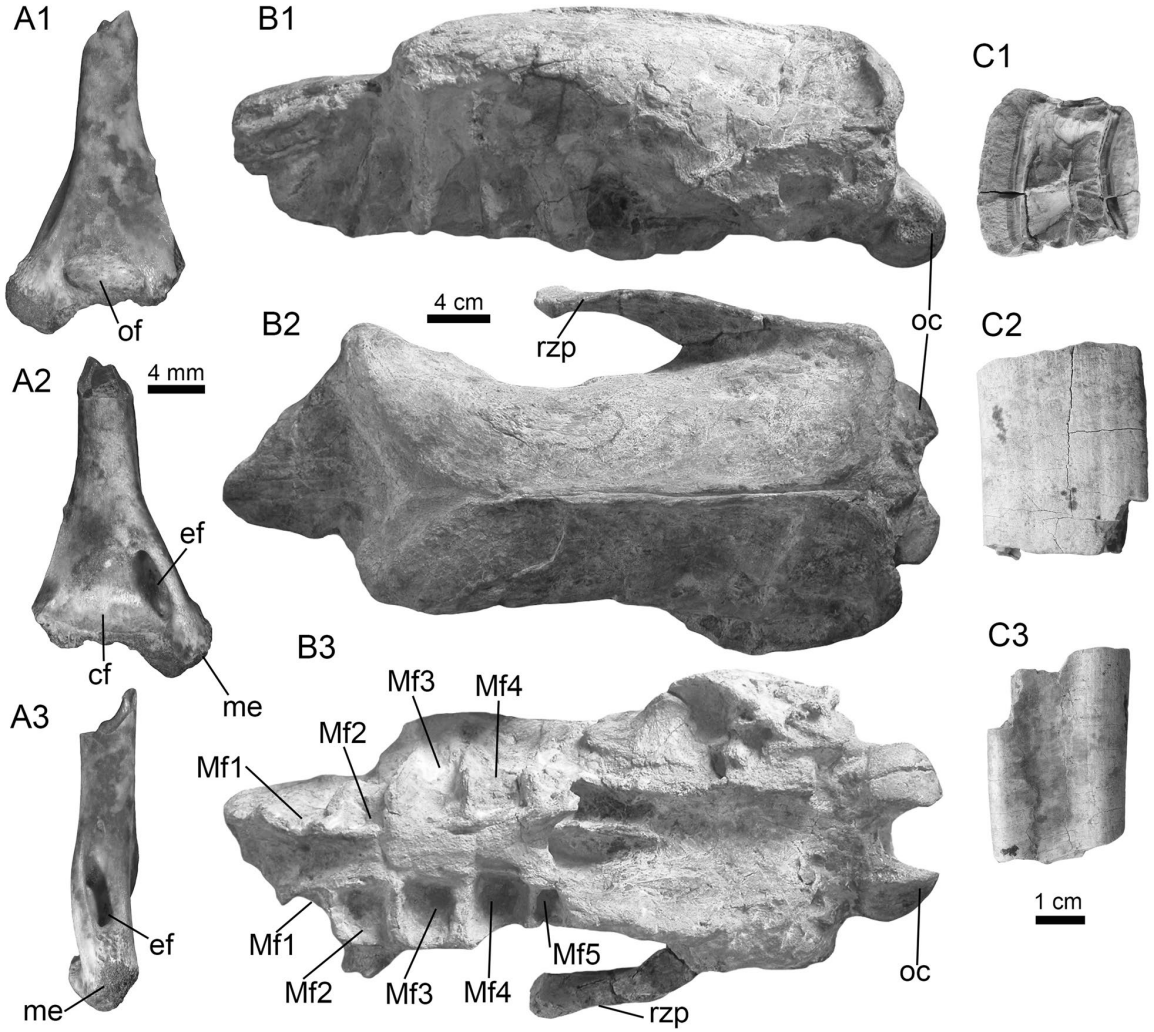
Metatheria (Didelphidae) and Xenarthra (Megatheriidae) from the Vergel (**A1**–**B3**) and Cocuiza (**C1**–**C3**) members. **A1**–**A3** Right humerus (AMU-CURS-1140) of cf. *Didelphis* sp. **B1**–**C3** Skull (**B1**–**B3** AMU-CURS-184) and left molariform Mf3 (**C1**–**C3** AMU-CURS-1303) cf. †*Proeremotherium* sp. Views: anterior (**A2**, **C2**), dorsal (**B2**), labial (**C3**), mesial (**A3**), posterior (**A1**), left lateral (**B1**), occlusal (**C1**), and ventral (**B3**). *cf* coronoid fossa, *ef* entepicondylar foramen, *lc* lateral condyle, *mc* medial condyle, *me* medial epicondyle, *Mf* upper molariform, *oc* occipital condyle, *of* olecranon fossae, *rzp* right zygomatic process

**Fig. 23:**
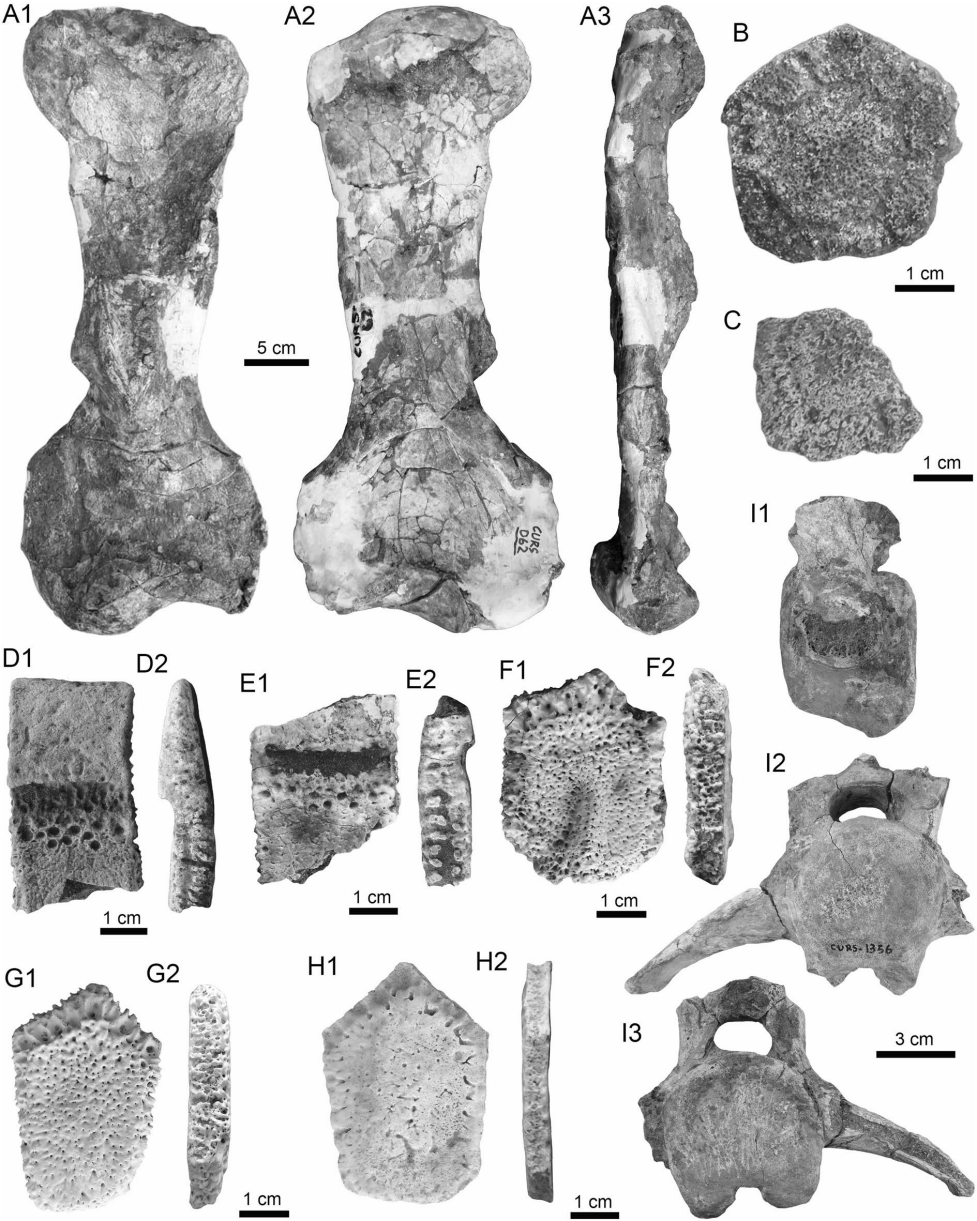
Xenarthra (Mylodontidae, Glyptodontidae, and Pampatheriidae) from the Vergel (**A1**–**E2**, **H1**, **H2**) and Cocuiza (**F1**–**G2**, **I1**–**I3**) members. **A1**–**A3** Right humerus (AMU-CURS-62) of Mylodontidae indet. **B**, **C** Carapace osteoderms (AMU-CURS-1242) of dorsal or postero-dorsal (**B**) and indeterminate position (**C**) of aff. *Boreostemma* sp. **D1**–**G2** Movable osteoderms (**D1**, **D2** AMU-CURS-1063, and **E1**, **E2** AMU-CURS-1119), and fixed osteoderms of pelvic buckler (**F1**, **F2** AMU-CURS-1295, and **G1**, **G2** AMU-CURS-1294) of aff. *Holmesina floridanus*. **H1**, **H2** Fixed osteoderm of pelvic buckler (AMU-CURS-736) of aff. *Plaina* sp. **I1**–**I3** Incomplete caudal vertebra (AMU-CURS-1356) of Xenarthra indet. Views: anterior (**A1**, **I2**), cross sectional (**D2**, **E2**, **F2**, **G2**, **H2**), external (**B**–**D1**, **E1**, **F1**, **G1**, **H1**), left lateral (**I1**), medial (**A3**), and posterior (**A2**, **I3**)

**Fig. 24:**
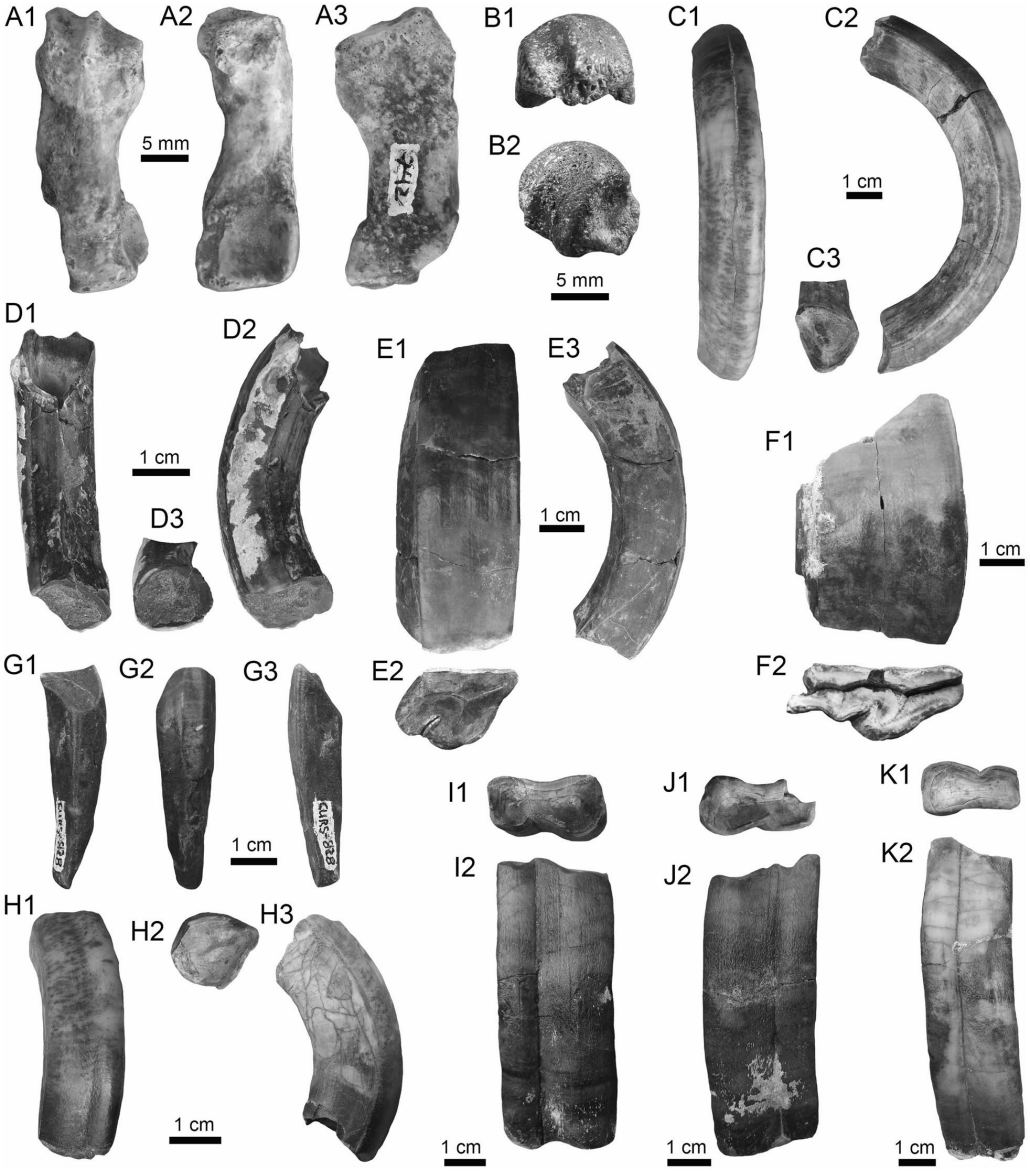
Litopterna (Proterotheriidae) and Notoungulata (Toxodontidae) from the Vergel Member. **A1**–**B2** Metacarpal (**A1**–**A3** AMU-CURS-742) and distal epiphysis of a metacarpal (**B1**, **B2** AMU-CURS-1189) of indeterminate position assigned to Proterotheriidae indet. **C1**–**K2** Teeth of *Falcontoxodon* sp. **C1**–**D3** Upper incisors: **I2** (**C1**–**C3** AMU-CURS-825) and **I1** (**D1**–**D3** AMU-CURS-1335). **E1**–**E3** Right upper premolar P4 (AMU-CURS-1331). **F1**, **F2** Right upper molar M1 or M2 (AMU-CURS-1346). **G1**–**H3** Lower incisors i1 or i2 (**G1**–**G3** AMU-CURS-828 and **H1**–**H3** AMU-CURS-888). **I1**–**K2** Lower premolars left p4 (**I1**, **I2** AMU-CURS-831), right p4 (**J1**, **J2** AMU-CURS-1337), and left p4 (**K1**, **K2** AMU-CURS-1338). Views: anterior (**A1**), distal (**B1**, **E3**), disto-medial (**B2**), medial (**A2**), posterior (**A3**), occlusal (**C3**, **D3**, **E2**, **F2**, **H2**, **I1**, **J1**, **K1**), labial (**E1**, **F1**, **G2**, **H1**, **I2**, **J2**, **K2**), lingual (**D1**, **G1**), mesiolingual (**C2**), and mesial (**C1**, **D2**, **G3**, **H3**)

**Fig. 25:**
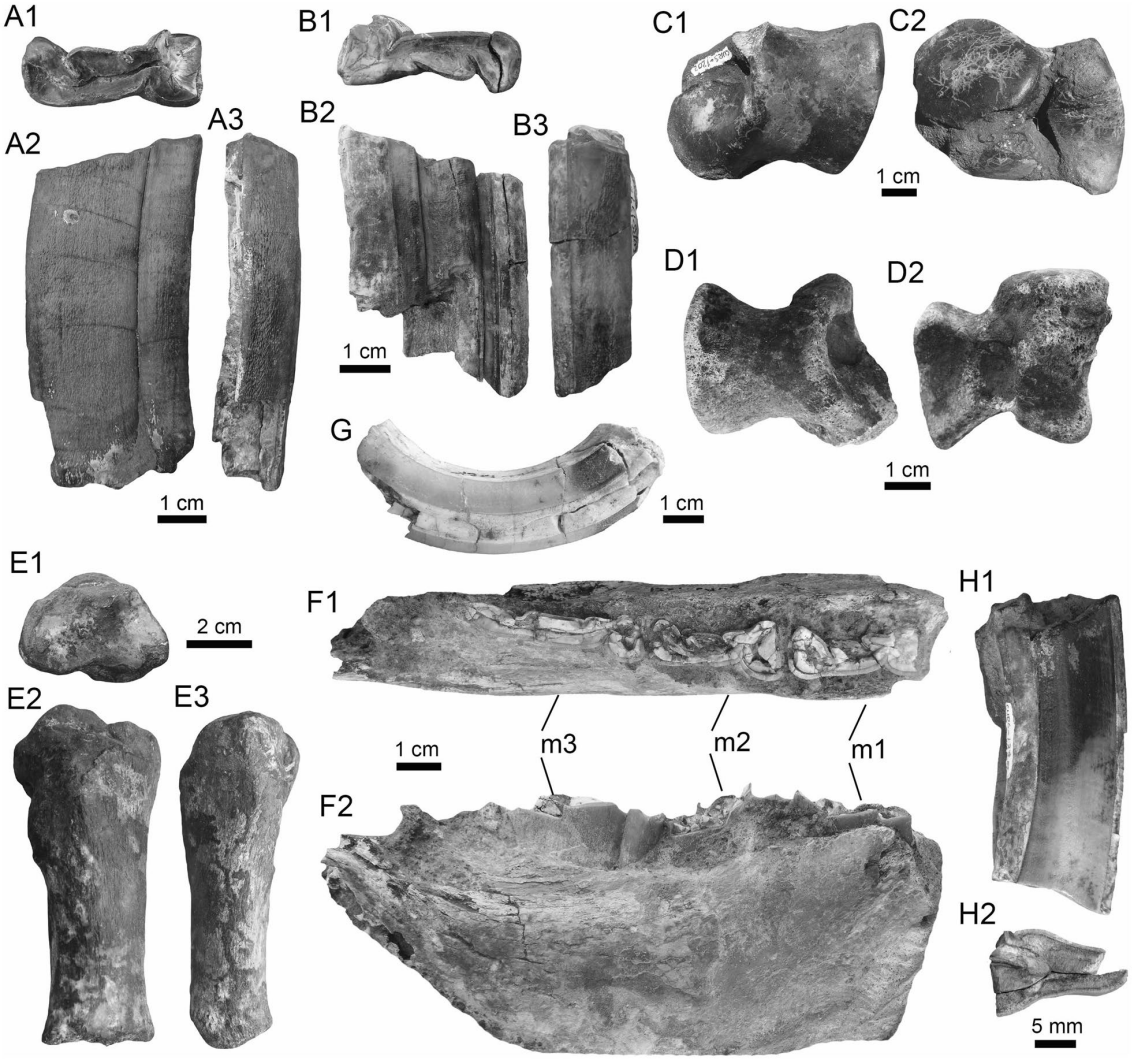
Notoungulata (Toxodontidae) from the Vergel (**A1**–**E3**) and Cocuiza (**F1**–**H2**) members. **A1**–**E3** *Falcontoxodon* sp. **A1**–**B3** Right lower molars, m1 or m2 (**A1**–**A3** AMU-CURS-1345 and **B1**–**B3** AMU-CURS-1348). **C1**–**D2** Astragali. **C1**, **C2** Left astragalus (AMU-CURS-1202). **D1**, **D2** Right astragalus (AMU-CURS-1330). **E1**–**E3** Metatarsal IV (AMU-CURS-1118). **F1**–**H2** Toxodontinae indet. **F1**, **F2** Right partial mandible (AMU-CURS-1351). **G** Upper incisor (AMU-CURS-1326). **H1**, **H2** Upper left molar (AMU-CURS-1325). Views: anterior (**E1**), lateral (**E3**, **F2**), distal (**A3**, **B3**), dorsal (**C1**, **D1**, **E2**), plantar (**C2**, **D2**), labial (**A2**), lingual (**B2**, **G**, **H1**), and occlusal (**A1**, **B1**, **F1**, **H2**)

**Fig. 26:**
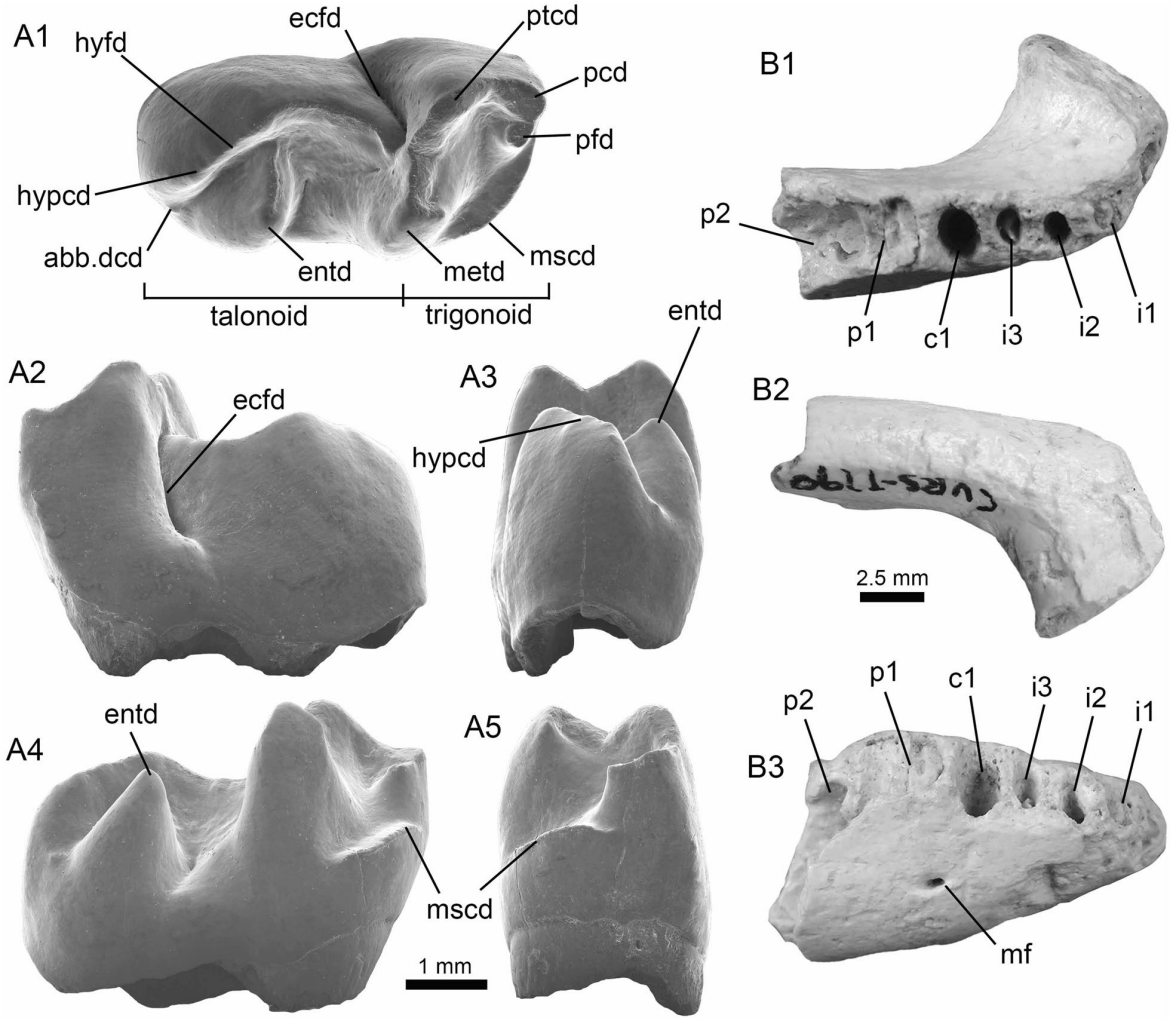
Notoungulata (Typotheria) from the Vergel Member. **A1**–**A5** Left m3 (AMU-CURS-818) of Interatheriidae indet. **B1**–**B3** Jaw with fused symphysis (AMU-CURS-1190) of Typotheria inc. sed. Views: distal (**A3**), dorsal (**B1**), labial (**A2**), lingual (**A4**), mesial (**A5**), occlusal (**A1**), right lateral (**B3**), and ventral (**B2**). *abb.dcd* abbreviated distolingual cingulid, *c* canine, *ecfd* ectoflexid, *entd* entoconulid, *hyfd* hypoflexid, *hypcd* hypoconulid, *i* incisor, *mf* menthal foramen, *metd* metaconid, *pcd* paraconid, *pfd* paralophid, *ptcd* protoconid, *p* premolar, *mscd* mesiolingual cingulid

**Fig. 27:**
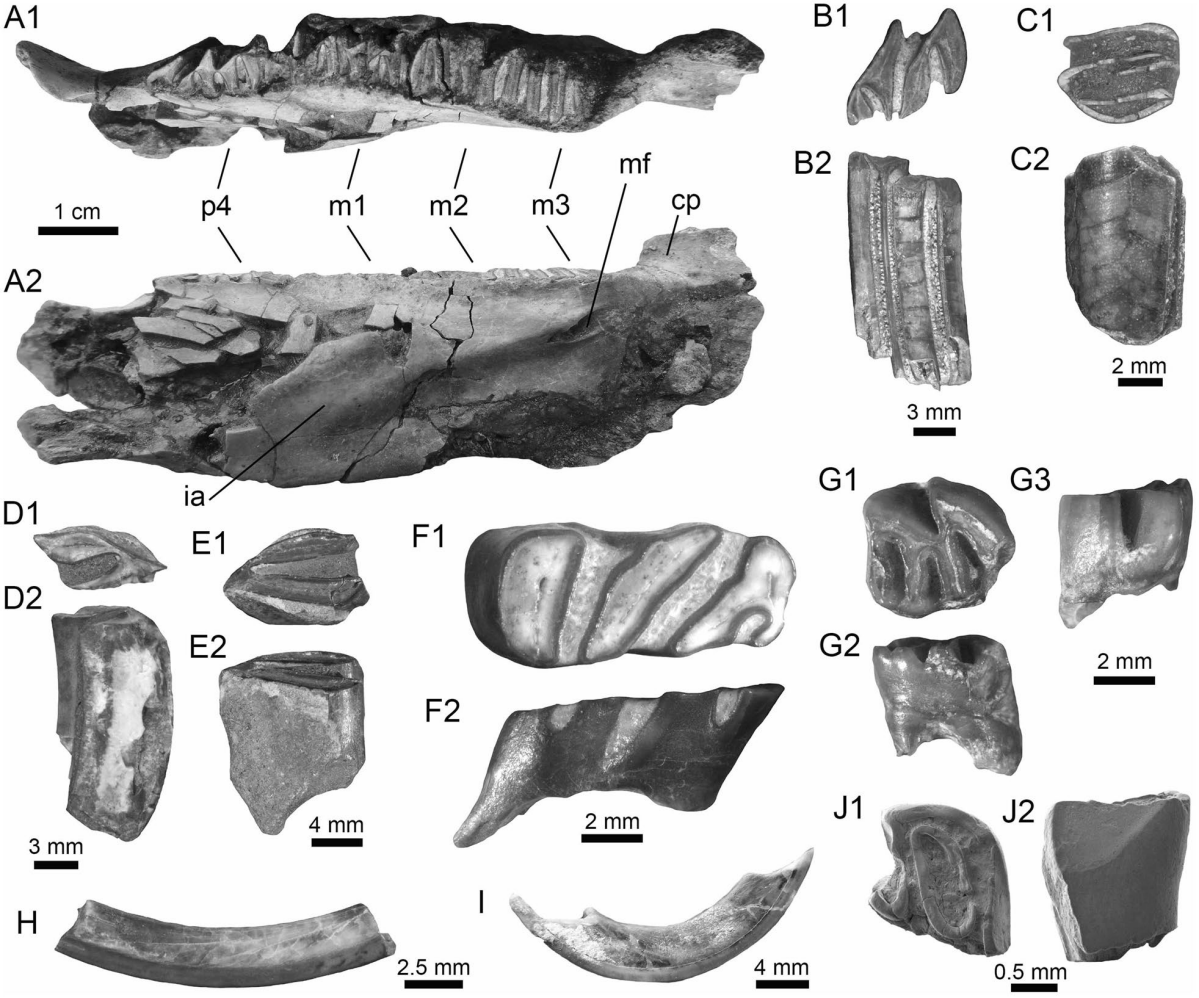
Rodentia (Caviomorpha) from the Vergel Member. **A1**–**B2** Right dentary (**A1**, **A2** AMU-CURS-744) and left molar M1 or M2 (**B1**, **B2** AMU-CURS-1222) of ?*Hydrochoeropsis wayuu*. **C1**–**E2** Fragmented cheek teeth (**C1**, **C2** AMU-CURS-1186, ?pr. IIa’’ and pr. IIb of a right m3; **D1**, **D2** AMU-CURS-1188, ?pr. 1 of a left M1 or M2, and **E1**, **E2** AMU-CURS-1187, ?pr. IIa and pr. IIb’ of left m1) of Hydrochoerinae indet. **F1**, **F2** Left dp4 (AMU-CURS-1220) of Neoepiblemidae indet. **G1**–**G3** Left M1 or M2 (AMU-CURS-1221) of *Marisela gregoriana*. **H**–**J2** ?Caviomorpha indet. **H**, **I** Incisors of indeterminate position (**H** AMU-CURS-1155 and **I** AMU-CURS-1206). **J1**, **J2** Tooth fragment (AMU-CURS-1235). Views: medial (**A2**), distal (**C2**, **D2**, **E2**), labial (**G2**), lingual (**B2**, **F2**, **G3**), mesial or distal (**H**, **I**, **J2**), and occlusal (**A1**, **B1**, **C1**, **D1**, **E1**, **F1**, **G1**, **J1**). *cp* condylar process, *ia* incisor alveolus, *mf* mandibular foramen, *m* lower molar, and *p* lower premolar

**Fig. 28:**
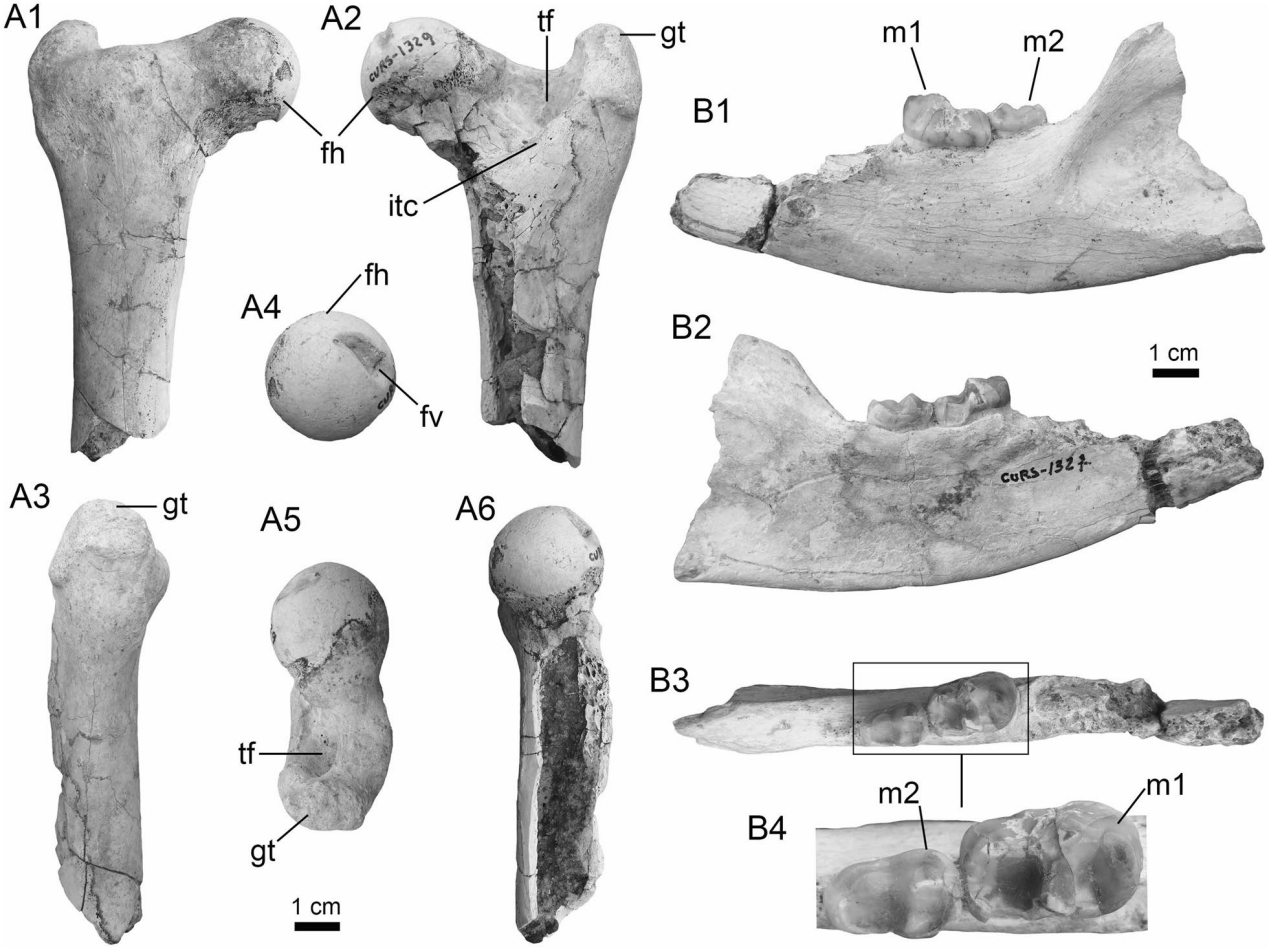
Artiodactyla (Camelidae) and Carnivora (Procyonidae) from the Cocuiza Member. **A1**–**A5** Proximal portion of a right femur (AMU-CURS-1329) of Camelidae indet. **B1**–**B4** Partial left hemimandible (AMU-CURS-1327) of *Chapalmalania* sp. Views: anterior (**A1**), lateral (**A3**, **B1**), proximal (**A5**), medial (**A6**, **B2**), occlusal (**B3**, **B4**), posterior (**A2**). *fh* femoral head, *fv* fovea, *gt* greater trochanter, *itc* intertrochanteric crest, *tf* trochanteric fossa

**Fig. 29:**
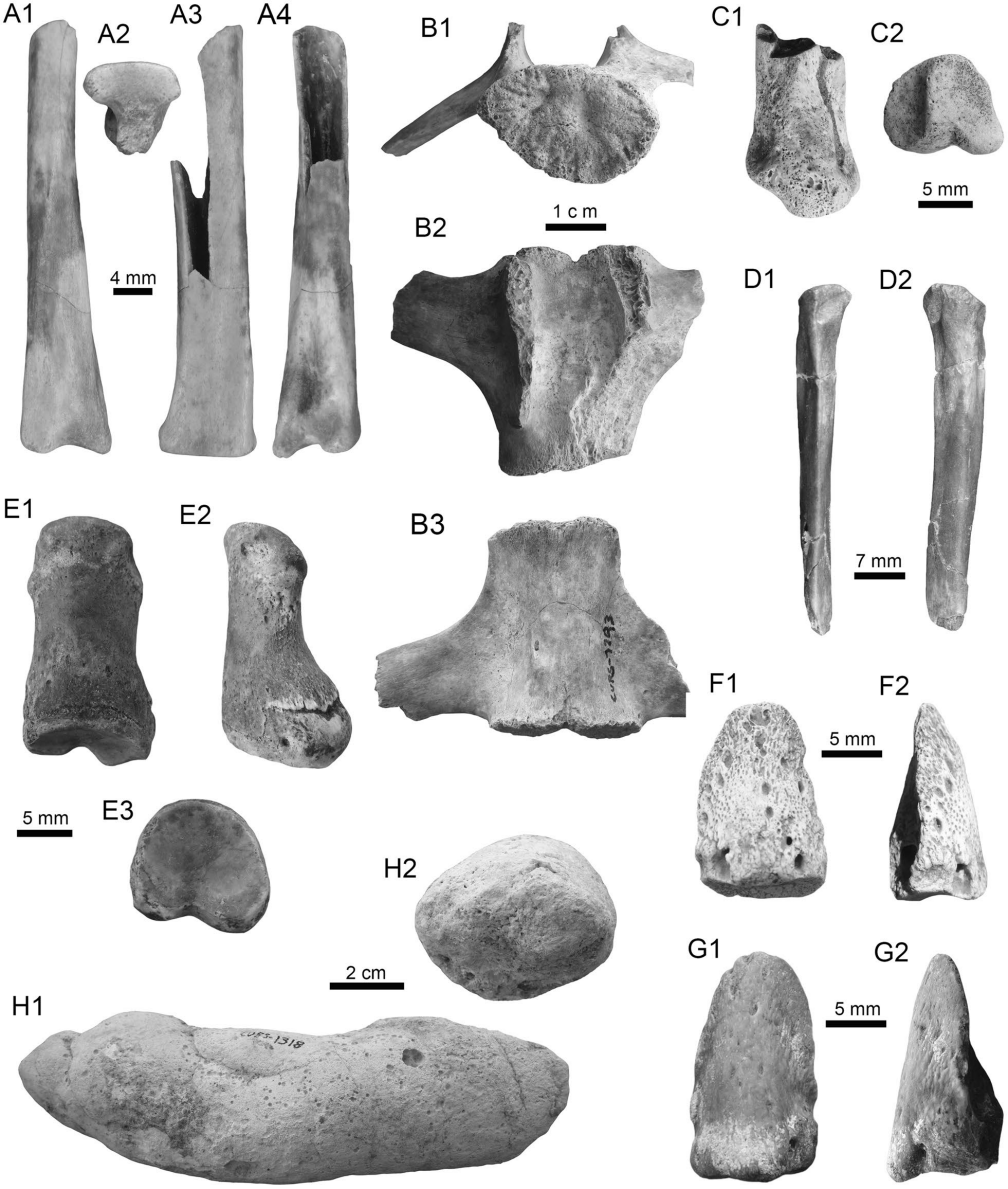
Indeterminate mammalian remains and coprolites from the Norte Casa Chiguaje (**A1**–**A4**, **C1**–**G2**) and San Gregorio Oeste del Pueblo (**B1**–**B3**, **H1**, **H2**) localities. **A1**–**A4** 3rd right metatarsal (AMU-CURS-1096). **B1**–**B3** Incomplete ?caudal vertebra (AMU-CURS-1293). **C1**, **C2** Distal portion of a tibia (AMU-CURS-1198). **D1**, **D2** ?right fibula (AMU-CURS-1197). **E1**–**E3** podial phalange (AMU-CURS-1124). **F1**–**G2** Ungual phalanges (AMU-CURS-1127). **H1**, **H2** Presumed crocodylian coprolite (AMU-CURS-1318). Views: anterior (**A3**, **B1**, **C1**, **D1**), cross sectional (**H2**), dorsal (**B2**, **E1**, **F1**, **G1**), distal (**C2**), lateral (**A1**, **E2**, **G2**, **H1**), medial (**A4**, **F2**), right lateral (**D2**), proximal (**A2**, **E3**), and ventral (**B3**)

The vertebrate fossils correspond to a total sample of 1746 cranial and postcranial elements of freshwater fishes, amphibians, reptiles, and mammals (Figs. 4, 5, 6, 7, 8, 9, 10, 11, 12, 13, 14, 15, 16, 17, 18, 19, 20, 21, 22, 23, 24, 25, 26, 27, 28, and 29, Table 1), recovered from the NCC (11° 17′ 52.9″ N, 070° 14′ 7.3″ W) and SGOP (11° 17′ 54.6″ N, 070° 11′ 24.6″ W) localities. The fossil specimens were collected by the authors (JDCB, RS, TMS, JDC, MD, EAC, AAC, and MRSV), and other collaborators during several field expeditions between 2007 and 2020. Large specimens were surface collected from the outcrops of both localities, while micro-vertebrates and microfossil remains (e.g., seeds and invertebrates) were found only in the NCC locality. A total of ~ 200 kg of conglomerates and sandstones were screen-washed using standard sieves with up to 0.5 mm mesh. The micro-specimens were sorted using a stereomicroscope. Images were captured with a Leica MZ16F multifocal stereomicroscope and a Scanning Electronic Microscope (JEOL JSM-6010). All the fossil specimens are housed in the paleontological collections of the Alcaldía Bolivariana de Urumaco, Falcón State, Venezuela, with the acronym AMU-CURS for vertebrates, and AMU-PB and AMU-PI for fossil plant and invertebrate remains, respectively.

Taxonomic identification involved an extensive bibliographic review and comparison with fossil and extant specimens housed at Argentina [Museo de La Plata (MLP); Museo Argentino de Ciencias Naturales Bernardino Rivadavia, Buenos Aires (MACN); Museo de Ciencias Naturales y Antropológicas J. C. Moyano, Mendoza (MCNAM-PV); Museo Paleontológico Egidio Feruglio, Trelew (MPEF PV); Laboratorio de Investigaciones en Evolución y Biodiversidad-Colección Paleontología de Vertebrados, Universidad Nacional de la Patagonia San Juan Bosco, Esquel, Chubut (LIEB-PV)], Austria [Naturhistorisches Museum, Vienna (NHMW)], Brazil [Museu Paraense Emilio Goeldi (MPEG-V); Paleontological collection of the Departamento Nacional de Produção Mineral, Rio de Janeiro (DNPM); Paleontological collection of the Universidade Federal do Acre (Campus Rio Branco), Rio Branco (UFAC)], Colombia [Instituto de Ciencias Naturales, Universidad Nacional de Colombia (ICN); Museo Geológico José Royo y Gómez, Servicio Geológico Colombiano, Bogotá (IGM); The Mapuka Museum of Universidad del Norte (MUNSTRI)], France [Muséum National d'Histoire Naturelle, Paris (MNHN)], Hungary [Hungarian Natural History Museum, Budapest (HNHM)], Italy [Massimo Delfino Herpetological Collection, Università di Torino (MDHC)], Poland [Institute of Systematics and Evolution of Animals of the Polish Academy of Sciences, Krakow (ZZSiD)], Spain [Museo Nacional de Ciencias Naturales, Madrid (MNCN)], Switzerland [Natural History Museum of Basel (NMB); Paleontological Institute and Museum at the University of Zurich (PIMUZ)], United Kingdom [Natural History Museum, London (NHMUK)], USA [Academy of Natural Sciences of Drexel University, Philadelphia (ANSP); American Museum of Natural History, New York (AMNH); California Academy of Sciences, San Francisco (CAS); Field Museum of Natural History, Chicago (FMNH); Florida Museum of Natural History, Gainesville (FLMNH); Natural History Museum Los Angeles County (LAM), Vertebrate collection of the La Brea Tar Pits and Museum, Los Angeles, California], and Venezuela [Centro de Investigaciones Antropológicas, Arqueológicas y Paleontológicas of the Universidad Experimental Francisco de Miranda (CIAAP, UNEFM-PF); Colección de Paleontología del Instituto Venezolano de Investigaciones Científicas (IVIC); Museo de Ciencias de Caracas (MCNC); Museo de Biología de la Universidad del Zulia (MBLUZ); Museo de la Estación Biológica Rancho Grande (EBRG); Museo de Historia Natural La Salle (MHNLS), Fundación La Salle de Ciencias Naturales, Caracas].

In order to estimate the generic richness and assess the completeness of the sampling, we performed a rarefaction and extrapolation sampling curve (Colwell et al. 2012). Rarefaction curves were made for the two best sampled clades (mammals and fishes) recorded in the NCC locality. We estimated generic richness because most of the specimens could not be identified to species level. We included specimens identified to genus level, and if a specimen was only identified to a more inclusive clade, we treated it as a different genus. For example, as Mylodontidae is not represented by any genus, a specimen was referred as a mylodontid undetermined genus (Table 1). We computed and plotted the rarefaction and extrapolation sampling curves using the iNEXT package (Hsieh et al. 2016) available in R (R core team 2019).

## Dating of the San Gregorio Formation

We analyzed 30 palynological samples (Additional file 3) from two stratigraphic columns spanning the entire San Gregorio Formation, including the NCH (Figs. 1A and 3A; Additional file 1) and SGRS (Fig. 1; Additional file 2) sections, plus 29 samples from the Cocuiza Member for micropaleontology (nannoplankton and foraminifera) (Additional file 4). Five marine low-Mg calcitic shell molluscan fossils (pectinids) from the NCH section and four from the Rio Seco Oil Pipeline section (Cocuiza Member) were prepared for ^87^Sr/^86^Sr geochronological analyses (Additional file 5).

The palynological samples were prepared following standard techniques at Instituto de Investigaciones en Estratigrafía (IIES), Universidad de Caldas, Colombia (Traverse 2007). The procedure included the processing of 15 g of rock in hydrochloric acid (HCl) for 12 h to remove calcareous material. Hydrofluoric acid (HF) was then added to remove silicates. The organic residue was cleaned using ultrasonic equipment, and then concentrated by centrifugation, followed by mounting of a first cover slide in a solution of polyvinyl alcohol. A second cover slide was mounted following the same protocol described above after oxidation of the residue with nitric acid (HNO3). Canadian balsam was used to seal both mounted slides.

A total of 29 samples were prepared for stratigraphic purposes using the standard technique of smear slides (Backman and Shackleton 1983). Microscopic examinations of calcareous nannofossils were performed in a Nikon light microscope at 1000× magnification. We apply the standard biozonation of Martini (1971) for tropical areas and use the biochronology proposed by Backman et al. (2012).

Twenty-two rock samples were prepared for foraminifera (Thomas and Murney 1985). Samples were treated with hydrogen peroxide (H_2_O_2_) [5%] to eliminate the organic matter. Each sample was washed trough a 63-µm sieve to remove the finest sediment. The residue was dried at 50 °C in an oven for 24 h, and separated into the following fractions: > 63 µm, > 125 µm, > 250 µm, and > 425 µm. Fractions were analyzed using a stereomicroscope Nikon SMZ 1500. Most of the foraminifera extracted from the sediment were benthic, and our taxonomic classification followed van Morkhoven et al. (1986), Bolli et al. (1994), Kaminski and Gradstein (2005), and Holbourn et al. (2013).

Powdered calcite samples were drilled from the interior of each specimen using a hand-held Dremel tool with a carbide dental burs. Approximately 0.01 to 0.03 g of powder was recovered from each fossil sample, and these were analyzed according to standard techniques (Kirby et al. 2008). The powdered samples were dissolved in 100 µL of 3.5 N HNO_3_ and then loaded onto cation exchange columns packed with strontium-selective crown ether resin (Eichrom Technologies, Inc.) to separate Sr from other ions. Sr isotope analyses were performed on a Micromass Sector 54 Thermal Ionization Mass Spectrometer equipped with seven Faraday collectors and one Daly detector in the Department of Geological Sciences at the University of Florida. Sr was loaded onto oxidized tungsten single filaments and run in triple collector dynamic mode. Data were acquired at a beam intensity of about 1.5 V for 88Sr, with corrections for instrumental discrimination made assuming ^86^Sr/^88^Sr = 0.1194. Errors in measured ^87^Sr/^86^Sr are better than ± 0.00002 (2*σ*), based on long-term reproducibility of NIST 987 (^87^Sr/^86^Sr − 0.71024). Age estimates were determined using the appropriate portion of Look-Up Table Version 4:08/03 associated with the Sr isotopic age model of McArthur et al. (2001).

## Results

### Dating of the San Gregorio Formation

*Vergel Member*: Most of the pollen samples were sterile (22 out of 30, Additional file 3). The pollen record of meters 237–262 of NCH section, which corresponds to the top 40 m of the Vergel Member (Additional file 1), includes the last occurrence datum (LAD) of *Bombacacidites nacimientoensis*, *Retitrescolpites*? *irregularis*, and *Rhoipites guianensis*.

**Fig. 30:**
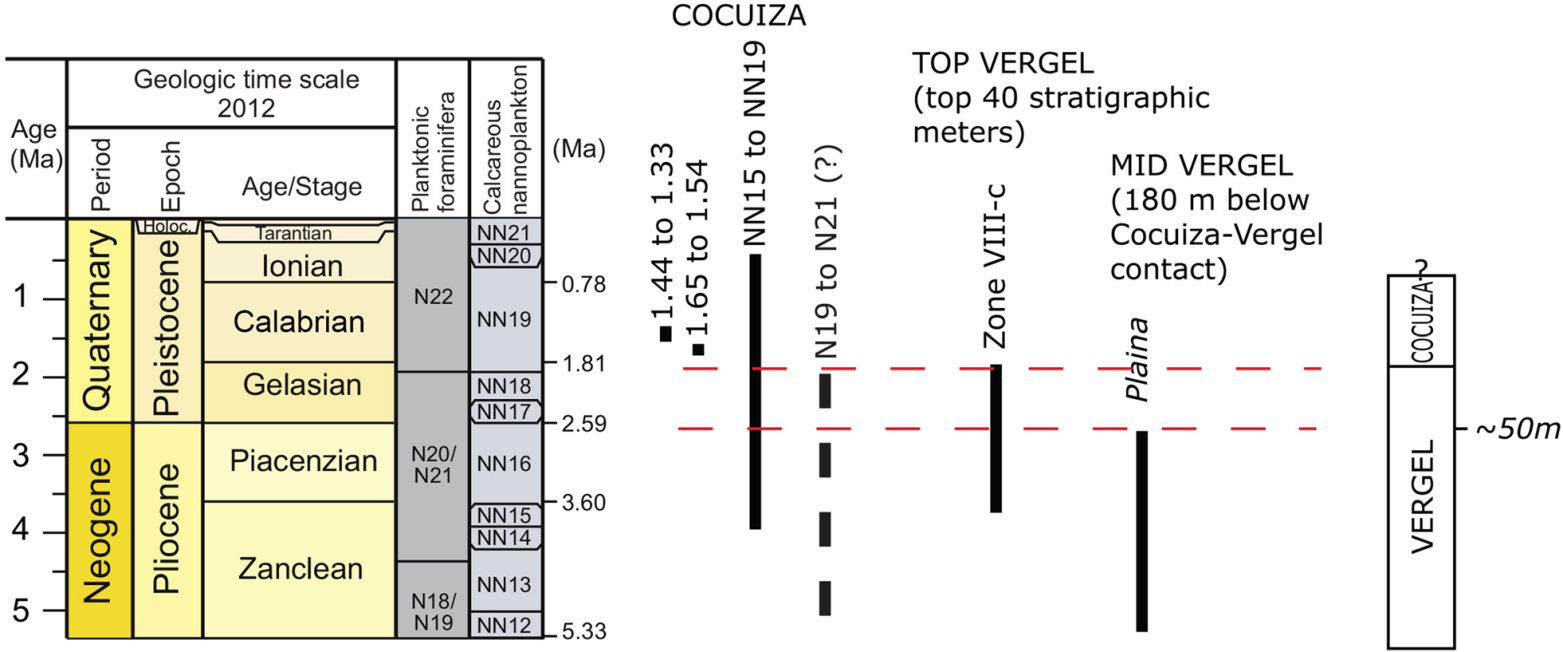
New chronostratigraphy for the San Gregorio Formation. The age (Ma, million years ago) is derived from multiple palynological, nannoplankton, and foraminifera and ^87^Sr/^86^Sr analyses (see Additional files 3, 4, 5)

*Cocuiza Member*: Strontium ratios ranged between 0.709100 and 0.709342 (Additional file 5). Five of the samples yielded ratios higher than, or indicative of, modern seawater suggesting diagenetic alteration. Two others yielded latest Pleistocene ages, but were statistically inseparable from modern seawater. Two samples that seemed pristine and did not show evidences of probable alteration yielded Early Pleistocene ages when compared to the global seawater ^87^Sr/^86^Sr curve for the Neogene (McArthur et al. 2001). The first, from NCH section, yielded a mean age of 1.38 ± 0.06 Ma. The other, SGRS section, yielded a mean age of 1.59 ± 0.05 Ma (Additional file 5) (Fig. 30).

Calcareous nannoplankton results show poor to moderate preservation and abundant Paleogene and Neogene reworked microfossils. The youngest identified assemblage is composed of *Calcidiscus macintyrei*, *Helicosphaera sellii*, *Gephyrocapsa* spp., and *Pseudoemiliania lacunosa*. Of the twenty-two samples analyzed for foraminifera, eight were barren. Foraminifera were poorly preserved and relative abundances were low, mainly composed of benthic taxa. A consistent assemblage is composed of calcareous benthic *Ammonia beccarii*, *Elphidium poeyanum*, and *Melonis barleeanum* (Additional file 4). There are poorly preserved planktonic foraminifera, including *Globoturborotalita* cf. *woodii* and *Globoturborotalita* cf. *rubescens* (Additional file 4). The assemblage also contains *Rhabdammina cylindrica* and *Gyroidinoides complanatus* from the early to middle Miocene or older (Bolli et al. 1994; Kaminski and Gradstein 2005) that could be considered as reworked.

### Paleodiversity and taxonomy

**Fig. 31:**
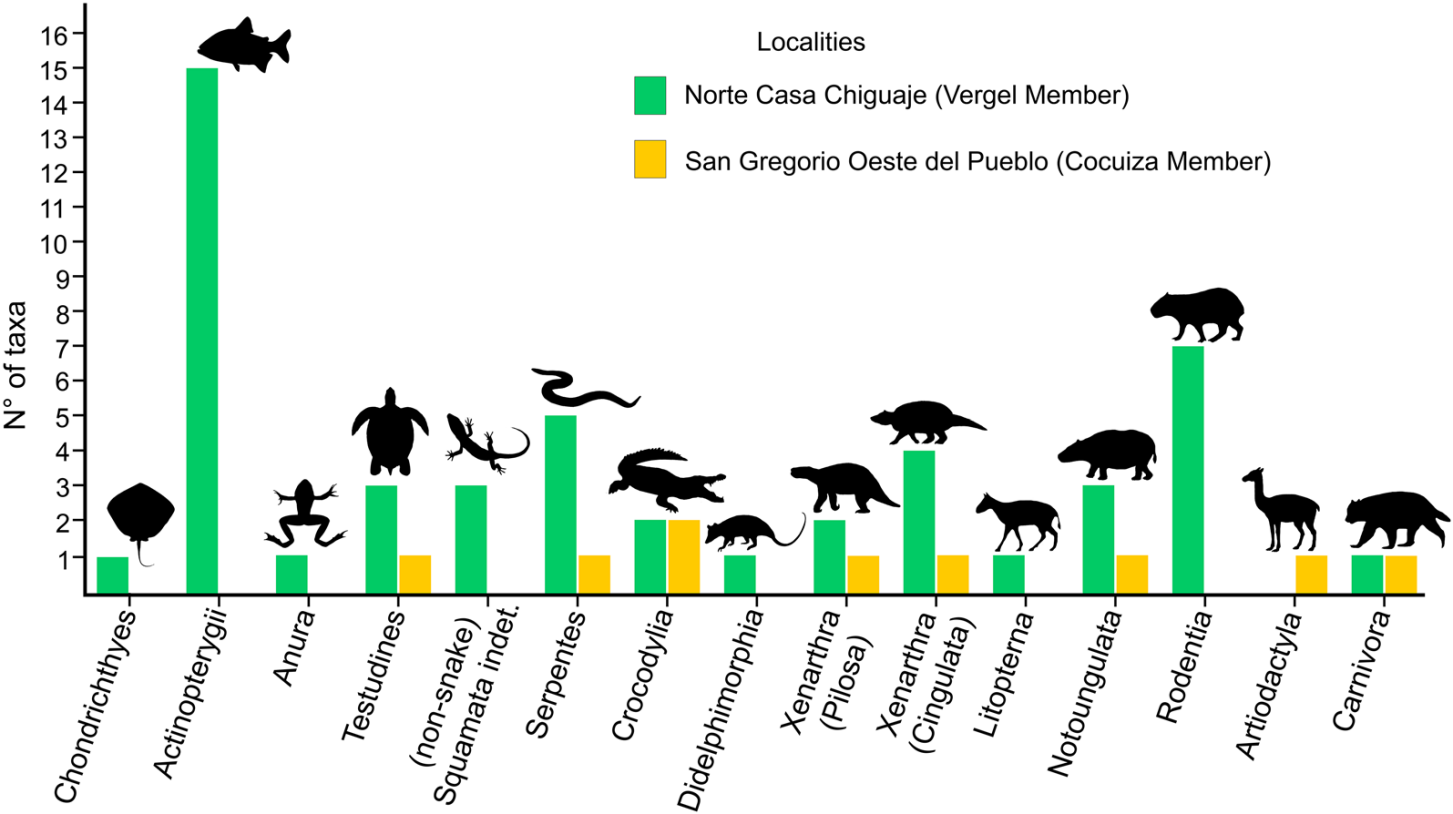
Freshwater and terrestrial vertebrate diversity of the San Gregorio Formation by localities. Information based on Table 1

A total of 1746 cranial and postcranial specimens represent the fossil sample from the San Gregorio Formation, 1719 specimens from the NCC locality (Vergel Member), while 27 are from SGOP locality (Cocuiza Member). Herein a terrestrial and freshwater faunal diversity of at least 49 taxa (fishes, amphibians, reptiles, and mammals) is reported from NCC (Fig. 31), including taxa previously described from the locality (Carlini et al. 2008c, 2018; Carlini and Zurita 2010; Vucetich et al. 2010; Zurita et al. 2011; Scheyer et al. 2013; Castro et al. 2014; Forasiepi et al. 2014; Carrillo et al. 2018). The terrestrial and freshwater vertebrate diversity reported for the first time from the SGOP locality includes nine reptilian and mammalian taxa (Fig. 31).

Chondrichthyes Huxley, 1880

Batomorphii Cappetta, 1980

Myliobatiformes Compagno, 1973

Potamotrygonidae Garman, 1877

*Potamotrygon* Garman, 1877

*Potamotrygon* sp.

(Fig. 4A1–J).

*Locality*: NCC (conglomerate, Fig. 3B).

*Material*: Fifty-two isolated teeth of indeterminate jaw position (AMU-CURS-868–875, -1094 and -1212) and 36 fragmented caudal spines (AMU-CURS-865).

*General description, comparisons and remarks*: The teeth are small, less than 2.4 mm wide, and 2 mm high. The crown is higher than the root, with a middle transverse crest that separates the labial and lingual sides. Two crown shapes are recognized, one with a cusp and the other without one. In the cuspidate teeth, the crest is lingually elongated and forms a distinctive triangular cusp; the labial side of most specimens is concave. In non-cuspidate teeth, the crown looks wider than long, with both rounded and concave labial sides. We identify the presence of teeth with cuspidate (males) or non-cuspidate (females) crowns, as the sexual dimorphism in adult individuals of the freshwater stingray *Potamotrygon* (see Adnet et al. 2014). With the exception of only two cuspidate teeth (Fig. 4G1–H3), remaining teeth are ornamented on their labial side. All specimens have a strongly convex lingual side. The root is a typical holaucorhize type, with two lobes having both rounded and flattened basal sections; a central foramen is present in the nutritive grove.

The caudal spines are eroded and broken (Fig. 4I, J); however, most of the specimens preserve their denticles, as well as the central ridge and central groove in the ventral and dorsal sides, respectively. The morphological characters present in the teeth and caudal spines from the NCC locality coincide with those present in *Potamotrygon* (e.g., Adnet et al. 2014 and references there in). Nevertheless, due to the scarce comparative material and the poor knowledge of the broader dental pattern among more than 30 recognized living *Potamotrygon* species from different South American river basins, we refrain from a more specific allocation.

Myliobatiformes indet.

(Fig. 4K1–K3).

*Locality*: NCC (conglomerate, Fig. 3B).

*Material*: An isolated vertebra (AMU-CURS-1213).

*General description, comparisons and remarks*: The vertebra is small with an amphicoelous centrum 3.5 mm in diameter and 2.7 mm wide. A pair of dorsal and ventral foramina where the neural and hemal arches were nested are preserved. The dorsal and ventral foramina are elongated and not well defined, a diagnostic feature of ray vertebrae (see Kozuch and Fitzgerald 1989). Although the only batoid recorded until now in the San Gregorio Formation is *Potamotrygon*, the vertebra does not preserve diagnostic elements to assign it to that taxon.

Actinopterygii Klein, 1885

Characiformes (sensu Fink and Fink, 1981)

Anostomidae Günther, 1864

*Megaleporinus* Ramirez et al., 2017

cf. *Megaleporinus* sp.

(Fig. 5A1–B3).

*Locality*: NCC (conglomerate, Fig. 3B).

*Material*: Two premaxillary symphyseal teeth (AMU-CURS-851).

*General description, comparisons and remarks*: Premaxillary symphyseal teeth of up to 3.9 mm in height and 2 mm wide. Both teeth are straight, with a massive unicuspid incisiform crown, a deep concave labial side, and a rounded and slightly irregular cutting edge. The teeth preserve at their lateral edges a contact surface for other premaxillary teeth. Among the Anostomidae, premaxillary symphyseal teeth with a massive unicuspid incisiform shape are characteristics of *Megaleporinus*, of which eleven species are recognized (Ramirez et al. 2017; Birindelli et al. 2020). In contrast, *Abramites*, *Anostomoides*, and *Leporinus* have compressed teeth with a mesial ridge and usually a single outstanding cusp, often accompanied by smaller lateral cusps in juveniles (see Birindelli et al. 2013; Assega and Birindelli 2019). Our comparisons suggest also that the specimens AMU-CURS-851 resemble the premaxillary symphyseal teeth of *Megaleporinus muyscorum* in size and morphology (Fig. 5C1, C2), the only trans-Andean species inhabiting the Magdalena River basin. However, given that comparisons with *Megaleporinu*s species were not exhaustive, we prefer to keep the specimens from San Gregorio Formation in open nomenclature. These specimens from the NCC locality represent the first fossil record for this genus.

*Schizodon* Agassiz, in Spix and Agassiz, 1829

*Schizodon corti* Schultz, 1944

*Schizodon* cf. *S. corti*

(Fig. 5D1–O).

*Locality*: NCC (conglomerate, Fig. 3B).

*Material*: Seventy teeth from the premaxilla and dentary (AMU-CURS-849–850 and -1141).

*General description, comparisons and remarks*: Teeth are up to 3.6 mm in height and 1.5 mm wide. The premaxillary teeth (Fig. 5D1–I) are essentially straight, possessing three cusps, with a concave and convex shape in lingual and labial sides, respectively. Dentary teeth (Fig. 5J–O) are recurved and chisel shaped, with a concave and convex shape in lingual and labial sides, respectively. Symphyseal teeth bearing three cusps, and between two and three cusps in the second and third positions. Myers (1950) used dental morphology to diagnose most genera of the Anostomidae, as it was widely used method then (Winterbottom 1980; Garavello and Santos 2009; Ramirez et al. 2017). The genera *Anostomus*, *Pseudanos*, and *Petulanos* have compressed teeth (i.e., without mesial ridge) with three or four rounded cusps of similar size on the premaxilla and dentary (see Myers 1950; Winterbottom 1980). *Gnathodolus*, *Sartor*, and *Synaptolaemus* have compressed teeth with three weak cusps on the premaxilla, and four, three, or one (respectively) extremely elongated teeth on the dentary (Myers 1950). *Laemolyta* has compressed teeth with three to five rounded cusps of similar size on the premaxillary (similar to *Anostomus*, for example), and compressed teeth with a truncated cutting edge on the dentary (see Mautari and Menezes 2006). *Rhytiodus* and *Schizodon* also have compressed teeth, but with acute three to five cusps on the premaxilla and dentary. The other genera have teeth with a mesial ridge and usually a single outstanding cusp. Based on these comparisons, the fossil teeth described from the NCC locality are consistent with *Schizodon*. According to van der Sleen and Albert (2018), *Schizodon* is represented by at least 14 species in the cis-Andean basins, and the knowledge of their intraspecific dental variation is poorly known (Sidlauskas and Vari 2008). The only trans-Andean species is *Schizodon corti* (Fig. 5P–S), which inhabits the Lake Maracaibo basin (Rodríguez-Olarte et al. 2009). Given the locality of the fossil teeth, we herein tentatively identify them as belonging to cf. *S*. *corti*. Nevertheless, better comparisons are due once more comparative material is available. These specimens from the NCC locality represent the first fossil record for the genus *Schizodon*.

Erythrinidae Valenciennes, 1847

*Hoplias* Gill, 1903

*Hoplias* sp.

(Fig. 6A–F).

*Locality*: NCC (conglomerate, Fig. 3B).

*Material*: Two hundred and forty-eight isolated teeth of indeterminate jaw position (AMU-CURS-824, -860, -1142 and -1215).

*General description, comparisons and remarks*: Teeth ranging up to 5.5 mm in height. These teeth are characteristics of homodont dentition, straight, or slightly curved with a conical and pointed shape. Crowns of pyramidal shape characterize the uppermost part of the teeth with distinctly sharp edges, and the basal portion is wider with a parallel sulcus. Half way up the crown, the tooth shows a narrowing or “waist” that separates the top of the crown from the base. The morphology of the specimens from the NCC locality is indistinguishable from that of the extant representatives of *Hoplias* (Fig. 6G1–I). Specific determinations are not possible with only isolated teeth.

Serrasalmidae Bleeker, 1859 (sensu Van Der Laan, 2018)

*Mylossoma* Eigenmann and Kennedy, 1903

*Mylossoma* sp.

(Fig. 6J1–N2).

*Locality*: NCC (conglomerate, Fig. 3B).

*Material*: Five complete symphyseal dentary teeth (AMU-CURS-1216).

*General description, comparisons and remarks*: The symphyseal teeth are up to 2.7 mm in height and up to 2 mm in width (Fig. 6J1–N2). The teeth are labiolingually and mesiodistally expanded, and have an oval base and smooth surfaces. In occlusal view, these are characterized by an ovoidal groove bordered by a labial transverse peaked crest and a lower lingual ridge. Other *Mylossoma* species within the “pacu clade” (see Thompson et al. 2014), such as *Colossoma* and *Piaractus*, also have a combination of molariform-like teeth adapted for crushing hard foods. Our comparative results suggest that teeth in adults are much smaller in *Mylossoma* than in *Colossoma* and *Piaractus*. The premaxillary and dentary teeth of the above-mentioned genera look very similar, especially those of *Colossoma* and *Piaractus,* which seem to be indistinguishable, hampering taxonomic identifications with isolated teeth. However, as it has been noticed by Dahdul (2004), and supported by our comparisons, fossil and recent symphyseal mandibular teeth, in both juveniles and adults of *Mylossoma*, *Colossoma*, and *Piaractus*, have a diagnostic concavity in the lingual face, in which the elevation of the distal edge is different among these genera. The *Mylossoma* specimens from the NCC locality differ from those of *Colossoma* and *Piaractus* and are practically indistinguishable from those of the extant species of *Mylossoma* (Fig. 6O1–P2), in which the symphyseal specimens are characterized by a narrow and low distal edge. Given that the morphology of AMU-CURS-1216 specimens is similar to both that of *Mylossoma acanthogaster*, the only trans-Andean species inhabiting the Lake Maracaibo basin (Rodríguez-Olarte et al. 2009) as well as to the other four recognized Cis-Andean species (Mateussi et al. 2018), more accurate specific identification for these specimens is not possible.

Serrasalmidae indet.

? “pacu clade.”

(Figs. 6Q and 7A1–H).

*Locality*: NCC (conglomerate, Fig. 3B).

*Material*: Forty-two dentaries and premaxillary isolated teeth represent the sample (AMU-CURS-858–859 and -1143).

*General description, comparisons and remarks*: Teeth are up to 2.5 mm in height and 2 mm in width, although some broken and incomplete specimens could be larger. A molariform-like shape, being labiolingually and mesiodistally expanded with an oval base and a high crest, characterizes the teeth from the outer row (Fig. 7A1–D2). A molariform-like and elongated shape with a transverse and strongly peaked crest that is slightly curved to the lingual side, characterizes teeth from the inner row (Fig. 7E1–H). In all specimens, the transverse crest lacks serration. As previously mentioned, non-symphyseal mandibular teeth of the extant “pacu clade” (*Mylossoma*, *Colossoma* and *Piaractus*) look similar in shape across species. The specimens AMU-CURS-858–859 and AMU-CURS-1143 are comparable in morphology and size with the teeth of *Mylossoma*, the only representative of the "pacu clade" and Serrasalmidae so far registered for the Vergel Member. However, in our taxonomic comparisons we have been able to notice that non-symphyseal mandibular teeth of both *Colossoma* and *Piaractus* juveniles are comparable in size and morphology with those of *Mylossoma*. Added to this, other species of Serrasalmidae, especially some included in the “*Myleus* clade” (see Thompson et al. 2014), also have molariform-like teeth adapted for crushing hard foods (van der Sleen and Albert 2018). Thus, we neither allocate these isolated teeth to generic level nor discard that they could belong to more than one taxon within the “pacu” or “*Myleus*” clades.

Cichliformes Betancourt-R et al., 2013

Cichlidae Bonaparte, 1835

Cichlidae indet.

(Fig. 7I1–M2).

*Locality*: NCC (conglomerate, Fig. 3B).

*Material*: Ten complete and fragmented dorsal and anal fin spines (AMU-CURS-1223–1224).

*General description, comparisons and remarks*: The only complete spine is 21 mm in length (Fig. 7J1–J3). The spines are robust and elongated, with a sharp end at the apical section. The anterior edge is smooth, the posterior one is characterized by a deep median grove, and the lateral sides are ornamented by parallel groves. A median foramen, lateral condyles, and posterior condylar process characterize the articular section of the spine. The specimens AMU-CURS-1223 have the typical morphology observed in dorsal and anal spines of cichlids. Nevertheless, it is difficult to make precise taxonomic identifications based on isolated spines. For this reason, these spines from Vergel Member cannot be referred to beyond indeterminate cichlids.

Siluriformes (sensu Grande, 1987)

Ariidae Bleeker, 1862

*Sciades* Müller and Troschel, 1849

cf. *Sciades* sp.

(Fig. 8A1–A3).

*Locality*: NCC (conglomerate, Fig. 3B).

*Material*: A fragmented dorsal spine (AMU-CURS-1058).

*General description, comparisons and remarks*: The spine is 22 mm in length, with only the anterior portion preserved. Most of the articular region is missing, preserving only part of the left lateral wing. The spine body is robust with a triangular shape in cross section (Fig. 8A1–A3) with lateral sides characterized by a striated ornamentation. A crest with thick tubercles and a posterior side with a deep groove characterize the body of the spine. The specimen AMU-CURS-1058 is closer in morphology to the dorsal spines of the extant species of *Sciades* (Fig. 8B1, B2) than to any other species of marine or freshwater catfish that we have been able to compare.

Callichthyidae Bonaparte, 1838

Callichthyidae indet.

(Fig. 8C1–G2).

*Locality*: NCC (conglomerate, Fig. 3B).

*Material*: Four pectoral-fin spine fragments, one left (AMU-CURS-1167a) and three of indeterminate position (AMU-CURS-1167b-1668), plus two bony plate fragments of the body armor (AMU-CURS-1169).

*General description, comparisons and remarks*: The two proximal pectoral-fin spines AMU-CURS-1167 are 11 and 13 mm in length, respectively; in both specimens, the articular process is missing. The shaft is ovoid in section, with the anterior and anterodorsal edges ornamented by small circular odontodes bases (Fig. 8C1, C2). The posterior edge preserves a strong dentation. Specimens AMU-CURS-1668 are ovoid in section and anterior and anterodorsally ornamented by small odontode bases and a well-developed dentation in the posterior edge (Fig. 8D1–E). Dorsal-fin spines anteriorly and anterodorsally ornamented by small odontodes and well-developed posterior dentitions are typical of Callichthyidae (Lundberg 1997). Although the bony plate fragments are incomplete (Fig. 8F–G2), an elongated shape with smooth surface can be observed, a feature that characterizes the body armor plates of the Callichthyidae taxa (e.g., van der Sleen and Albert 2018). Due to the absence of diagnostic characters in the specimens, the presence of more than a single taxon cannot be ruled out.

Doradidae Bleeker, 1858

Astrodoradinae Higuchi et al., 2007

*Amblydoras* Bleeker, 1862

cf. *Amblydoras* sp.

(Fig. 8H1–H3).

*Locality*: NCC (conglomerate, Fig. 3B).

*Material*: A partial left cleithrum (AMU-CURS-1225), including the nearly complete posterior cleithral process and its base.

*General description, comparisons and remarks*: The cleithrum is 14 mm in length and 6 mm in maximum height. The anterior portion, corresponding to the base of the posterior cleithral process, including part of the cleithrum bulge laterally, and part of the sulcus medially, where the dorsal articular process of the pectoral-fin spine inserts. Dorsal process of cleithrum partially preserved, immediately dorsal to the anterior portion of posterior process of cleithrum. Medial face of posterior process of cleithrum smooth and concave at base. Lateral face of posterior cleithral process concave at base and straight posteriorly, bearing a longitudinal series of aligned spines and a longitudinal keel immediately dorsal to the series of spines (Fig. 8H1–H3). Series of spines, including six small protuberances near base, from anteriormost portion to terminus of dorsal process, and posteriorly to that point, possessing six larger protuberances well spaced. Protuberances from 0.01 to 0.05 mm in height. Tip of posterior cleithral process straight and blunt. The presence of a series of well-spaced aligned spines in AMU-CURS-1225 distinguishes this fossil from most species of Doradidae, as this is a characteristic feature of Astrodoradinae (Higuchi et al. 2007; Birindelli 2014). Among Astrodoradinae, only *Amblydoras*, *Anadoras*, and *Astrodoras* possess relatively large, distinct well-spaced spines that are aligned on the posterior cleithral process. Of the three aforementioned genera, *Anadoras* and *Astrodoras* have the posterior cleithral process slightly deeper and more triangular than *Amblydoras*. Therefore, the AMU-CURS-1225 is most similar to *Amblydoras* (Fig. 8I1, I2). The specimen AMU-CURS-1225 from the Vergel Member represents the first fossil record for the genus *Amblydoras*.

*Scorpiodoras* Eigenmann, 1925

cf. *Scorpiodoras* sp.

(Fig. 8J1–J3).

*Locality*: NCC (conglomerate, Fig. 3B).

*Material*: A partial right cleithrum (AMU-CURS-1226), including exclusively the posterior half (or third) of the posterior cleithral process.

*General description, comparisons and remarks*: AMU-CURS-1226 is approximately triangular in shape, with around 9 mm in length and 3 mm in maximum height. Anterior border convex with irregular margin, dorsal margin is concave and the ventral one straight. Posterior tip of process blunt. Medial face smooth. Lateral face ornamented with more or less six longitudinal ridges. Two dorsal most ridges, more or less continuous (i.e., not denticulated) and relatively short (approximately 0.01 mm of height). Next two longitudinal ridges (0.01 mm of height), from dorsal to ventral margins, denticulated and converging approximately at middle of the specimen. Fifth ridge (from dorsal to ventral margins) largest, approximately of 0.2 to 0.8 mm in height, more robust near tip of process and distinctly denticulated (or composed of coalescent distally oriented spines). Tip of posterior cleithral process somewhat tilted laterally. The presence of strong ridges (Fig. 8J2, J3), including a horizontal series of spines (even as denticulated ridges), distinguishes this fossil from most species of Doradidae, as this is a characteristic feature of Astrodoradinae (Higuchi et al. 2007; Birindelli 2014). The presence of denticulated longitudinal series and the distally curved posterior cleithral process is only present in *Scorpiodoras* (Fig. 8K1, K2). The specimen AMU-CURS-1226 assigned to cf. *Scorpiodoras* sp. from the NCC locality represents the first fossil for the genus.

Doradidae indet.

(Figs. 9A1–J and 10A1–I2).

*Locality*: NCC (conglomerate, Fig. 3B).

*Material*: An assortment of doradid remains, including three skull fragments (AMU-CURS-1170, -1227 and -1175), 62 pectoral (AMU-CURS-667, -1233–1234) and 11 dorsal spines (AMU-CURS-1180), and four mid-lateral scutes (AMU-CURS-864 and AMU-CURS-1232a-c).

*General description, comparisons and remarks*: AMU-CURS-1227 is a complete disarticulated parieto-supraoccipital (Fig. 9A1, A2). It is around 12 mm in length, 8 mm in width, and 3 mm in height. Nonagon shaped, with posterior margin straight (presumably sutured to anterior nuchal plate). Anterior margin pointed with contralateral concave margins (presumably sutures to frontals). Lateral margin composed of three stretches of concave margins, with width greater around last third of bone. Dorsal surface relatively flat and ornamented with small depressions, including two relatively parallel lines of depressions near the anterolateral tips (close to presumably suture between frontals and sphenotic). Ventral surface of bone with three concavities, one large covering most of the surface and two contralateral ones in the posterior portion, divided by a longitudinal bony septum. The truncated posterior margin in AMU-CURS-1227 leaves no doubt that the specimen belongs to either a Doradidae or an Auchenipteridae specimen. The relative flatness and the proportions (i.e., more elongated than wide) are more similar to features of Doradidae than to Auchenipteridae.

AMU-CURS-1170 is a partial parieto-supraoccipital, including possibly the middle of the bone (Fig. 9B). Specimen with approximately 9 mm in maximum width and 7 mm in length. Dorsal surface flat with some ornamentation composed of small depressions, including a part of the longitudinal line of depression linked to the sensory canal that runs from the parieto-supraoccipital to the frontals, and the transversal line of relatively larger depressions that runs in the middle of the parieto-supraoccipital. The flat parieto-supraoccipital bone is ornamented with small depressions, resembling the parieto-supraoccipital of Doradidae. It is likely that AMU-CURS-1227 and AMU-CURS-1170 belong to cf. *Amblydoras* sp. or cf. *Scorpiodoras* sp.; however, given the broken nature of the specimens and the small portion of diagnostic anatomy they preserve, we suggest an allocation at the family level.

AMU-CURS-1175 is a partial left cleithrum fragment, of around 7 mm in length and 7 mm in maximum height (Fig. 9C). Although the poor preservation of this specimen precludes a more precise taxonomic identification, its general morphology is somewhat different from that of the cleithrum of *Amblydoras* and *Scorpiodoras*. This suggests the presence of a third Doradidae taxon in the Vergel Member assemblage.

The pectoral-fin spines (AMU-CURS-667, -1233–1234) include fourteen right specimens, 16 left, and 30 of indeterminate position. The spines are broken (Fig. 9E1–J), and the most complete one is 29 mm in length (Fig. 9D1–D3). The shaft is robust, well ossified, and flattened dorso-ventrally, with an oval shape in cross section. Both dorsal and ventral surfaces of the shaft bear coarse parallel groves. The anterior edge is characterized by small triangular and sharp denticles, which are inclined in the distal direction. Denticles of the posterior edge are bigger than the ones on the anterior edge, and these are inclined toward the proximal direction of the spine. In the well-preserved articular sections, the dorsal articular process is semi-circular and wide, the articular groove is triangular and large, and the anterior and ventral processes are well developed. Dorsal-fin spines (AMU-CURS-1180) are also in fragmentary condition, preserving only the proximal-half portions (Fig. 10A1–D2); the most complete one is 13 mm in length. These are robust and triangular in cross section, with lateral sides characterized by coarse parallel groves. In most specimens, the dorsal section is smooth, but two of the specimens bear triangular denticles inclined distally. The articular region is triangular with a prominent anterior process, a circular articular foramen, and a well-developed medial process. These fossil pectoral-fin and dorsal-fin spines resemble those of the extant species of *Amblydoras*, *Scorpiodoras*, *Anadoras* (e.g., Figs. 9K1–L3 and 10E1–F3), and other members of Astrodoradinae. However, due to the fragmentary and poor preservation of most of the specimens, added to the poor knowledge of intraspecific spine variation in Doradidae catfishes, determinations that are more accurate are not possible.

The partial mid-lateral scute (AMU-CURS-864a) is 1.5 mm in length (thorn length), preserving the entire thorn and its base (Fig. 10G). The thorn is strongly curved and posteriorly oriented. AMU-CURS-864b is also a partial mid-lateral scute of approximately 0.5 mm in length, preserving only part of the thorn near its base (Fig. 10H). The thorn is curved, posteriorly oriented, with a relatively small base (likely smaller than half of thorn). AMU-CURS-1232a-b correspond to two mid-lateral scutes. One of the specimens (AMU-CURS-1232a) is partially preserved (right body side), with length approximately 1.2 mm and height around 0.6 mm (Fig. 10I1, I2). AMU-CURS-1232b is a mid-lateral scute thorn completely preserved, posteriorly oriented, with distal margin convex, base larger than half of thorn (measured from anterior insertion of base to posterior tip). Doradidae are the only catfishes bearing mid-lateral scutes with a posteriorly oriented thorn. However, due to the fragmentary nature of the specimens, an identification to generic level is impossible. In any case, the mid-lateral scutes of the caudal peduncle usually possess elongated thorns, whereas the mid-lateral scutes on the center of the body usually possess smaller thorns with relatively large bases.

Heptapteridae Gill, 1861

*Pimelodella* Eigenmann and Eigenmann, 1888

cf. *Pimelodella* sp.

(Fig. 10J).

*Locality*: NCC (conglomerate, Fig. 3B).

*Material*: One incomplete pectoral-fin spine of indeterminate position, 6.5 mm in length (AMU-CURS-1183).

*General description, comparisons and remarks*: The specimen corresponds to the distal part of the spine, with a well-ossified and compressed shaft of sub-rectangular shape in cross section. Both dorsal and ventral surfaces of the shaft exhibit parallel shallow grooves and small pits. The anterior edge of the spine is characterized by small triangular denticles inclined toward distal direction where they become progressively smaller. The most distal section of the anterior edge (apical section) lacks denticles. In contrast, denticles in the posterior edge tend to be lightly inclined toward the proximal direction of the spine, and are wider, larger, and better defined than the denticles of the anterior edge. Although in AMU-CURS-1183 the articular section is missing, the combination of the above-mentioned characters supports the specimen’s assignment within Heptapteridae. AMU-CURS-1183 is tentatively assigned to cf. *Pimelodella*, whose pectoral spines in fossil and extant species are characterized by an ornamentation pattern that can be clearly differentiated from other Siluriformes genera (Bisbal and Gómez 1986; Lundberg and McDade 1986; Bogan et al. 2020).

Loricariidae Rafinesque, 1815

Hypostominae Kner, 1853

Hypostominae indet.

(Fig. 10K1–P).

*Locality*: NCC (conglomerate, Fig. 3B).

*Material*: Nine pectoral-fin spine fragments (AMU-CURS-817 and -1173), and three isolated odontodes (AMU-CURS-876).

*General description, comparisons and remarks*: Pectoral-fin spine fragments of up to 15 mm in length (Fig. 10K1–N2). The three isolated and elongated odontodes are up to 3 mm in length (Fig. 10O, P). In the pectoral-fin spines, the articular region is missing, and only one fragmentary anterior section is preserved. The shaft of the spines is robust, slightly flattened dorso-ventrally with an oval cross section; only the two largest specimens preserve the articular groove, which is elongated in outline. Small circular odontode bases ornament the anterior and anterodorsal section. Parallel low ridges characterize the dorsal surface, forming grooves with small circular odontode bases; a well-developed line of circular odontode bases is present along the posteriodorsal edge. In the posterior margin of the shaft, a longitudinal sulcus is present. Pectoral spines bearing well-developed odontodes could be a representative character of Hypostominae, contrary to other loricariids subfamilies where the pectoral spine is always with thick skin or dermal plates (e.g., van der Sleen and Albert 2018). AMU-CURS-1173 resembles the shaft of *Hemiancistrus*; nevertheless, the fragmentary condition limits further recognition of more than one taxon and the taxonomic assignment beyond Hypostominae.

Loricariidae indet.

(Fig. 10Q, R).

*Locality*: NCC (conglomerate, Fig. 3B).

*Material*: Four body plates of the body armor (AMU-CURS-1230) and five broken and eroded small articulate sections of pectoral spines (AMU-CURS-1231).

*General description, comparisons and remarks*: Three of the body armor plates are fragmented and of indeterminate position; the complete specimen is 2.7 mm wide (Fig. 10Q) and presumably corresponds to a plate of the median position. Both the complete and the fragmented plates are ornamented by parallel rows of odontodes forming keels. Loricariids belong to a diverse group of armored Siluriformes (van der Sleen and Albert 2018), and taxonomic identification based on their isolated body plates is a difficult task. The fragmentary condition of the specimens limits further taxonomical recognition, especially to differentiate whether these materials also belong to the above-mentioned Hypostominae loricariids or not.

Pimelodidae (sensu Lundberg and Littman, 2003)

*Platysilurus* Haseman, 1911

cf. *Platysilurus* sp.

(Fig. 11A1–B3).

*Locality*: NCC (conglomerate, Fig. 3B).

*Material*: Three incomplete left pectoral spines (AMU-CURS-1172).

*General description, comparisons and remarks*: The specimens are up to 14 mm in length, preserving part of the shaft and the articular region (Fig. 11A1–B3); these are slightly curved, robust, and flattened dorso-ventrally with an oval cross section. Most of the spines preserve the dorsal articular process, which is robust and somewhat rectangular in shape; the anterior process is preserved only in two of the specimens, and the ventral process is missing. The articular groove is triangular in outline. Both dorsal and ventral surfaces of the shaft bear coarse subparallel grooves, and the anterior and posterior edges have small triangular denticulations. AMU-CURS-1172 are morphologically more similar to the dorsal and pectoral spines of the two extant species of *Platysilurus* (Fig. 11C1–C3) (for species diversity see van der Sleen and Albert 2018) than any other species of marine or freshwater catfish that we have been able to compare. However, due the poor preservation of the spines, we tentatively assign AMU-CURS-1172 specimens to cf. *Platysilurus*.

Pimelodidae indet.

(Fig. 11D1–E3).

*Locality*: NCC (conglomerate, Fig. 3B).

*Material*: Two incomplete right and left pectoral spines (AMU-CURS-1228).

*General description, comparisons and remarks*: The most complete spine reaches 25 mm in length (Fig. 11E1–E3). In both spines, the articular region is present. However, the dorsal process is eroded and broken in one of the specimens; anterior and ventral processes are missing. The shaft is flattened and slight curved, with parallel and longitudinal grooves. The anterior edge is characterized by small denticles inclined toward distal direction; in contrast, the posterior edge has bigger triangular denticles inclined toward the proximal direction of the spine. AMU-CURS-1228 resembles the pectoral spines of extant and fossil species of *Pimelodus* (see Lundberg 1997; Vallone et al. 2017) more than any other pimeloid species that we could compare. However, due to the fragmentary condition of AMU-CURS-1228, we prefer to tentatively assign them to Pimelodidae indet. Clear morphological differences between the spines AMU-CURS-1172 assigned to cf. *Platysilurus* (Fig. 11A1–B3) and AMU-CURS-1228 unequivocally support the presence of at least two pimeloids in the NCC assemblage.

Siluriformes indet.

(Fig. 11F–M).

*Locality*: NCC (conglomerate, Fig. 3B).

*Material*: Ninety-nine cranial and postcranial isolated elements, most of them in eroded and fragmentary condition that does not permit a confident identification beyond Siluriformes indet.

*General description, comparisons and remarks*: The sample includes a fragment of a left post-temporal and two other skull fragments of indeterminate position (AMU-CURS-1175, Fig. 11G, H), a precaudal vertebrae with a centrum of 10 mm wide and 8.3 mm high (AMU-CURS-1174, Fig. 11I1, I2), and 95 pectoral and dorsal spine fragments (AMU-CURS-867, and -1180–1183, Fig. 11J–M). Although shaft fragments represent most of the dorsal and pectoral spines, some eroded articular regions have also been identified in the sample (Fig. 11K). These dorsal and pectoral catfish spines are in a bad fragmentary state with a marked degree of erosion preventing the recognition of diagnostic elements that could allow taxonomic identification even at the family level. The specimen AMU-CURS-1237 is a partial left cleithrum with around 8.5 mm in length (Fig. 11F) whose general morphology and ornamentation suggests clear differences with cleithrum bones of the extant specimens of Ariidae, Callichthyidae, Doradidae, Heptapteridae, and Loricariidae taxa described above from the NCC assemblage (Table 1). AMU-CURS-1237 could belong to another taxon, probably to a pimeloid catfish. However, future new fossil specimens would be necessary to clarify its taxonomy.

Synbranchiformes (sensu Gosline, 1983)

Synbranchidae Swainson, 1838

*Synbranchus* Bloch, 1795

*Synbranchus* sp.

(Fig. 12A1–J).

*Locality*: NCC (conglomerate, Fig. 3B).

*Material*: One right and two left fragmented dentaries (AMU-CURS-1146-a-c), a pharyngeal bone (AMU-CURS-1146-d), and 15 isolated vertebrae (AMU-CURS-1171).

*General description, comparisons and remarks*: The fragmented dentaries (Fig. 12A1–C3) are between 2 and 3.5 mm long, preserving only their anterior-symphyseal section. The anterior margin of the symphyseal region is rounded, exhibits a well-developed process, and the dorsal margin is covered by high subcircular tooth implantations. On the external face of each dentary, next to the symphyseal region, two well-developed foramina are observed. The pharyngeal bone (Fig. 12D1, D2) is 3 mm long, triangular, and covered by subcircular tooth implantations.

Most of the vertebrae are eroded and incomplete (Fig. 12G1–J), corresponding to six precaudal, three caudal, and one of indeterminate position. Precaudal vertebrae are characterized by a central body with the anterior face practically flat or slightly concave, and the posterior face wider than the anterior with a deep conical cavity; the transverse process is wide and ventrolaterally projected. Caudal vertebrate are not well preserved. However, like the anterior ones, a flat or slightly concave face characterizes the central body; the anterior face is bigger than the anterior, with a deep conical cavity, features that are characteristics in the vertebrate of Synbranchidae (Bogan et al. 2012). The fossil dentaries AMU-CURS-1146-a-c resemble those of the extant *Synbranchus marmoratus* (Fig. 12E1–F2), and their size suggests that these fossils would be fragments of small-sized individuals. Due to the poor preservation of the fossil dentaries, the lack of diagnostic elements in the isolated vertebrae, as well as the scarce osteological comparative material for some of the recognized living species of *Synbranchus*, especially those from the Amazon basin (see Utsunomia et al. 2014), a more accurate specific determination is not possible. The *Synbranchus* specimens from the NCC locality represent the oldest fossil record for this genus, since its fossil record was restricted to the Late Pleistocene of Argentina (Bogan et al. 2012).

Actinopterygii indet.

(Fig. 13A–Q).

*Locality*: NCC (conglomerate, Fig. 3B).

*Material*: Abundant isolated elements that include seven pharyngeal teeth (AMU-CURS-1145) and 12 other teeth of indeterminate position (AMU-CURS-1178). One hundred twenty-four complete and fragmented vertebrae of the precaudal and caudal regions (AMU-CURS-1176–1177 and -1240–1241). Forty-two fragmented cranial and postcranial bones (AMU-CURS-1179 and -1238) and 10 scale fragments (AMU-CURS-1863).

*General description, comparisons and remarks*: The isolated teeth are up to 2 mm in height (Fig. 13A–D). The poorly preserved cranial and postcranial bones (Fig. 13E, F) and scales (Fig. 13N–Q), lack diagnostic elements that allow a more detailed taxonomic assignment. In the case of the vertebrae, only a few specimens are complete (e.g., Fig. 13L1, L2). The rest of the specimens are incomplete and in a very poor preservational state, particularly in most of the vertebral centra. The largest vertebra in the sample does not exceed 4.5 mm in length (Fig. 13G–M). Given the small size of the vertebrae, they could belong to juveniles or other small-sized species, different from the taxa that can be recognized for the NCC assemblage (Table 1). Due to the poor preservation of the vertebrae and the scarcity of recent comparative material, a more detailed taxonomic identification is not possible at this time.

Lissamphibia Haeckel, 1866

Anura Fischer von Waldheim, 1813

Pipidae Gray, 1825a

*Pipa* Laurenti, 1768

cf. *Pipa* sp.

(Fig. 14A1, A2).

*Locality*: NCC (conglomerate, Fig. 3B).

*Material*: An incomplete sacral vertebra fused to a partial urostyle (AMU-CURS-722).

*General description, comparisons and remarks*: AMU-CURS-722 (total length 3.7 mm) preserves the vertebral centrum with a portion of the urostyle and poorly preserved transverse processes and prezygapophyses (a significant remnant of the right prezygapophyseal facet but much less of the left). Although the compound element is incomplete and moderately damaged, it clearly shows a single anterior condyle, planar diapophyses, a sacral sagittal dorsal ridge, and broad spinal foramina (one per side), which are useful characters for referring AMU-CURS-722 to pipids such as cf. *Pipa* sp. (see Delfino and Sánchez-Villagra 2018 and references therein). The specimen AMU-CURS-722 must have belonged to an individual of small size and the absence of a ridge on the sacral transverse process (there is only a hint of a very weak, elongated convexity directed posterolaterally) resembles characters present in *Pipa parva* (Fig. 14B1, B2) which is currently present in Falcón State (Mijares-Urrutia and Arends 2000). However, the poorly preserved condition of the specimens precludes a taxonomic identification beyond generic level.

Anura indet.

(Fig. 14C1–L).

*Locality*: NCC (conglomerate, Fig. 3B).

*Material*: The specimens correspond to 20 isolated and fragmented cranial and postcranial microelements that, due to their poorly preserved condition and lack of diagnostic characters, cannot be confidently identified beyond Anura indet.

*General description, comparisons and remarks*: Cranial elements include maxillary fragments up to 2.7 mm in length (AMU-CURS-723, -1149, -1162, and -1165–1166) preserving some dental positions, but not complete teeth (Fig. 14C1–F2). Postcranial elements include a distal fragment of a ?right ilium of 2.19 mm in length (AMU-CURS-1150, Fig. 14G), a distal portion of left humerus of 1.9 mm in length (AMU-CURS-1152, Fig. 14H), distal fragment of a right radioulna with a length of 4.2 mm (AMU-CURS-1151, Fig. 14J), and some fragmented vertebrae and other indeterminate limb bones (AMU-CURS-807 and -1153, Fig. 14I, K, L).

Testudines Batsch, 1788 [Joyce et al., 2020a].

Cryptodira Cope, 1868 [Joyce et al., 2020c]

Testudinidae Gray, 1825a, b [Joyce et al., 2021]

*Chelonoidis* Fitzinger, 1835

*Chelonoidis* sp.

(Fig. 15A1–A3).

*Locality*: NCC (conglomerate, Fig. 3B).

*Material*: An incomplete right femur (AMU-CURS-584).

*General description, comparisons and remarks*: AMU-CURS-584 has a length of 70 mm, preserving the shaft, part of the proximal metaphysis and the distal epiphysis. Although the distal articular surface of the specimen is not perfectly preserved, the articular facets are clearly visible. Morphological features of specimen AMU-CURS-584 coincide with those observed in femora of extinct (e.g., Turvey et al. 2017) and extant *Chelonoidis* (e.g., *Chelonoidis carbonarius* and *Chelonoidis denticulatus*).

Pleurodira Cope, 1865 [Joyce et al., 2020b]

Chelidae Lindholm, 1929 [Joyce et al., 2021]

*Chelus* Duméril, 1806

*Chelus* sp.

(Fig. 15B1, B2).

*Locality*: NCC (conglomerate, Fig. 3B).

*Material*: The specimen corresponds to a plastron fragment (AMU-CURS-839).

*General description, comparisons and remarks*: AMU-CURS-839 is 36 mm in length. It constitutes the anterolateral portion of a left xiphiplastron, exhibiting on its ventral surface a densely vermiculated bone surface, and preserving the beginning of the thick xiphiplastron tip characteristics of *Chelus*. On the dorsal surface (Fig. 15B1), a portion of the pubis is preserved.

Podocnemididae Cope, 1868 [Joyce et al., 2021]

Podocnemididae indet.

(Fig. 15C1–L2).

*Locality*: NCC (conglomerate, Fig. 3B) and SGOP (conglomerate Ly1, Fig. 3C).

*Material*: A total of 51 postcranial remains: 48 from NCC (AMU-CURS-79, -547, -555, -560, -567, -571–572, -579–580, -675, -763, -838, and -866) and three from SGOP (AMU-CURS-1305–1307) localities.

*General description, comparisons and remarks*: The specimen AMU-CURS-571 constitutes a cervical vertebra (Fig. 15C1–C5), resembling in length, height, and morphology cervical 3 of the extant *Podocnemis expansa* (AMNH 62947). The prezygapophyses are projected dorsally with rounded tips. The postzygapophyses are laterally projected, exhibiting a facet for the articulation with cervical 4. The posterior condyle has a horse-saddle shape, which is the most typical condition of cervicals 3 to 7 in podocnemidids. The ventral portion of the centrum lacks a keel and forms a slightly concave margin. AMU-CURS-675 is a nearly complete left ilium, preserving part of the concave acetabulum (Fig. 15D), and AMU-CURS-1305 constitutes a nearly complete left ischium, missing some portions of its most ventrodistal edge (Fig. 15E1, E2). Dorsoproximally, the latter exhibits the sutural surface that articulates with the pubis and ilium, as well as a smooth surface that makes part of the acetabulum capsule. In all its aspects, AMU-CURS-1305 resembles the left ischium of extant and fossil podocnemidids, as in *Podocnemis expansa* (AMNH-62947).

Carapace and plastron fragments (*n* = 48) are the most abundant podocnemidids remains in the NCC locality. AMU-CURS-547 corresponds to a lateral portion of a costal bone (Fig. 15I1, I2), potentially left costal 5, considering that it has the inguinal scar and on the ventral surface it lacks evidence of a sulcus between pleural scutes. It is attributed to podocnemidids, based on smoothly sculpted dorsal bone surface, and thinner thickness of the same, in contrast to representatives of the *Chelus* genus that also occur in the Vergel Member. AMU-CURS-555 corresponds to a lateral portion of a potential right costal 5, exhibiting a portion of the inguinal scar on its ventral surface. The specimen AMU-CURS-560 represents the medial portion of a costal bone, potentially left costal 3, exhibiting a smooth dorsal bone surface and marks of the sulci between pleural and vertebral scutes. AMU-CURS-567 is the medial portion of a costal bone, potentially right costal 8. On the dorsal surface, the sulcus between vertebral and pleural scutes is clearly defined, and on the ventral surface a portion of the iliac scar is preserved. AMU-CURS-572A is a neural bone, missing its anterior portion. Its dorsal surface is eroded and there is no clear evidence of a sulcus. AMU-CURS-866 is a neural 1, missing its anterior portion. On its dorsal surface the sulcus between vertebral scutes is visible. On the ventral surface, the scar for the attachment of the thoracic vertebra is preserved. AMU-CURS-572B represents a complete peripheral bone from the posterior margin of the carapace. On its dorsal surface, the sulci between marginal and pleural scutes are visible and well defined. AMU-CURS-579 corresponds to an isolated peripheral bone from the carapace–plastron bridge region (Fig. 15L1, L2); on its dorsal surface, the sulcus between marginal scutes is visible. The medial edge (sutural contact with the costal) has been affected by bioerosion creating pits between the serrated bone textures. AMU-CURS-1306 is a costal bone (Fig. 15J), potentially the right costal 6, due to the relatively straight medial margin and the sulci between vertebral and pleural scutes on its dorsal surface. In ventral view, the costal rib process is well defined. AMU-CURS-1307 corresponds to a peripheral bone from the posterior margin of the carapace (Fig. 15F). In dorsal view, the sulci between marginal and pleural scutes are well defined, as well as some annuli lines close to the boundary between marginal and the pleural.

AMU-CURS-838 corresponds to a nearly complete peripheral bone from the bridge region of the shell (Fig. 15G1, G2). On its dorsal surface, the sulci between marginals and pleural scutes are visible, as well as a dichotomic sculpturing bone surface, which can be the case of shells of some extant podocnemidids, for example, *Podocnemis lewyana*. AMU-CURS-79 represents the posterior tip of a right xiphiplastron (Fig. 15K1, K2); the dorsal surface of the ischial scar is preserved, indicating that both ischia met medially. The specimen AMU-CURS-580 is a partial right hypoplastron (Fig. 15H1, H2), missing part of its lateral region and most of the anteromedial region. On its ventral surface, the abdominofemoral sulcus is visible. Due to the absence of diagnostic characteristics defining possible morphotypes in the sample, we can only justify the presence of at least one podocnemidid taxon.

Testudines indet.

(Fig. 16A–E).

*Locality*: NCC (conglomerate, Fig. 3B).

*Material*: An assortment of 85 fragmentary and poorly preserved cranial and some postcranial bones and carapace/plastron remains (AMU-CURS-561, -569, -581, -693, -735, -840–844, -862, -883, -1059, -1122, -1154, -1160, -1185, and -1288–1289).

*General description, comparisons and remarks*: Due to their preservation, these remains lack diagnostic elements that allow a more confident assignment (e.g., Fig. 16D–E). Among the most representative elements that can be referred are a fragment of a small right premaxilla of 11.7 mm in length (AMU-CURS-1154, Fig. 16A), the distal section of a left fibula of 21 mm in length (AMU-CURS-1160, Fig. 16B1, B2), and a plastron fragment with a clear round bite mark (AMU-CURS-862, Fig. 16C1, C2).

Squamata Oppel, 1811b

Teiidae Gray, 1827

*Tupinambis* Daudin, 1802

*Tupinambis* s.l.

(Fig. 16F1–F3).

*Locality*: NCC (conglomerate, Fig. 3B).

*Material*: A fragmentary right dentary (AMU-CURS-721).

*General description, comparisons and remarks*: AMU-CURS-721 is 37 mm long and posteriorly and posteroventrally incomplete. In medial view (Fig. 16F3), the Meckel’s canal is entirely open (it reaches the posterior edge of the dentary symphysis) and broad. No teeth are preserved but they were clearly subpleurodont and surrounded by a porous tissue. A thick subdental shelf (3.7 mm) preserves the first 11 tooth positions, plus the anterior wall of the 12th position. The dental shelf is of uniform height up to the ninth tooth position, but thins slightly posteriorly. The remnants of the teeth indicate that their base was approximately cylindrical (but the last preserved tooth was slightly compressed labiolingually) and that their size decreased posteriorly up the fifth position and then increased up to the last preserved tooth that was clearly the largest of the preserved series (Fig. 16F1). Despite the presence of a matrix filling the concavities, it seems that medial to each tooth position there is a replacement socket, longer than it is wide. Along the posterior section of the dentary, a small tooth cusp is visible among the matrix filling the replacement socked of the 11th tooth position. The dentary symphysis reaches the level of the fifth alveolus. The outer surface of the dentary (Fig. 16F2) hosts six dental foramina aligned parallel to the straight dorsal edge of the element, and closer to it than to the ventral edge. Irregular postmortem traces are present on the outer surface.

The general morphology of AMU-CURS-721 matches that of large-sized teiid (Estes 1983; Nydam et al. 2007). It differs from that of *Dracaena* because of the higher number of tooth positions present in the symphysis and the higher number of small anterior teeth (actually tooth positions in AMU-CURS-721; Estes 1961). AMU-CURS-721 differs from extinct †*Paradracaena colombiana* (Estes 1961) (originally described from the middle Miocene of Colombia, Estes 1961, but also present in the Miocene of Brazil, Hsiou et al. 2009, and Peru, Pujos et al. 2009) in the nearly straight orientation of the dorsal edge of the dentary. Conversely, the morphology of AMU-CURS-721 is broadly congruent with that of *Salvator* and *Tupinambis*, whose comparative osteological diagnosis is still unknown (Hsiou et al. 2016) despite it having a relevant interest for paleontologists due to the rich fossil record of *Tupinambis*-like taxa (see Albino et al. 2006; Albino and Brizuela 2014). Waiting for a full description of the dentary of these two recently separated taxa (Harvey et al. 2012), AMU-CURS-721 is here referred to *Tupinambis* s.l. It is worth mentioning that *Tupinambis* is the only large-sized teiid currently inhabiting Falcón State (Mijares-Urrutia and Arends 2000).

(non-snake) Squamata indet.

(Fig. 16G–K).

*Locality*: NCC (conglomerate, Fig. 3B).

*Material*: Five cranial elements in a fragmentary condition (AMU-CURS-725, -797, -1148, and -1163–64).

*General description, comparisons and remarks*: The anterior section of a left maxilla (AMU-CURS-1148) is 1.5 mm in length and its distal section preserves the curved bone surface that forms part of the external narial opening (Fig. 16G). Only an incomplete recurved tooth is preserved, and on the lateral surface of the maxilla, a small foramen is visible. AMU-CURS-1164 is a right jaw fragment 2.8 mm in length preserving three complete teeth (Fig. 16H1, H2). AMU-CURS-1163 is a premaxilla (Fig. 16I) 2.9 mm in length preserving an incomplete tooth. The other two specimens are maxillary fragments (both specimens less than 1.5 mm in length) preserving one (AMU-CURS-797, Fig. 16K) and two (AMU-CURS-725, Fig. 16J) teeth, respectively. All the specimens have pleurodont dentition, and with the exception of AMU-CURS-1148, teeth are cylindrical and straight. In AMU-CURS-725, both teeth are robust, and one of these preserves a smaller accessory distal cusp. In the specimen AMU-CURS-1164, part of the subdental shelf is preserved with three closely spaced teeth, which are characterized by a triangular, pointed crown with sharp edges. Approximately, in the middle portion of the teeth, a clear narrow area delimits the transition between the pyramidal crown and the basal section; clear resorption pits are located at the base of the teeth. AMU-CURS-1163 apparently had a tooth with a flattened crown tip and the tooth in AMU-CURS-797 is slender, conical and well pointed (Fig. 16I). Strong differences in the morphology of premaxillary and maxillary teeth, and variation in tooth morphology along maxilla (or dentary) are usual in many lizard taxa. This might suggest that AMU-CURS-1148, AMU-CURS-1163, AMU-CURS-1164, and AMU-CURS-725 would not represent distinct taxa. In contrast, the tooth preserved in AMU-CURS-797 (Fig. 16K), which is small, slender, cylindrical, and well pointed, seems to belong to a different taxon than the above referred specimens. The specimens are not well preserved, and taking into account intraspecific variation, it is not possible to confidently state how many lizard taxa are present in the sample. Nevertheless, the subtle dental differences among AMU-CURS-725 and the other four specimens suggest the presence of at least two taxa.

Serpentes Linnaeus, 1758

Alethinophidia Nopcsa, 1923

Aniliidae Fitzinger, 1826 (sensu Vidal et al., 2009)

*Anilius* Oken, 1816

*Anilius scytale* (Linnaeus, 1758)

(Fig. 17A1–A5).

*Locality*: NCC (conglomerate, Fig. 3B).

*Material*: An incomplete trunk vertebra (AMU-CURS-1159).

*General description, comparisons and remarks*: AMU-CURS-1159 is missing part of the neural arch, small portions of the zygosphene and the right prezygapophysis. The vertebra is slightly wider than it is long (neural arch width: 5.0 mm; centrum length: 4.8 mm). The prezygapophyses are relatively long; they extend well anterolaterally in dorsal view and are dorsolaterally inclined in anterior view, reaching the level of the zygosphene. The neural arch is strongly depressed in posterior view. The zygosphene is relatively thin in anterior view. The cotyle is broader than it is high and is larger than the neural canal. The condyle is circular. The postzygapophyseal articular facets are large and ovoidal. The hemal keel is weakly expressed in its medial sector but well defined posteriorly, where it broadens slightly and is posteriorly directed (a groove on each side marks its posterolateral edge). The paradiapophyses are laterally eroded. The diapophysis is much smaller than the parapophysis; they are partially separated by a deep and well-defined groove that develops from their posterior edge in anterodorsal direction. There are no parapophyseal processes. On the ventral surface, medial to the parapophysis there is, on both sides, a sort of small bony bridge that connects the parapophysis to the cotyle; a foramen opens posteriorly to this bridge delimiting a channel that could be in connection with the deep groove that develops on the anterior surface between the parapophysis and the cotyle. The wide, much depressed, and almost flattened neural arch in posterior view, the elongated and much dorsally inclined prezygapophyses, the rather shallow posterior median notch of the neural arch, and the prominent interzygapophyseal constriction observable in AMU-CURS-1159, are characteristic features of the extant American pipe snake, *Anilius scytale* (Hoffstetter and Rage 1977; Rage 1984, 1998; Smith 2013; Head 2020), which is currently present in Venezuela, though not in Falcón State (Mijares-Urrutia and Arends 2000; Barrio-Amorós et al. 2002).

*Anilius* (and its sole species *A*. *scytale*) represents the sole extant taxon of Aniliidae, as recent studies have demonstrated that the extant Asian cylindrophiids, anomochilids, and uropeltids (which share several vertebral features in common) are only distantly related and should not be referred to this group (Gower et al. 2005; Vidal et al. 2009; Head 2020; Smith and Georgalis in press). Note that another potential aniliid genus was also present in the Neogene of the Amazonian region, i.e., †*Colombophis* Hoffstetter and Rage, 1977 (Hoffstetter and Rage 1977; Head et al. 2006; see also Hsiou et al. 2010 for a different taxonomic interpretation). The vertebra from the NCC locality further differs from *Colombophis* primarily by its much smaller size and less robust nature, and to a lesser degree by its more depressed neural arch, more slender and pointed prezygapophyses and postzygapophyses, and less thick zygosphene (Hoffstetter and Rage 1977; Head et al. 2006; Hsiou et al. 2010). We refer this vertebra to *A*. *scytale*, a taxonomic assignment supported also by geographic and stratigraphic rationale. The specimen AMU-CURS-1159 represents the first fossil occurrence of *Anilius scytale*.

Constrictores Oppel, 1811a (sensu Georgalis and Smith 2020).

Boidae Gray, 1825a

*Corallus* Daudin, 1803

*Corallus* sp.

(Fig. 17B1–B5).

*Locality*: NCC (conglomerate, Fig. 3B).

*Material*: An incomplete trunk vertebra (AMU-CURS-1157).

*General description, comparisons and remarks*: AMU-CURS-1157 is missing most of the left prezygapophysis and part of the right prezygapophysis, whereas part of the left side of the zygosphene is damaged. The vertebra is moderately large, with a centrum length of 7.4 mm and a neural arch width of 9.5 mm. The zygosphene is moderately thick in anterior view, with its two lateral edges prominent and extending much dorsally, whereas a distinct convex ridge is present at around its mid-level. In dorsal view, the zygosphene is crenate, with distinct lateral lobes (only the right is preserved). The neural spine is thick in dorsal view, moderately high in posterior view, whereas in lateral view, it is relatively thin, much posteriorly inclined, and its height increased toward the posterior portion of the neural arch. The prezygapophyses are almost horizontal in anterior view, with only slight dorsal inclination. The neural arch is much vaulted in posterior view. The cotyle and condyle are large and almost circular. The hemal keel is wide, denoting that the vertebra apparently originates from the posterior trunk region of the column. The posterior median notch of the neural arch is deep in dorsal view. The wide vertebral centrum, being wider than long in ventral view, the paradiapophyses not divided into diapophyses and parapophyses, the reduced prezygapophyseal accessory processes, the deep posterior median notch of the neural arch, as well as the general shape of the vertebra, being robust, strongly built, and higher than long in lateral view, all denote that AMU-CURS-1157 can be assigned to Boidae (Rage 1984, 2001; Szyndlar and Rage 2003).

Within Boidae, the specimen AMU-CURS-1157 bears strong resemblance with the extant genus *Corallus*, in particular its prezygapophyses being horizontally oriented (almost 180°) in anterior view. Other diagnostic characters are the wide, broad, and strongly vaulted neural arch, the crenate zygosphene in dorsal view with a strong median lobe, the zygosphene in anterior view bearing a prominent median ridge and being wider than the cotyle, the presence of small parazygantral foramina, the high neural spine in lateral view, and the absolute vertebral size (neural arch width less than 10 mm) (Rage 2001; Camolez and Zaher 2010; Onary et al. 2018). *Corallus* is still present in Falcón State (Mijares-Urrutia and Arends 2000).

*Eunectes* Wagler, 1830

*Eunectes* sp.

(Fig. 17C1–C5).

*Locality*: SGOP (conglomerate Ly1, Fig. 3C).

*Material*: A trunk vertebra (AMU-CURS-1304).

*General description, comparisons and remarks*: AMU-CURS-1304 is a large specimen with a centrum length of 18 mm. The vertebra is wider than it is long, with a prominent anterior widening of the centrum. The zygosphene is slightly concave in dorsal view and trapezoidal, relatively thick, and with a median tubercle in anterior view. The prezygapophyses are much laterally projected in anterior view. The interzygapophyseal constriction is distinct and defined. The posterior median notch is deep. The cotyle is large and deep. The neural arch is slightly depressed. The hemal keel is moderately thick and crosses the whole midline of the centrum in ventral view. Two prominent and deep subcentral foramina lie at around the middle of the centrum, one at each side of the hemal keel. Similar to the above *Corallus* specimen (AMU-CURS-1157), specimen AMU-CURS-1304 can be assigned to boids on the basis of a wide vertebral centrum, being wider than long in ventral view, the paradiapophyses not being divided into diapophyses and parapophyses, the relatively reduced prezygapophyseal accessory processes, the deep posterior median notch of the neural arch, and also the general shape of the vertebra, being robust, strongly built, and higher than long in lateral view (Rage 1984, 2001; Szyndlar and Rage 2003). Within boids, AMU-CURS-1304 can be referred to the genus *Eunectes*, commonly known as anacondas, on the basis of its rather robust and large size, the slightly depressed neural arch, the thick zygosphene with a median tubercle, the deep interzygapophyseal constriction, and the laterally projected prezygapophyses (see Hsiou and Albino 2009, 2010; Hsiou et al. 2013). The slightly depressed neural arch and the moderately wide hemal keel further imply a position of the vertebra from the posterior or posterior mid-trunk region of the column. Anacondas of the genus *Eunectes* comprise the largest snakes of South America and among the largest worldwide (Murphy and Henderson 1997). Besides the extant species of the genus, another extinct named species has also been referred: †*Eunectes stirtoni* from the middle Miocene of Colombia (Hoffstetter and Rage 1977). Based on the available new material from the San Gregorio Formation, we refrain from assigning this single vertebra to the species level and prefer to refer it to the genus level only. *Eunectes* is currently present in Venezuela only in the Orinoco River basin (Wallach et al. 2014).

Boidae indet.

(Fig. 18A1–B3).

*Locality*: NCC (conglomerate, Fig. 3B).

*Material*: Two isolated vertebrae, one incomplete trunk vertebra (AMU-CURS-1147), and one fragmented specimen of indeterminate position (AMU-CURS-804).

*General description, comparisons and remarks*: AMU-CURS-1147 is small (centrum length: 4.8 mm), missing portions of both postzygapophyses and the edges of both its prezygapophyses, whereas its paradiapophyses and its condyle are much eroded (Fig. 18A1–A5). The neural arch is vaulted in posterior view. The zygosphene is moderately crenate in dorsal view, with two rather prominent lateral lobes, whereas in anterior view it is thin, convex, and wider than the cotyle. The prezygapophyses are dorsally inclined. The neural spine is moderately high, with its base extending across most of the midline of the neural arch. AMU-CURS-804 lacks the posterior ventral part of the centrum (centrum length: ~ 1.2 mm), the right prezygapophysis, both postzygapophyses, most of the posterior portion of the neural arch, the neural spine, and part of the zygosphene (Fig. 18B1–B3). The prezygapophyses are dorsally inclined in anterior view, whereas they are rather slender and extend well anterolaterally in dorsal view. Distinct paracotylar foramina are present. The paradiapophyses are rather eroded but seem not to have been divided into diapophyses and parapophyses. A moderately wide hemal keel (or hypapophysis) is present in the ventral surface of the centrum. The overall shape of these two specimens, being relatively strongly built, with the centrum wider than long in ventral view, the paradiapophyses not divided into diapophyses and parapophyses, the relatively reduced prezygapophyseal accessory processes, suggest that they can be referred to Boidae (Rage 1984, 2001; Szyndlar and Rage 2003). A more precise identification is not possible due to the preservational status of the fossils. It has to be noted that AMU-CURS-1147 bears some resemblance with *Epicrates* Wagler, 1830, especially in terms of the dorsal inclination and lateral expansion of prezygapophyses, the shape of the neural spine, and the overall vertebral shape and size (see e.g., Onary et al. 2018). However, we hesitate to definitively assign this incomplete new fossil specimen to that genus. Nevertheless, an assignment of both specimens to *Corallus* (as was the case of the specimen AMU-CURS-1157) seems to be excluded based on the characters described above, most prominently the much dorsally inclined prezygapophyses of these vertebrae. Specimens AMU-CURS-804 and AMU-CURS-1147 could suggest the presence of at least a second taxon of boids in the NCC assemblage.

?Boidae or ?Aniliidae indet.

(Fig. 18C1–C5).

*Locality*: NCC (conglomerate, Fig. 3B).

*Material*: An incomplete trunk vertebra (AMU-CURS-1158).

*General description, comparisons and remarks*: AMU-CURS-1158 is missing part of the posterior portion of the neural arch and the dorsal part of the neural spine, whereas its cotyle, left prezygapophysis, and both paradiapophyses are strongly eroded. The vertebra is wider than long in ventral view (centrum length: 6.4 mm; neural arch width: 9.1 mm), with its prezygapophyses extending anterolaterally. There is a relatively deep interzygapophyseal constriction. The zygosphene is only slightly crenate in dorsal view, whereas in anterior view it is relatively thin and almost straight to slightly convex. The neural arch is moderately vaulted. The sharp hemal keel in ventral view denotes that the vertebra originates from the mid-trunk region of the column. The overall shape of AMU-CURS-1158, being relatively strongly built, with the centrum wider than long in ventral view, the paradiapophyses not divided into diapophyses and parapophyses, and the relatively reduced prezygapophyseal accessory processes are consistent with a referral to Boidae (Rage 1984, 2001; Szyndlar and Rage 2003). However, AMU-CURS-1158 bears also some resemblance to *Colombophis*, a genus that has been referred to aniliids (Hoffstetter and Rage 1977; Head et al. 2006), or simply treated as a basal alethinophidian (Hsiou et al. 2010), especially †*Colombophis spinosus*, from the middle Miocene of Brazil, Colombia and Venezuela (Hsiou et al. 2010). Features shared between AMU-CURS-1158 and *Colombophis* are the deep interzygapophyseal constriction, the rather pointed and dorsally inclined prezygapophyses, the shape of the zygosphene in anterior and dorsal views, the short prezygapophyseal accessory processes, and the neural spine increasing in height in lateral view much posteriorly from the level of the zygosphene (see figures in Hoffstetter and Rage 1977; Head et al. 2006; Hsiou et al. 2010). Nevertheless, AMU-CURS-1158 can still be differentiated from *C*. *spinosus* by its more vaulted neural arch in posterior view, much more pointed postzygapophyses, more anteriorly inclined prezygapophyses in dorsal view (condition approaching more the type species of *Colombophis*, i.e., †*Colombophis portai* Hoffstetter and Rage 1977), a longer and better defined hemal keel, the orientation of the paradiapophyses, its neural spine not so confined to the posterior portion of the neural arch, and its proportionally much smaller size. Although boid affinities for AMU-CURS-1158 seem to be most likely, based on the existing limited material, we cannot exclude a referral to *Colombophis* or a *Colombophis-*like form.

Caenophidia Hoffstetter, 1939

Colubroides Zaher et al., 2009

Colubroides indet.

(Fig. 18D1–D5).

*Locality*: NCC (conglomerate, Fig. 3B).

*Material*: A fragmentary trunk vertebra (AMU-CURS-1161).

*General description, comparisons and remarks*: A fragmentary trunk vertebra with a centrum length of 1.9 mm (AMU-CURS-1161), missing the zygosphene, its left prezygapophysis, and parts of both postzygapophyses, neural spine, hypapophysis, and right prezygapophysis. The centrum is elongated. A ventrally expanding hypapophysis projects ventrally from the centrum in lateral view, though its exact extent and size cannot be evaluated with certainty. The synapophyses are divided into diapophyses and parapophyses. Paracotylar foramina are present. All the above characters are consistent with the anatomy of Colubroides. Within Colubroides, the presence of a hypapophysis instead of a hemal keel throughout all trunk vertebrae is a characteristic, among others, of most taxa of natricids, elapids, and viperids, whereas a hypapophysis is also present in the anterior trunk vertebrae of “colubrines” (Rage 1984; Szyndlar 2012; Smith 2013; Georgalis et al. 2019). The preservation of the specimen AMU-CURS-1161 does not afford any more precise taxonomic attribution, but it confirms the presence of Colubroides in the fossil assemblage.

Serpentes indet.

(Fig. 18E1–G).

*Locality*: NCC (conglomerate, Fig. 3B).

*Material*: A fragmentary trunk vertebra (AMU-CURS-803), another fragmentary trunk vertebra (AMU-CURS-805), and a fragmented prezygapophysis (AMU-CURS-724).

*General description, comparisons and remarks*: AMU-CURS-803 has a length of ~ 1.3 mm (Fig. 18E1–E4), missing a large part of the neural arch, the right postzygapophysis, most of the left prezygapophysis, and parts of the neural spine and the right prezygapophysis. The most peculiar feature of this vertebra is the high convexity of its thin zygosphene in anterior view, whereas in dorsal view, three distinct lobes are present at the anterior edge of this structure. The specimen AMU-CURS-805 (Fig. 18G) is also a rather fragmentary trunk vertebra missing most of the left prezygapophysis and both postzygapophyses. In a fragmented prezygapophysis (AMU-CURS-724, Fig. 18F) the completely prezygapophyseal articular facet and the prezygapophyseal accessory process are preserved. Due to their poor preservational state, these three specimens are little informative. The relatively long prezygapophyseal accessory process present in AMU-CURS-724 hints at possible affinities with Colubroides, though we refrain from formally assigning this specimen to that group. The overall morphology of AMU-CURS-803, with its relatively wider than long centrum, seems to conform mostly to boids.

Crocodylia Gmelin, 1789 (sensu Benton and Clark, 1988).

Alligatoridae Gray, 1844

Caimaninae Brochu, 2003 (sensu Norell, 1988)

*Caiman yacare* (Daudin, 1802)

*Caiman* aff. *C. yacare*

(Fig. 19A1–A3).

*Locality*: SGOP (conglomerate Ly1, Fig. 3C).

*Material*: An isolated right maxilla fragment (AMU-CURS-1328).

*General description, comparisons and remarks*: There are only a few crocodylian fossils from the San Gregorio Formation so far that can be assigned to a species. Of those, AMU-CURS-1328 is among the best-preserved and identifiable skull remains (Fig. 19A1–A3). The bone bears 14 alveoli and the dorsal bone surface is sculptured with ornamental pitting. Just posterior to the largest alveolus, rostral canthi are not present. Rostral canthi are typical for some species of *Caiman*, such as *C. latirostris* (also for *Melanosuchus niger*), but are absent in others, such as *C. crocodilus* or *C. yacare* (e.g., Norell 1988; Brochu 1999). Anteriorly, there is just a remnant of the suture with the premaxilla, whereas posteriorly, the suture with the jugal is well preserved. The anterior border of the suborbital fenestra is oblique, wide, unlike in *C*. *yacare* and *C*. *crocodilus*, where it is pointed. In *Caiman c*. *apaporiensis* from Colombia, the margin is also oblique but much narrower (Medem 1955). Medially, the bone is broken and the smooth internal narial passage is visible. In lateral view, the outline of the maxilla is wavy. In ventral view, the lateral margin of the maxilla from alveolus 7 to 11 is straight rather than slightly convex, which is noteworthy for a presumably adult specimen.

The first three anterior alveoli are of similar diameter, whereas the following fourth alveolus is much larger. The following ten alveoli are small in diameter and similar sized. Medial to alveoli 2–3, 3–4, 5–6, 6–7, and 7–8, occlusal pits for the dentary dentition can be seen, with the first one being shallow, the following two pits being deeper and the last one being shallow again. These occlusal pits indicate a complete overbite, as is typical for alligatorids, and are indistinguishable from those of extant *C*. *yacare* MLP-R 5044. Alveolus 3 and alveoli 9–13 still carry well-preserved teeth (Fig. 19A2, A3). The third tooth is conical and slightly recurved. The teeth in alveoli 9–13 are straighter and become smaller and more bulbous from anterior to posterior. Bulbous teeth in the posterior portion of the dentary are absent in *C*. *crocodilus apaporiensis* (e.g., Medem 1955; Escobedo-Galván et al. 2015). The anterior teeth in this series are spaced well apart from each other. The last two alveoli might be confluent as there is no bony separation visible. Teeth 3 and 9–11 show anteroposterior carinae, whereas the more bulbous teeth 12 and 13 have a round crown in cross section. AMU-CURS-1328 has a length of 190 mm, indicating that the maxilla derived from a large skull of ca. 400 mm in length (based on comparisons with extant caimanine skulls). AMU-CURS-1328 appears to combine a mosaic of features that could be ancestral to the modern *C*. *yacare*, *C*. *crocodilus*, and *C*. *c*. *apaporiensis*. In the absence of further and more complete specimens, we therefore treat AMU-CURS-1328 as aff. *C*. *yacare*.

Caimaninae Brochu, 2003 (sensu Norell, 1988)

Caimaninae indet.

(Fig. 19B–L).

*Locality*: NCC (conglomerate, Fig. 3B) and SGOP (conglomerate Ly1, Fig. 3C).

*Material*: Twelve cranial and postcranial isolated remains from NCC (AMU-CURS-553, -711, and -1060), and SGOP (AMU-CURS-1315) localities.

*General description, comparisons and remarks*: AMU-CURS-553 includes four crocodylian osteoderms, three of which are flat and one is keeled. The keeled specimen is 23 mm wide and 15 mm long, preserving only the anterior half of the osteoderm (Fig. 19B–D). In this specimen the ornamental pits do not reach the anterior osteoderm margin. The smallest fragment (16 × 17 mm) of the flat osteoderms shows only a few scattered shallow pits on the bone surface and preserves only a small part of the actual bone margin (Fig. 19D). The two-remaining flat osteoderms are of square or almost square shape (26 × 26 mm and 25 × 28 mm), each comprising three strongly sutured margins and one smoother margin (Fig. 19B, C). The two osteoderms differ in the size and distribution of ornamental pits on the bone surface. In one specimen, the largest pits are found distributed along the sutured margins and smaller and less deep pits are scattered over the osteoderm center, whereas in the other specimen, large pits are distributed all over the bone surface with the exception of the thinner, non-sutured margin. Ventrally, all four osteoderms show a cross-hatching pattern of metaplastically ossified structural fibers. The three flat osteoderms are tentatively identified as the posterior ossifications of composite ventral osteoderms prominent in Caimaninae (but see also Brochu et al. 2012 for composite osteoderms in a non-Brevirostres crocodylian).

AMU-CURS-711 comprises two crocodylian osteoderms that were found together. The first one is strongly elongated, with sutural margins, and tapering to anterior and posterior tips. It is 20 mm long and 7 mm wide and carries a low medial ridge (Fig. 19F). The surface of the osteoderm is strongly sculptured by deep pits. The osteoderm likely represents an early stage of a developing dorsal osteoderm (may be from the paravertebral shield), in which the keeled area develops first (Schmidt 1914). The second osteoderm is of rectangular shape (20 mm long and 18 mm wide as preserved) with two sutured margins and one margin tapering into a sharp edge (Fig. 19E). This latter margin of the osteoderm is broken and thus its margin not preserved. The external surface shows a single row of three large and widely spaced pits, opposite the tapering edge margin. Internally, the osteoderm shows some cross-hatching pattern. This osteoderm is identified as the anterior ossification of a composite ventral osteoderm, in which the tapering edge is the anterior margin and the ornamented area is the posterior margin of the ossification. The specimen AMU-CURS-1315 is a partially preserved osteoderm identifiable as the anterior element of a composite ventral osteoderm (Fig. 19G1, G2). This specimen also shows a single row of ventral ornamental pits, three preserved margins with weak sutures, and dorsally a cross-hatching pattern of the bone surface.

AMU-CURS-1060a-b includes an assortment of crocodylian remains, including a skull fragment, one cervical rib, and three fragmentary osteoderms. The skull fragment (AMU-CURS-1060b, Fig. 19L), a right postorbital, has a smoothly convex anterolateral margin, an opposite concave posteromedial margin (forming the margin of the supratemporal fenestra), and shows strong ornamental pitting on its dorsal surface. In anterolateral view, the sutural contacts with the frontal anteriorly and parietal medially and the squamosal posteriorly are preserved, as well as the foramen for the superficial temporal artery (Holliday and Witmer 2007). The bone is 31 mm long (maximum anteroposterior length) and 23 mm wide (maximum mediolateral width). The cervical rib is 31 mm long and 8 mm high and shows the typical double articulation with ventral capitulum and a more dorsally situated tuberculum (AMU-CURS-1060b, Fig. 19K). Of the osteoderms (AMU-CURS-1060a, Fig. 19H–J), two are of rectangular shape and flat, one being the anterior element and the other being the posterior element of a composite ventral osteoderm. Both elements show a crosshatching pattern and few scattered nutrient foramina on the internal bone surface. Whether both elements form a single unit, however, is not clear. The posterior ossification (22 × 13 mm as preserved) shows three sutured and one broken margin and only small, scattered pits (and one larger pit) over its external bone surface. The anterior ossification (26 × 18 mm) shows three sutured margins, and one margin tapering to a smooth sharp edge. A single row of five pits filled with sediment is present opposite the tapering margin. One small-keeled osteoderm shows two rows of parasagittally arranged pits, with those on one side of the keel being slightly larger than those on the other side (Fig. 19J). This osteoderm is 20 mm long and 16 mm wide. Ventrally it shows a single nutrient foramen. The remaining four osteoderms (or partial skull bones) are very fragmentary. They show strong ornamentation of the bone surface in the form of pitting, but otherwise lack sutural contacts or other diagnostic features.

Crocodylia indet.

(Figs. 20A1–T2 and 21A–P).

*Locality*: NCC (conglomerate, Fig. 3B) and SGOP (conglomerate Ly1, Fig. 3C).

*Material*: Over 583 isolated remains, including 524 teeth [522 from NCC (AMU-CURS-19, -167, -302, -558, -574–577, -666, -707, -829, -847, -861, -881–882, -1095, -1121, -1129, and -1201) and 2 from SGOP (AMU-CURS-1322 and -1352)], 35 osteoderms [28 from NCC (AMU-CURS-30, -594, -737, -830, -884, -1029, -1125, -1184, and -1236) and 7 from SGOP (AMU-CURS-1311–1316 and -1321)], and 24 indeterminable skull, lower jaw, and other postcranial bone fragments [21 from NCC (AMU-CURS-21, -561, -578, -593, -743, -826, -1030, -1062, -1082, -1200, and -1236) and 3 from SGOP (AMU-CURS-1309, -1320, and -1319)].

*General description, comparisons and remarks*: Many postcranial bones and teeth are recovered from the San Gregorio Formation, but in contrast to larger or more complete cranial elements, these are seldom diagnostic to the generic or specific level. Teeth are represented in different sizes and shapes, ranging from a few millimeters to 63 mm long and 28 mm wide at the base for the largest specimen (e.g., AMU-CURS-1057). Most of the teeth are well preserved with slender, curved, massive, and conical sharp crowns, showing carinae, ornamental ridges, or fine ornamental rugosities of the enamel surface (Fig. 20A1–H). Some tiny curved crocodylian teeth (AMU-CURS-1095, Fig. 20I1, I2) of about 2 and 4 mm in length, with lateral carinae and rugose surface wrinkles on the enamel crown, which might be from a hatchling or very young juvenile specimens.

The osteoderms in general are well preserved, and here the most representative specimens are described. AMU-CURS-030 and AMU-CURS-033 comprise two keeled paravertebral osteoderms of sub-square shape (Fig. 20J1–K2). The surface ornamentation of the osteoderms consists of round to ovoid pits that extend over the complete dorsal surface. Ventrally the osteoderms show scattered nutrient foramina and a strong cross-hatching pattern of metaplastically ossified structural (collagenous) fibers of the deep connective tissue underlying osteoderms of the paravertebral shield (the cingular ligament; see Salisbury and Frey 2001). The osteoderms show sutured margins, with the medial margin being thickened, indicating a close contact with an adjacent osteoderm. AMU-CURS-030 and AMU-CURS-033 could derive from the medial rows of the paravertebral shield (Frey 1988). AMU-CURS-593 contains a fragmentary osteoderm with a tilted keel and a small carinated tooth (13 mm in length) missing the very tip of the crown (Fig. 20L1, L2). The shape of the base of the osteoderm is not discernible due to the lack of preserved marginal areas. AMU-CURS-594 comprises three osteoderms (Fig. 20M, N, Q), of which the largest one is complete and the other two only partially preserved. The smallest specimen (17.5 × 15.5 mm) is flat and of rectangular shape. The preserved margins show sutures and the external surface is sculptured with pits. The mid-sized, only partially preserved specimen and the largest specimen (43 × 36 mm) are keeled osteoderms of ovoid shape. The ornamentation consists of irregularly arranged, larger and smaller pits that reach the margins. The three specimens all show a cross-hatching pattern and scattered nutrient foramina on their internal/visceral bone surface.

AMU-CURS-830 is a single osteoderm of roughly ovoid shape and an off-centered peak (Fig. 20R). Although overall preservation is not great in this specimen, the margins of the osteoderm carry pegs and sockets, indicating sutured margins on all sides. Ornamentation consists of irregularly arranged larger and smaller pits that reach up to the bone margins. Based on the presence of the off-centered peak, the osteoderm could be from an accessory row on the trunk or from the tail of the animal.

AMU-CURS-1184 is an osteoderm with sub-rectangular base (62 × 78 mm) and a medial keel (Fig. 20P). The posterior margin and one of the lateral margins of the osteoderm show stronger sutures, whereas the sutures are less developed on the anterior and opposite lateral margins. Ventrally, a strong cross-hatching pattern is visible. AMU-CURS-1185 is a keeled osteoderm with a rectangular shape. The lateral margins comprise thickened sutures, indicating articulation with adjacent medially and laterally positioned osteoderms. The bone surface is sculptured with deep pits, except the anterior margin, which remains as a free anterior bar. This specimen (36 mm long × 32 mm wide) pertains to the dorsal paravertebral shield. Due to gypsum incrusting, a cross-hatching pattern and few scattered nutrient foramina are only weakly visible in the ventral bone surface.

AMU-CURS-1311 is a partial osteoderm with an ovoid base and medial keel (Fig. 20O1, O2). Ventrally a slight cross-hatching pattern and a few small foramina are discernible. AMU-CURS-1312 (Fig. 20T1, T2) and -1314 (Fig. 21B) are partial osteoderms with rectangular bases and medial keels. The anterior dorsal margins of the osteoderms taper to a sharp edge and lack ornamental pitting. The ventral base of both osteoderms is slightly concave. A weak cross-hatching pattern and few small foramina can be seen on the ventral bone surface. AMU-CURS-1313 (Fig. 20S) comprises a strongly eroded partial osteoderm with medial keel that lacks the lateral margins, and AMU-CURS-1316 is a strongly weathered flat osteoderm of rectangular shape (Fig. 21C).

Most cranial and postcranial bones are poorly preserved. Cranial bones are represented by small portion of the left dentary (AMU-CURS-1320, Fig. 21D1, D2), possibly preserving the posterior margin of the fourth alveolus to the anterior margin of the seventh alveolus (the fourth one being much larger than the others), and a small portion of either skull or lower jaw (AMU-CURS-1321, Fig. 21E), with strong sculptured pitting pattern on the external bone surface. AMU-CURS-1200 is a fragment that derives from the skull roof and preserves a part of the dorsal and posterodorsal orbital rim of a frontal sutured to a small anterior part of the parietal (Fig. 21F). The dorsal surface of both bone fragments shows ornamental pitting and a smooth ventral bone surface. The frontal part also shows a small foramen laterally. AMU-CURS-1309 comprises the posterior part of a surangular (Fig. 21G).

Postcranial bones include fragmented vertebrae, ribs, and limbs. AMU-CURS-21 is well-preserved vertebral centra with cotyle and condyle articulations (Fig. 21I1, I2). Anteroventrally, partially preserved hypapophyses and lateroventrally, elongated parapophyses that extend along the anterior margin of the centrum are present, which indicates that these specimens are from the anterior (postaxial) cervical vertebrae. The centrum is 23 mm long, 14 mm wide, and 17 mm high (anteriorly). AMU-CURS-578a corresponds to a centrum of a dorsal vertebra with a condyle and a cotyle as articular surfaces (Fig. 21L1, L2), and one rib fragment (Fig. 21P). The vertebral centrum is 25 mm long and 17 mm wide at mid-centrum. Ventrally, the centrum has a straight and not a concave border and thus represents one of the posterior centra in the dorsal (lumbar) series. The rib fragment (AMU-CURS-578b) is 84 mm long and 12.5 mm wide at the widest and flattened expansion of the rib shaft. AMU-CURS-743 is an isolated humeral shaft (Fig. 21H), where most of the proximal head and distal epiphysis are not preserved, so that the fourth trochanter and a small foramen constitute the only identifiable shaft features. AMU-CURS-826 is a well-preserved vertebral centrum (Fig. 21J1, J2) with cotyle and condyle articulations similar in shape to AMU-CURS-21. AMU-CURS-887 is an isolated vertebral centrum from the dorsal series. The centrum has a cotyle and a condyle and dorsally, the facets for the neural arch and the floor of the neural canal are visible, and zygapophyses are not preserved. AMU-CURS-1030 is another isolated crocodylian vertebral centrum with a cotyle and condyle articulation (Fig. 21K1, K2). The centrum is 41 mm long and 22.5 mm at mid-centrum. This specimen also comes from the posterior part of the dorsal (lumbar) vertebral series. AMU-CURS 1062 comprises two vertebrae of different sizes showing the proximal bases of hypapophyses, and are thus identified as pertaining to the cervical series (Fig. 21M1–N). The smaller vertebra has well-preserved zygapophyses and transverse processes, but the neural spine and the condylar and cotylar articulations of the centrum are strongly weathered (Fig. 21M1, M2). The larger specimen has a better-preserved centrum, but the neural spine and the zygapophyses—with the exception of the left postzygapophysis––are not preserved (Fig. 21N). AMU-CURS-1236 corresponds to an isolated prezygapophysis and five four partially preserved osteoderms, which were not found in association to each other (Fig. 21O). The articulation facet of the isolated prezygapophysis is 15 mm long and 8 mm wide. The very fragmentarily preserved osteoderms show strong ornamentation of the external bone surface in the form of pitting, but otherwise lack sutural contacts or other diagnostic features.

Mammalia Linnaeus, 1758

Metatheria Huxley, 1880

Didelphimorphia Gill, 1872

Didelphidae Gray, 1821a

*Didelphis* Linnaeus, 1758

cf. *Didelphis* sp.

(Fig. 22A1–A3).

*Locality*: NCC (conglomerate, Fig. 3B).

*Material*: A partial right humerus (AMU-CURS-1140).

*General description, comparisons and remarks*: AMU-CURS-1140 has a length of 22 mm, preserving a short portion of the shaft and distal epiphysis, where the entepicondylar foramen (Fig. 22A2, A3), the humeral coronoid fossa (Fig. 22A1), olecranon fossae, and medial epicondyle (Fig. 22A2, A3) are preserved. AMU-CURS-1140 has certain similarities with the humerus of extant species of *Didelphis*; however, the fragmentary nature of the fossils does not permit an identification beyond generic level.

Placentalia Owen, 1837

Xenarthra Cope, 1889

Phyllophaga Owen, 1842

†Megatheriidae Gray, 1821b

†Megatheriinae Gray, 1821b

†*Proeremotherium* Carlini, Brandoni and Sánchez, 2006b

cf. †*Proeremotherium* sp.

(Fig. 22B1–C3).

*Locality*: NCC (conglomerate, Fig. 3B) and SGOP (conglomerate Ly1, Fig. 3C).

*Material*: A nearly complete skull of a ground sloth (AMU-CURS-184), collected from the sandstones overlying the conglomeratic layer in the NCC locality, and an isolated tooth (AMU-CURS-1303) from the SGOP locality.

*General description, comparisons and remarks*: AMU-CURS-184 is a relatively well-preserved skull with a total length of 455 mm, lacking the jugals, the premaxillae, the left zygomatic process of the squamosal, vertical lamina of the left pterygoid, anterior part of the nasals, anterior part of the maxillae, lateral and partial anterior wall of the alveoli of right Mf1, lateral and anterior wall of the alveoli of left Mf1, and the lateral wall of those of the left tooth row, and teeth (Fig. 22B1–B3). AMU-CURS-184 is broadly similar in size and morphology to that of †*Proeremotherium eljebe* from the underlying Codore Formation in the Urumaco Sequence (Carlini et al. 2006b), but differing in several features such as a longer basicranial area and a more posteriorly projected basioccipital between the condyles (see for details Carlini et al. 2018). The presence of this specimen assigned to cf. *Proeremotherium* in the San Gregorio Formation documents a northern Neotropical occurrence of a megatheriine that addresses issues on intraspecific variation and biogeography (Carlini et al. 2018).

The isolated tooth AMU-CURS-1303 is an incomplete left molariform Mf3 (42 mm height) of indeterminate position (Fig. 22C1–C3). The molariform lacks enamel and it is almost quadrangular in shape (slightly wider than long), with well-marked corners. Although in occlusal view AMU-CURS-1303 is broken and transversal hard dentine (orthodentine) layers are still visible, the two well-developed transversal crests of orthodentine, separated by a deep “V”-shaped valley that characterize cheek teeth (except Mf5) of megatherids (see Carlini et al. 2006b; Bargo et al. 2012), are not preserved.

†Mylodontidae Gill, 1872

†Mylodontidae indet.

(Fig. 23A1–A3).

*Locality*: NCC (conglomerate, Fig. 3B).

*Material*: A right humerus (AMU-CURS-62).

*General description, comparisons and remarks*: The specimen AMU-CURS-62 is approximately 450 mm in length. It was collected in the sandstones overlying the conglomeratic layer in the NCC locality (Fig. 2E). As in other mylodontids, like †*Bolivartherium*, †*Lestodon*, or †*Glossotherium*, AMU-CURS-62 has a head that slightly exceeds the height of the major and minor tuberosities (Fig. 23A1, A2), and does not markedly projected posteriorly. The major tuberosity is slightly larger and more massive than the minor one. The diaphysis is a little wider than in specimens known of the above-mentioned genera. The pectoral and deltoid crests are prominent, placed at the mid-shaft, forming a “V” pointed distally and aligned with the main diaphyseal axes (not as in scelidotherines, where these structures are diagonally set at the beginning of the distal third of the diaphysis). The entepicondylar foramen is absent and the pronator and supinator processes are not strongly developed but broad, forming a wide and flattened distal third. The projected line that joins its medial most and lateral-most projections being oblique with respect to the line that joins the tuberosities. The olecranial fossa is not deep (Fig. 23A2), and the distal line of the trochlea is slightly concave. At the distal articular surface, the condyle is bigger mediolaterally than the trochlea.

Cingulata Illiger, 1811

†Glyptodontoidea Gray, 1869

†Glyptodontidae Gray, 1869

†*Boreostemma* Carlini et al., 2008b

aff. †*Boreostemma* sp.

(Fig. 23B, C).

*Locality*: NCC (conglomerate, Fig. 3B).

*Material*: Two osteoderms of the carapace region (AMU-CURS-1242).

*General description, comparisons and remarks*: The osteoderms AMU-CURS-1242 resemble those of *Boreostemma* from the underlying Codore Formation (Pliocene) (see Carlini et al. 2008b); however, the poor preservational condition does not allow a more accurate taxonomic assignation. The complete specimen, which is 43 mm in length (Fig. 23B), can be assigned here to dorsal or postero-dorsal osteoderm of the carapace region. The second specimen is incomplete and precludes a determination of the carapace region (Fig. 23C). The specimens AMU-CURS-1242 correspond to the osteoderms referred previously to aff. †*Boreostemma codorensis* by Vucetich et al. (2010). These osteoderms were not illustrated by Vucetich et al. (2010), and the taxonomical reference by these authors as aff. *B*. *codorensis* is incorrect, as the correct name of the species is †*Boreostemma pliocena* (see Carlini et al. 2008b). Other specimens assigned to *Boreostemma*? from the NCC locality included at least 14 osteoderms reported by Carlini et al. (2008c) and Zurita et al. (2011).

†Pampatheriidae Paula Couto, 1954

†*Holmesina* Simpson, 1930

†*Holmesina floridanus* Robertson, 1976

aff. †*Holmesina floridanus*.

(Fig. 23D1–G2).

*Locality*: NCC (conglomerate, Fig. 3B) and SGOP (conglomerate Ly1, Fig. 3C).

*Material*: Four carapace osteoderms, including two incomplete movable osteoderms from the NCC (AMU-CURS-1063 and -1119) and two fixed osteoderm of pelvic buckler from SGOP (AMU-CURS-1294 and -1295) localities.

*General description, comparisons and remarks*: Vucetich et al. (2010) referred some osteoderms assigned to aff. *Holmesina floridanus* for the NCC locality; however, the specimens were not illustrated. The new pampathere osteoderms collected in the San Gregorio Formation and referred herein belong to a new taxon (sp. 1) under study (in prep.) and aff. to *Holmesina floridanus* (the oldest species recorded as a pampathere in North America) (Edmund 1987; Scillato-Yané et al. 2005; Carlini and Zurita 2010).

The specimens described herein (Fig. 23D1–E2) are thick, with a peripheral area lower than the main exposed surface, with one or two rounds of depressions that opens radially; the surface is rugose in appearance because of several punctures, on the exposed main surface clear centra area elevated, flanked by two shallow and wide depressions. The evidence suggests that a †*Kraglievichia*/*Holmesina* (or a related intermediate taxon) would have migrated to North America during the GABI, and there the genus *Holmesina* would have differentiated with a single species (*H. floridanus*) for the Blancan (and Irvingtonian?), and other species for the Rancholabrean (†*Holmesina septentrionalis*, that was sometimes included as a taxon in the Blancan because of labels in collections) (Carlini and Zurita 2010). Similarities between specimens AMU-CURS-1063 (Fig. 23D1, D2), -1119 (Fig. 23E1, E2), -1294 (Fig. 23G1, G2) and -1295 (Fig. 23F1, F2), from the San Gregorio Formation and osteoderms of *H*. *floridanus* (FLMNH-UF 223813) from the late Blancan late Pliocene of North America, are evident, which support the hypothesis that *Holmesina* is linked to the *Kraglievichia* lineage (Carlini and Zurita 2010), provided these similarities are indeed indication of close relationships. The sequence †*Kraglievichia paranense* (late Miocene, Tortonian) (Cione et al. 2000)—new “sp.1” of the San Gregorio Formation—*H. floridanus* (late Blancan, late Pliocene) may represent an anagenetic series.

†*Plaina* Castellanos, 1937

aff. †*Plaina* sp.

(Fig. 23H1, H2).

*Locality*: NCC (conglomerate, Fig. 3B).

*Material*: An isolated and complete fixed osteoderm of pelvic buckler (AMU-CURS-736).

*General description, comparisons and remarks*: AMU-CURS-736 (sp. 2) is 35 mm in length, resembling those osteoderms of *Plaina* sp. from the Pliocene of Northwestern Argentina (Gois 2013; Góis et al. 2013; Bonini 2014). It is because AMU-CURS-736 has an almost flat and smooth exposed surface with few punctuations, and with a shallow and wide depression, that surrounds a slightly elevated and rounded central area (Fig. 23H1). AMU-CURS-736 is thinner (Fig. 23H2) than those of the specimens “sp. 1” referred above to aff. *Holmesina* (AMU-CURS-1063, -1294 and -1295). AMU-CURS-736 is close to half of its thickness for an equivalent surface (e.g., Fig. 23D2, E2, F2 and G2). The presence of this eventual new “sp. 2” in the NCC locality would represent a second lineage of pampatheres.

Xenarthra indet.

(Fig. 23I1–I3).

*Locality*: SGOP (conglomerate Ly1, Fig. 3C).

*Material*: An incomplete caudal vertebra (AMU-CURS-1356).

*General description, comparisons and remarks*: AMU-CURS-1356 corresponds to a caudal vertebra of the anterior region of the series with a centrum of 57 mm in diameter. The right transverse process, hemal facets, and neural arch are preserved; however, the articular facets and spinous process are missing. The right transverse process in AMU-CURS-1356 projects so far ventrally, a morphological feature observable also in some glyptodonts (see Gillette and Ray 1981, figs. 76, 77). Nevertheless, due to the preservational condition of AMU-CURS-1356, for now, we refrain from assigning this specimen to either a terrestrial sloth or a glyptodont.

†Meridiungulata McKenna, 1975

†Litopterna Ameghino, 1889

†Proterotheriidae Ameghino, 1887

†Proterotheriidae indet.

(Fig. 24A1–B2).

*Locality*: NCC (conglomerate, Fig. 3B).

*Material*: A metacarpal (AMU-CURS-742) and a distal epiphysis of a metacarpal (AMU-CURS-1189).

*General description, comparisons and remarks*: The proterotheriid elements assigned here as a metacarpal (Fig. 24A1–A3) and a distal epiphysis of a metacarpal (Fig. 24B1, B2), likely belonged to a juvenile individual as the epiphysis did not fuse to the shaft. Due the fragmentary condition of these specimens, it is not possible to determine a more precise taxonomic identification. Carrillo et al. (2018) reported proterotheriid specimens from the Algodones Member of the Codore Formation, the new specimens here reported from the NCC locality extend the stratigraphic record to the Vergel Member of the San Gregorio Formation.

†Notoungulata Roth, 1903

†Toxodontidae Gervais, 1847

†Toxodontinae Trouessart, 1898

†*Falcontoxodon* Carrillo et al., 2018

†*Falcontoxodon* sp.

(Figs. 24C1–K2 and 25A1–E3).

*Locality*: NCC (conglomerate, Fig. 3B).

*Material*: Thirty-three dental and postcranial elements, including two upper incisors (AMU-CURS-825 and -1335), two upper premolars (AMU-CURS-1331 and -1332), two upper molars (AMU-CURS-1333 and -1346), five lower incisors (AMU-CURS-828, -888, -1339, -1342, and -1350), 14 lower premolars (AMU-CURS-831–835, -846, -889, -1334, -1336–1338, -1340–1341, and -1343), five lower molars (AMU-CURS-1344–1345 and -1347–1349), two astragali (AMU-CURS-1202 and -1330), and one metatarsal (AMU-CURS-1118).

*General description, comparisons and remarks*: We report 33 additional dental and postcranial elements of *Falcontoxodon* sp., a taxon reported from the same locality by Carrillo et al. (2018). The second upper incisor (I2) is developed as a tusk; it is triangular in cross section with enamel in the mesial and part of the labial side of the crown (Fig. 24C1–C3). The upper premolars (P4) show an enamel band on the labial side of crown, a second one on the mesiolingual side and a lingual enamel fold with a narrow enamel band (Fig. 24E1, E2). The upper molars are identified as M1 or M2 (Fig. 24F1, F2) because of the absence of a lingual column in the protoloph (Carrillo et al. 2018). They have a primary lingual enamel fold and one broad enamel band on the labial side of the crown and two narrow bands, one on the mesial and one on the lingual side.

The lower incisors (Fig. 24G1–H3) have a broad labial enamel band and a narrow lingual band. In one specimen (AMU-CURS-1342), there is a small lingual enamel fold. The lower premolars (Fig. 24I1–K2) have an enamel band only on the labial side. The specimens with a labial groove are tentatively identified as p4, which is absent in the p3 of the holotype of *Falcontoxodon* (Carrillo et al. 2018)*.* The lower molars (identified as m1 or m2) have a buccal enamel fold on the labial side of the crown, and a meta-entoconid and ento-hypoconulid fold on the lingual side (Fig. 25A1–B3). There is a lingual enamel band that extends from the anterior fold to the hypoconulid, as in the holotype of *Falcontoxodon* (Carrillo et al. 2018)*.* The new postcranial elements include two astragali (AMU-CURS-1202 and -1330, Fig. 25C1–D2) and a metatarsal IV (AMU-CURS-1118, Fig. 25E1–E3). The neck of the astragali is very short and the medial tibial facet is expanded medially, as in the *Falcontoxodon* astragalus described from the same locality (Carrillo et al. 2018). However, in the new astragali, specimens have the sustentacular and navicular facets separated, unlike the astragalus previously described, which could be related to intraspecific or ontogenetic variation.

†Toxodontinae indet.

(Fig. 25F1–H2).

*Locality*: SGOP (conglomerate Ly1, Fig. 3C).

*Material*: An upper incisor (AMU-CURS-1326), an upper left molar (AMU-CURS-1325), and a right partial mandible (AMU-CURS-1351).

*General description, comparisons and remarks*: The upper left molar AMU-CURS-1325 (Fig. 25H1, H2) is ~ 80 mm in length, and the right partial mandible (AMU-CURS-1351) of ca. 155 mm in length with m1–m3. The lower molars in AMU-CURS-1351 have a buccal enamel fold, and on the lingual side, the m1 and m2 have a meta-entoconid and an ento-hypoconulid fold (Fig. 25F1, F2). Parts of the crowns are broken, including enamel bands on the lingual side.

†Typotheria Zittel, 1893

†Interatheriidae Ameghino, 1887

†Interatheriidae indet.

(Fig. 26A1–A5).

*Locality*: NCC (conglomerate, Fig. 3B).

*Material*: an isolated crown (AMU-CURS-818).

*General description, comparisons and remarks*: AMU-CURS-818 is an isolated unworn crown of the left m3 with a length of 4.7 mm. The crown has a distinctive elongated talonid (Fig. 26A1), is nearly hypsodont, and lacks cementum. On the occlusal surface, the crown is lophodont and displays a straight and long transverse metacristid with no distinct longitudinal projection. The trigonid has a distinctive labial reduction of the anterolingual cristid. A deep lingual sulcus separates the paraconid from the metaconid. The metacristid of the metaconid is distinct and projects lingually. The protoconid is connected to the paraconid by a longitudinal ridge. Similar to the m3 of other typotheres, the talonid attaches to the trigonid about midway along the length of the transverse metacristid and forms a labially convex crescent. A discontinuous cingular segment is located distolabially from the hypoconulid. There is a strong mesiolingual cingulid connecting the base of the paralophid with the base of the metaconid. The cristid obliqua is short, low, and rounded and runs parallel to the tooth row.

AMU-CURS-818 is referred to the Notoungulata based on it having a lophodont dentition with two main crescentic crestids (the metalophid and the hypolophid) together with a shorter transverse entolophid derived from the entoconid. The crown lacks cementum and is hypsodont, a distinctive morphology not present in any small-sized notoungulate. It lacks the distinctive tube-like margins present in hypselodont interatheriids with cementum (e.g., †*Miocochilius*). The unreduced second lobe present in the m3 rules out any hegetotheriidae relationships (Cerdeño and Reguero 2015), an interpretation also supported by the lack of cementum, and having a less reduced posterior lobe on m3, the latest only present in Neogene interatheriids. The relative depth of the labial valley between the trigonid and talonid rules out any relationship with other Neogene notoungulate reported in tropical South America. Finally, the presence of an anterolingual cingulid, only preserved in Paleogene †*Notostylops*, suggests an earlier divergence from primitive interatheriids. This small notoungulate has a distinct reduced talonid, and a labially reduced crest connecting the paralophid with the protoconid, features only present in Paleogene notoungulates. The m3 lacks the elongated second lobe present in Neogene mesotheriine specimens, e.g., †*Miocochilus anomopodus* and †*Protypotherium* (Rose 2006; Renvoisé and Michon 2014; Tapaltsyan et al. 2015). The nearly hypsodont crown with no cementum suggests a non-notohippine ancestry (Wyss et al. 2018).

AMU-CURS-818 from the NCC locality is one of the youngest interatheriid fossils in South America. Despite its clear association with fluvial depositional paleoenviroments, the shape of the crown suggests little to no transport prior to burial (Fig. 26A1–A5). However, a distinctive hypselodont dentition cannot be identified in our reduced sample (*N* = 1). Despite evident sampling biases, many of these hypselodont dental morphologies are distinctive of Neogene interatheriids, such as *Miocochilius* or *Protypotherium*. In contrast, the occurrences of notostylopids (typotheres) are restricted to Paleogene sequences in Argentina, Brazil, and Chile (McKenna and Bell 1997; Billet 2011). The absence of cementum rules out any relationship with the Interatheriinae (Vera et al. 2017), while the crown lacks the distinctive flat labial face present in hegetotheriids (Cerdeño and Reguero 2015). The anteroposterior elongation of the second lobe rules out any relationship to basal hegetotheriids, while the m3 has a relatively deep labial sulcus on m3 not present in basal Interatheriinae such as †*Santiagorothia* and †*Proargyrohyra*x (Cerdeño and Reguero 2015).

†Typotheria inc. sed.

(Fig. 26B1–B3).

*Locality*: NCC (conglomerate, Fig. 3B).

*Material*: A partial jaw (AMU-CURS-1190).

*General description, comparisons and remarks*: AMU-CURS-1190 is a partial jaw with fused symphysis of 16.0 mm in length and 13.5 mm in width. The specimen lacks all tooth crowns (Fig. 26B1) but the alveoli for lower i1 and right i1-p2 are preserved. A mental foramen is located about 5.0 mm below the base of the crown for the p1 (Fig. 26B3). The posterior end of the symphysis is located below the root of the p1 (Fig. 26B2). The symphysis is shallower than that of small-sized typotheres, like the hegetotheriid †*Hemihegetotherium trilobus* (Croft and Anaya 2006), and resembles the more gracile Eocene typothere genus †*Griphitherion* from northwestern Argentina (García and Powell 2011). AMU-CURS-1190 is tentatively referred as a Typotheria inc. sed. based on its small size, shallow symphysis, and completely fused mandible.

Rodentia Bowdich, 1821

Hystricognathi Tullberg, 1899

Caviomorpha Wood, 1955

Cavioidea Fisher von Waldheim, 1817 (sensu Kraglievich 1930)

Caviidae Fisher von Waldheim, 1817

Hydrochoerinae (Gray, 1825a, b) Gill 1872: Weber 1928 (sensu Kraglievich 1930)

†*Hydrochoeropsis* Kraglievich, 1930

?†*Hydrochoeropsis wayuu* Pérez et al., 2017

(Fig. 27A1–B2).

*Locality*: NCC (Fig. 3B).

*Material*: A right dentary (AMU-CURS-744) and a left M1 or M2 (AMU-CURS-1222).

*General description, comparisons and remarks*: The dentary of AMU-CURS-744 is ca. 89 mm in length (Fig. 27A1, A2); it was recovered from the fine sandstone layer about 20 cm below the conglomerate (Fig. 3B). AMU-CURS-744 is quite damaged, but the occlusal surface of the cheek teeth is relatively well preserved (Fig. 27A1, A2). On the medial surface of the dentary, the incisor alveolus forms a salience extended up to the m1 (Fig. 27A2). A prominence projects from the posterior most region of the alveolus in posterior direction, up to the level of the m3, where it is located the mandibular foramen, which penetrates the dentary obliquely (Fig. 27A2). In the lateral view, a small portion of the condylar process is preserved posteriorly to the level of the m3.

The cheek teeth are euhypsodont, formed by prisms or laminae (Fig. 27A1). From the dental series, the p4 and m3 are the best-preserved teeth. The p4 shows the anterior secondary prism (pr.s.a.) with a rounded mesial outline and the tip of the 3rd internal column (c.3e) is oriented distolabially. Lingually, the pr.s.a. shows a wide and shallow 5th internal flexid (h.5i). The secondary external flexid (h.s.e.) is wide and is penetrating obliquely. The pr.s.a. is connected to the pr. I by a thin and short isthmus. The pr. II and I are “y-shaped,” both connected by a short mesiodistally oriented isthmus. The columns of these prisms are transversely oriented, being the tip of the 3rd internal column (c.3i.) located at the same level as the 2nd external column (c.2e.). On the lingual region of the P I and P II, the 3rd internal flexid (h.3i) and 2nd internal flexid (h.2i) have the same deepness, reaching the midline of the tooth, while the 1st internal flexid (h.1i) is more developed, surpassing the midline. The tip of the h.2i is opposite to the fundamental external flexid (h.f.e). The h.f.e. is wide like the h.s.e. but is less deep. The tip of the 1st external column is broken.

In the m1, from the pr. I only the pr. Ib is preserved (Fig. 27A1). The lingual tip of this prism is connected to the pr. II. The lingual tips of the pr. IIa’ and IIa’’ are broken. The labial tip of the pr. IIa is connected to the pr. IIb. In the m2, only the pr. I is well preserved (Fig. 27A1). The pr. I’ and pr. II’ are transverse laminae, labially connected, separated by a labiolingually wide tertiary internal flexid (h.t.i.). The m3 is composed of transverse prisms (Fig. 27A1). The pr. I is “U-shaped” and does not preserve the lingual tip of the pr. I’’. The pr. I’ and pr. I’’ are labially united. The pr. II is quite damaged, not preserving the tips of the pr. IIa and pr. IIb’’.

The cheek teeth of AMU-CURS-744, as well as the specimen AMU-CURS-1222, were compared with other Neogene and Quaternary hydrochoerines (Vucetich et al. 2005, 2012, 2014, 2015; Deschamps et al. 2007; Pérez et al. 2017; Gomes et al. 2019; Cerdeño et al. 2019). Two morphological traits of the p4 let us to assign AMU-CURS-744 to the hydrochoerine ?*Hydrochoeropsis wayuu*, a taxon recently described from the Pliocene of Colombia (Pérez et al. 2017): (1) the h.2i and h.3i are equally deep (also shared with *Hydrochoeropsis dasseni* from the Pliocene of Argentina); and (2) the fifth internal flexid (h.5i) has the same wide and depth as in ?*H*. *wayuu*, differing from other known hydrochoerines (see Pérez et al. 2017: p. 115). Besides, the p4 exhibits a symmetric pr. II, with the 3rd internal column at the same level as the 2nd external column, and the tip of the h.2.i opposite to the h.f.e, which are diagnostic traits of this Pliocene hydrochoerines. The only lower tooth of the type series of ?*H. wayuu* is a fragmented p4. Therefore, the material here described is the most complete lower dental series assigned to this species.

The isolated upper tooth (left M1 or M2) AMU-CURS-1222 of 21.6 mm in length (Fig. 27B1, B2) was collected in a fine sandstone layer about 60 m south of the conglomerate outcrop. Although this layer belongs to the Vergel Member and it is included in the same area that we call NCC locality, stratigraphically it could be located about 30 m below the conglomerate. AMU-CURS-1222 is similar to the specimen MUN-STRI-16233, described by Pérez et al. (2017).

The fossil record of hydrochoerines from NCC locality includes †*Cardiatherium* sp. (see Vucetich et al. 2010). However, with the description of ?*H*. *wayuu* from the Ware Formation, Pérez et al. (2017) suggested that the remains reported by Vucetich et al. (2010) could belong to young specimens of this species. Hence, the specimens here described confirm the presence of ?*H. wayuu* in the San Gregorio Formation and reinforces the biostratigraphic correlation between the two geological units (Moreno et al. 2015).

Hydrochoerinae indet.

(Fig. 27C1–E2).

*Locality*: NCC (conglomerate, Fig. 3B).

*Material*: Three fragmentary teeth (AMU-CURS-1186–1188).

*General description, comparisons and remarks*: The specimens correspond to fragmentary laminar cheek teeth (AMU-CURS-1186, pr. IIa’’ and pr. IIb of a right m3; AMU-CURS-1187, ?pr. IIa and pr. IIb’ of left m1;AMU-CURS-1188, ?pr. 1 of a left M1 or M2), whose state of preservation does not allow a more precise taxonomic determination.

Chinchilloidea Bennett, 1833

†Neoepiblemidae Kraglievich, 1926

†Neoepiblemidae indet.

(Fig. 27F1, F2).

*Locality*: NCC (conglomerate, Fig. 3B).

*Material*: An isolated left dp4 (AMU-CURS-1220).

*General description, comparisons and remarks*: AMU-CURS-1220 corresponds to a left dp4 with 10 mm in length assigned to a neoepiblemid rodent (Fig. 27F1, F2). It is a mesiodistally elongated and laminar tooth, with signs of resorption in the apical portion. The occlusal surface is composed of four oblique laminae (Fig. 27F1). The first and second laminae are labially connected. A lingual flexid penetrates between both laminae obliquely, but it does not reach the midline of the tooth. Labially to the labial tip of the flexid, there is evidence of a closed fossetid. The third lamina is the most oblique and has a greater width. The fourth lamina is labiolingually shorter than the second one, and it is less oblique.

Neogene neoepiblemid rodents from the Neotropics include two late Miocene genera: †*Neoepiblema* and †*Phoberomys* (Sánchez-Villagra et al. 2003; Horovitz et al. 2006; Kerber et al. 2019), both recorded in the upper Miocene sequence of Urumaco (Carrillo and Sánchez-Villagra 2015). Adult specimens of *Neoepiblema* show the lower premolar and molars with three laminae, while *Phoberomys* spp. has p4 and molars with three and four laminae, respectively (Rasia and Candela 2018; Kerber et al. 2019). However, juvenile specimens (including *Neoepiblema*, Kerber, per. obs.) have five or four laminae composing the teeth (Rasia and Candela 2018; Boivin et al. 2019). Due to the absence of more diagnostic features, the material here reported is assigned to Neoepiblemidae indet. depending on further findings for better taxonomical identification of the San Gregorio neoepiblemid. Vucetich et al. (2010) reported the presence of *Neoepiblema* sp. for this unit (specimen UNEFM-VF-54). However, the specimen is quite fragmented for a confident identification. Here, new evidence of the presence of neoepiblemids confirms the survival of this lineage at the least until the Pliocene. In this sense, San Gregorio Formation neoepiblemids represent the LAD of this rodent clade.

Octodontoidea Waterhouse, 1839

Octodontoidea?

†*Marisela* Vucetich et al., 2010

†*Marisela gregoriana* Vucetich et al., 2010

(Fig. 27G1–G3).

*Locality*: NCC (conglomerate, Fig. 3B).

*Material*: an isolated left M1 or M2 (probably a M2) (AMU-CURS-1221).

*General description, comparisons and remarks*: The specimen AMU-CURS-1221 is a left M1 or M2 (probably a M2) with 4.4 mm in length, tetralophodont, with a sub-rectangular outline, and unilateral hypsodonty. There are four main lophs separated by three labial flexi (Fig. 27G1). The anteroloph is slightly labiomesially oriented in comparison to the other three lophs, which are transversely oriented. The labial tip of this loph is broken off. The posteroloph is transversely shorter than the protoloph and metaloph. The paraflexus and posteroflexus are slightly more penetrating than the mesoflexus. The posteroflexus is in closure process. Lingually, the hypoflexus is oblique, labiomesially oriented, and its tip is opposite to the second loph (protoloph).

The morphology of AMU-CURS-1221 is quite similar to the holotype of *Marisela gregoriana* (UNEFM-VF-55), but more worn, evidencing an ontogenetic older specimen than UNEFM-VF-55, which according to Vucetich et al. (2010) probably represent a young individual. This rodent of enigmatic affinities is endemic to the San Gregorio Formation. It represents a lineage that evolved in the northern portion of the continent, since there are no related fossils in southern South American deposits (Vucetich et al. 2010).

?Caviomorpha indet.

(Fig. 27H–J2).

*Locality*: NCC (conglomerate, Fig. 3B).

*Material*: Three isolated incisor teeth (AMU-CURS-1126, -1155 and -1206) and a small tooth fragment (AMU-CURS-1235).

*General description, comparisons and remarks*: The incisors (Fig. 27H, I) are between 14 and 22 mm in length, elongated, and curved, with the characteristic enamel layer of rodents covering the distal side of the tooth. Only AMU-CURS-1126 and AMU-CURS-1206 preserve the occlusal chisel-like edge. AMU-CURS-1235 is a small tooth fragment (Fig. 27J1, J2), preserving a portion of the occlusal section where one fossetid can be observed. Due to the absence of diagnostic features in incisors rodent teeth and the fragmentary tooth, these specimens cannot be assigned to any of the referred caviomorphs referred from the Vergel Member.

Artiodactyla Owen, 1848

Camelidae Gray, 1821a

Camelidae indet.

(Fig. 28A1–A6).

*Locality*: SGOP (conglomerate Ly1, Fig. 3C).

*Material*: A fragmented right femur (AMU-CURS-1329).

*General description, comparisons and remarks*: AMU-CURS-1329 corresponds to the proximal portion of a right femur. The proximal width (distance from the greater trochanter to the femoral head) measures 64.1 mm. The femoral head is large, with a diameter of 27.6 mm. The fovea of the head is triangular and elongated. The fovea is deep as in the guanaco (*Lama guanicoe*) and *Lama gracilis* (Cartajena et al. 2010) and not a small notch as in *Hemiauchenia* (Meachen 2005). The greater trochanter extends proximally to the same level that the head. The trochanteric fossa is wide, deep, and limited laterally by the intertrochanteric crest. Camelids are recorded in South America since the Pliocene (Gasparini et al. 2017; Carrillo et al. 2018). Although the incomplete preservation of AMU-CURS-1329 does not allow a more precise identification, it provides additional evidence of the early presence of camelids in northern South America during the Pliocene (~ 3.2 Ma) and Early Pleistocene (Carrillo et al. 2018).

Carnivora Bowdich, 1821

Procyonidae Gray, 1825b

†*Chapalmalania* Ameghino, 1908

†*Chapalmalania* sp.

(Fig. 28B1–B4).

*Locality*: SGOP (conglomerate Ly1, Fig. 3C).

*Material*: A partial left hemimandible (AMU-CURS-1327).

*General description, comparisons and remarks*: AMU-CURS-1327 is a partial left hemimandible of ca. 125.6 mm in length that preserves a fragment of the coronoid process and part of the corpus with the first and second lower molars (m1 and m2). The molars are bunodont (Fig. 28B3, B4). The m1 (17.8 mm in length) has the trigonid cuspids organized in a right-angled triangle, with a less-developed paraconid than the metaconid and protoconid. The paraconid is a single cusp, as in *Chapalmalania* cf. †*Ch. ortognatha* (MLP 91-IV-5-1), †*Cyonasua longirostris* (MACN 8290), and †*Cyonasua lutaria* (MLP 34-VI-20–6), and in the living genera *Bassaricyon*, *Bassariscus*, *Potos*, and *Nasuella*, whereas in *Nasua*, *Procyon*, and some *Cyonasua* (e.g., AMU-CURS-224 and AM: 45985) this structure is bifid. It is not possible to observe the presence of the entoconulid and entoconid because of the deterioration of the material. The hypoconulid is present as a posterior cingulum. The m2 (13.16 mm in length) lacks of paraconid, although the anterior region of the molar extends as a broad cingulum. The metaconid and protoconid have the same height. The entoconid is present and developed, as in *Cy. longirostris*, †*Cy. brevirostris*, †*Cy. pascuali*, which makes it different from *Ch. ortognatha*. The hypoconid and the hypoconulid are absent, but instead, there is a ridge, which runs along the postero-lingual region; the posterior projection observed in *Cyonasua* and some living procyonids is not marked. The ventral edge of the corpus is curved as in *Chapalmalania* cf. †*Ch. altaefrontis* (FMNH 14401), whereas in *Cyonasua* is less curved to straight (e.g., *Cy. brevirostris*).

AMU-CURS-1327 is allocated within the genus *Chapalmalania*, although its morphology does not resemble the previously species described from South America. In any case, this specimen forms part of a group of procyonids distinctively larger than other living and extinct members (Additional file 6). Prevosti and Forasiepi (2018) indicated that *Chapalmalania* species were the heavier procyonids in South America, with an estimated body mass between 125 and 181 kg. AMU-CURS-1327 represents the first record of this genus from Venezuela, and the second in northern South America (Forasiepi et al. 2014).

Mammalia indet.

(Fig. 29A1–G2)

*Locality*: NCC (conglomerate, Fig. 3B) and SGOP (conglomerate Ly1, Fig. 3B).

*Material*: a partial right metatarsal (AMU-CURS-1096), a ?caudal vertebra (AMU-CURS-1293), a fragmented tibia (AMU-CURS-1198), a ?right fibula (AMU-CURS-1197), a podial phalange (AMU-CURS-1124), and two ungual phalanges (AMU-CURS-1127).

*General description, comparisons and remarks*: AMU-CURS-1096 is a partial 3rd right metatarsal of 45 mm in length (Fig. 29A1–A4). Part of the body and the distal end are missing. The body and proximal end are straight and smooth (Fig. 29A1, A3, A4), resembling the overall shape of this element in Canoidea (i.e., Procyonidae and Canidae), although its proximal processes are less defined. The proximal surface has a distinctive chevron shape (Fig. 29A2), with its dorsal and ventral medial lobes similar in size and shape. Unfortunately, it is not possible to offer a more detailed taxonomic assignation due to the fragmentary state of the fossil.

AMU-CURS-1293 is an incomplete amphiplatyan ?caudal vertebra of 35 mm in length (Fig. 29B1–B3), from the SGOP locality. AMU-CURS-1198 is a distal portion of a tibia (Fig. 29C1, C2), AMU-CURS-11973 ?right fibula of 50 mm in length (Fig. 29D1, D2), AMU-CURS-1124 a podial phalange (Fig. 29E1–E3), and AMU-CURS-1127 two ungual phalanges (Fig. 29F1–G2), from the NCC locality. Due to their preservation and/or lack of diagnostic characters, these isolated postcranial elements could not be allocated a lower taxonomic level.

## Generic richness and sampling completeness

**Fig. 32:**
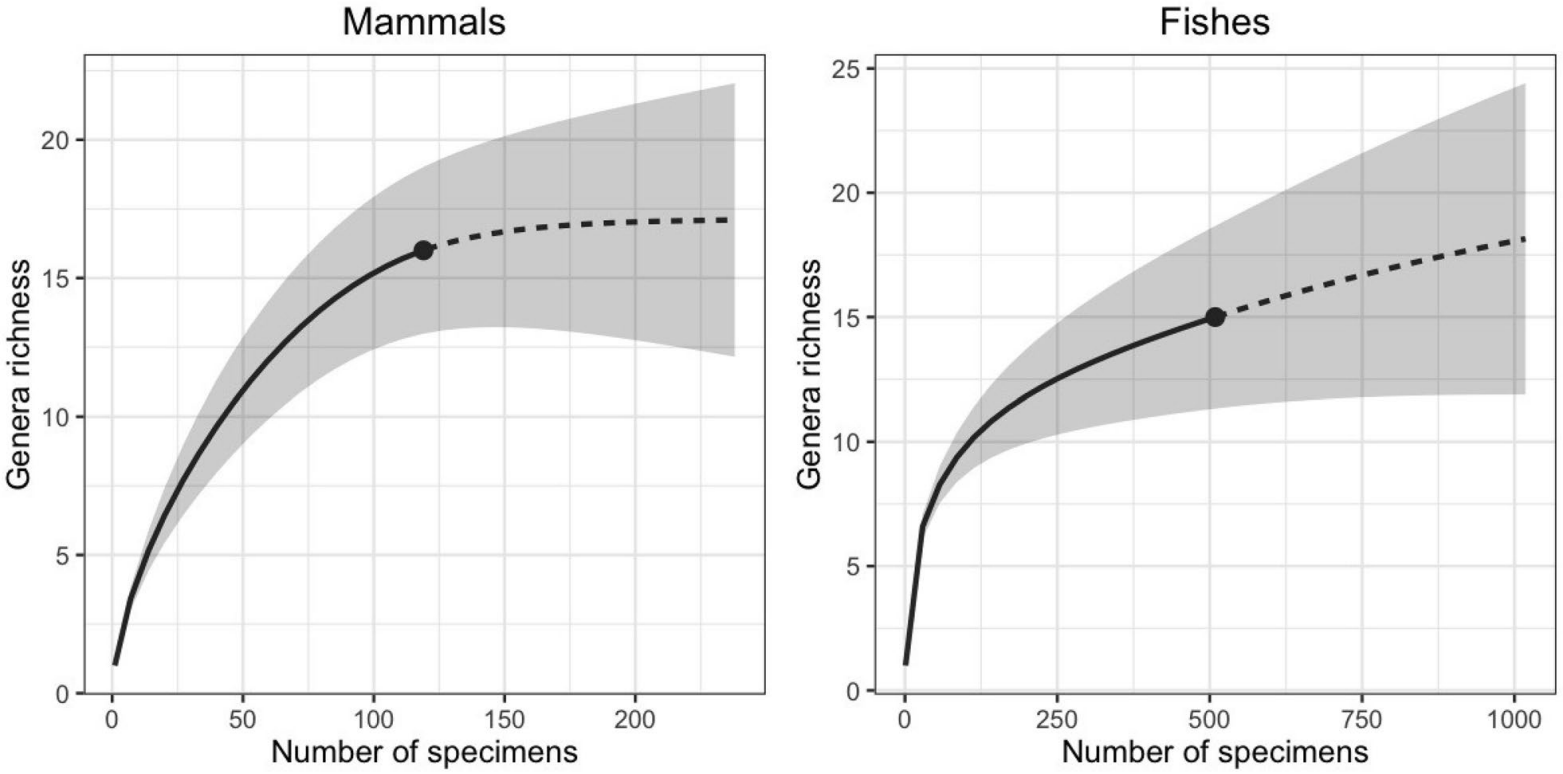
Sample size-based sampling curves for mammals and fishes recorded in Norte Casa Chiguaje. The plots show the rarefaction (solid lines), and extrapolation (dotted lines) sampling and the 95% confidence intervals (shaded areas) for the generic richness of mammals and fishes

A total of 119 mammal specimens and 509 fish specimens were used to compute the rarefaction and extrapolation plots. The extrapolation of generic richness was estimated for double the reference sample size (238 specimens for mammals and 1018 specimens for fishes). For mammals, 16 genera are observed, 11 identified and five unidentified (Table 1). We estimated that 17 genera (95% confidence interval = [13, 21]) would be recorded with a sampling size of 238 specimens (Fig. 32). For fishes, 15 genera are observed, 11 identified and four unidentified (Table 1). We estimated that 18 genera (95% confidence interval = [13, 24]) would be recorded with 1018 specimens (Fig. 32). Overall, the rarefaction and extrapolation plots indicate that the taxonomic sampling at the genus level for both mammals and fishes is representative of the fauna.

**Table 1:** Vertebrate paleodiversity of the San Gregorio Formation

Taxonomy					N° Ts		N° Tt		N° TPr	
					NCC	SGOP	NCC	SGOP	NCC	Refs.
Chondrichthyes	Myliobatiformes	Potamotrygonidae	*Potamotrygon*	sp.	88		1			
		Indet.	Indet.	Indet.	1					
Actinopterygii	Characiformes	Anostomidae	cf. *Megaleporinus*	sp.	2		1			
			*Schizodon*	cf. *S*. *corti*	70		1			
		Erythrinidae	*Hoplias*	sp.	248		1			
		Serrasalmidae	*Mylossoma*	sp.	5		1			
			Indet. (“pacu clade”)	Indet.	42					
	Cichliformes	Cichlidae	Indet.	Indet.	10		1			
	Siluriformes	Ariidae	cf. *Sciades*	sp.	1		1			
		Callichthyidae	Indet.	sp.	6		1			
		Doradidae	cf. *Amblydoras*	sp.	1		1			
			cf. *Scorpiodoras*	sp.	1		1			
			Indet.	Indet.	80		1		3	1
		Heptapteridae	cf. *Pimelodella*	sp.	1		1			
		Loricariidae	Hypostominae	Indet.	12		1			
			Indet.	Indet.	9					
		Pimelodidae	cf. *Platysilurus*	sp.	3		1			
			Indet.	sp.	2		1			
		Indet.	Indet.	Indet.	99					
	Synbranchiformes	Synbranchidae	Synbranchus	sp.	19		1			
	Indet.	Indet.	Indet.	Indet.	195					
Amphibia	Anura	Pipidae	cf. *Pipa*	sp.	1		1			
		Indet.	Indet.	Indet.	20					
Reptilia	Testudines	Testudinidae	*Chelonoidis*	sp.	1		1			
		Chelidae	*Chelus*	sp.	1		1			
		Podocnemididae	Indet.	Indet.	48	3	1	1		
		Indet.	Indet.	Indet.	85					
	Squamata	Teiidae	*Tupinambis* s.l	sp.	1		1			
		(non-snake) Squamata Indet.	Indet.	Indet.	5		2			
	Serpentes	Aniliidae	*Anilius*	*A. scytale*	1		1			
		Boidae	*Corallus*	sp.	1		1			
			*Eunectes*	sp.		1		1		
			Indet.	Indet.	2		1			
		?Boidae or ?Aniliidae	Indet.	Indet.	1		1			
		Colubroidea	Indet.	Indet.	1		1			
		Indet.	Indet.	Indet.	3					
	Crocodylia	Alligatoridae (Caimaninae)	*Caiman*	aff. *C*. *yacare*		1		1		
			Indet.	Indet.	11	1	1			
		Crocodylidae	*Crocodylus*	†*falconensis*			1		1	2
		Indet.	Indet.	Indet.	571	12		1		
Mammalia	Didelphimorphia	Didelphidae	cf. *Didelphis*	sp.	1		1			
	Xenarthra (Pilosa)	†Megatheriidae	cf. †*Proeremotherium*	sp.	1	1	1	1	1	3
		†Mylodontidae	Indet.	Indet.	1		1			
	Xenarthra (Cingulata)	Dasypodidae	†*Pliodasypus*	*vergelianus*			1		3	4
		†Glyptodontidae	aff. †*Boreostemma*	sp.	2		1		14	5, 6
		†Pampatheriidae	aff. †*Holmesina*	*floridanus*	2	2	1	1	?	7
			aff. †*Plaina*	sp.	1		1			
		Indet.	Indet.	Indet.		1				
	†Litopterna	†Proterotheriidae	Indet.	Indet.	2		1			
	†Notoungulata	†Toxodontidae	†*Falcontoxodon*	sp.	33		1		38	8
			Indet.	Indet.		3		1		
		†Interatheriidae (Typotheria)	Indet.	Indet.	1		1			
		†Typotheria inc. Sed	Indet.	Indet.	1		1			
	Rodentia	Cricetidae^a^	Indet.	Indet.	8^a^		1			
		Hydrochoeridae	†*Cardiatherium*	sp.			1		1	7
			cf. †*Caviodon*	sp.			1		1	7
			†?*Hydrochoeropsis*	*wayuu*	2		1			
			Indet.	Indet.	3					
		†Neoepiblemidae	†*Neoepiblema*	sp.			1		1	7
			Indet.	Indet.	1		1			
		Octodontoidea?	†*Marisela*	*gregoriana*	1		1		2	7
		Caviomorpha	Indet.	Indet.	4					
	Artiodactyla	Camelidae	Indet.	Indet.		1		1		
	Carnivora	Procyonidae	†*Cyonasua*	sp.			1		1	9
			†*Chapalmalania*	sp.		1		1		
	Indet.	Indet.	Indet.	Indet.	7					

Localities: Norte Casa Chiguaje (NCC) and San Gregorio Oeste del Pueblo (SGOP). Total number of specimens for locality (N° Ts). Total estimated taxa per locality (N° Tt). Total number of specimens referred in previous publications (N° TPr) and their references (Refs.): (1) Aguilera et al. (2013); (2) Scheyer et al. (2013); (3) Carlini et al. (2018); (4) Castro et al. (2014); (5) Zurita et al. (2011); (6) Carlini et al. (2008c); (7) Vucetich et al. (2010); (8) Carrillo et al. (2018); (9) Forasiepi et al. (2014)

^a^Personal communication (Dr. U. Pardiñas)

## Paleobotanical remains

**Fig. 33:**
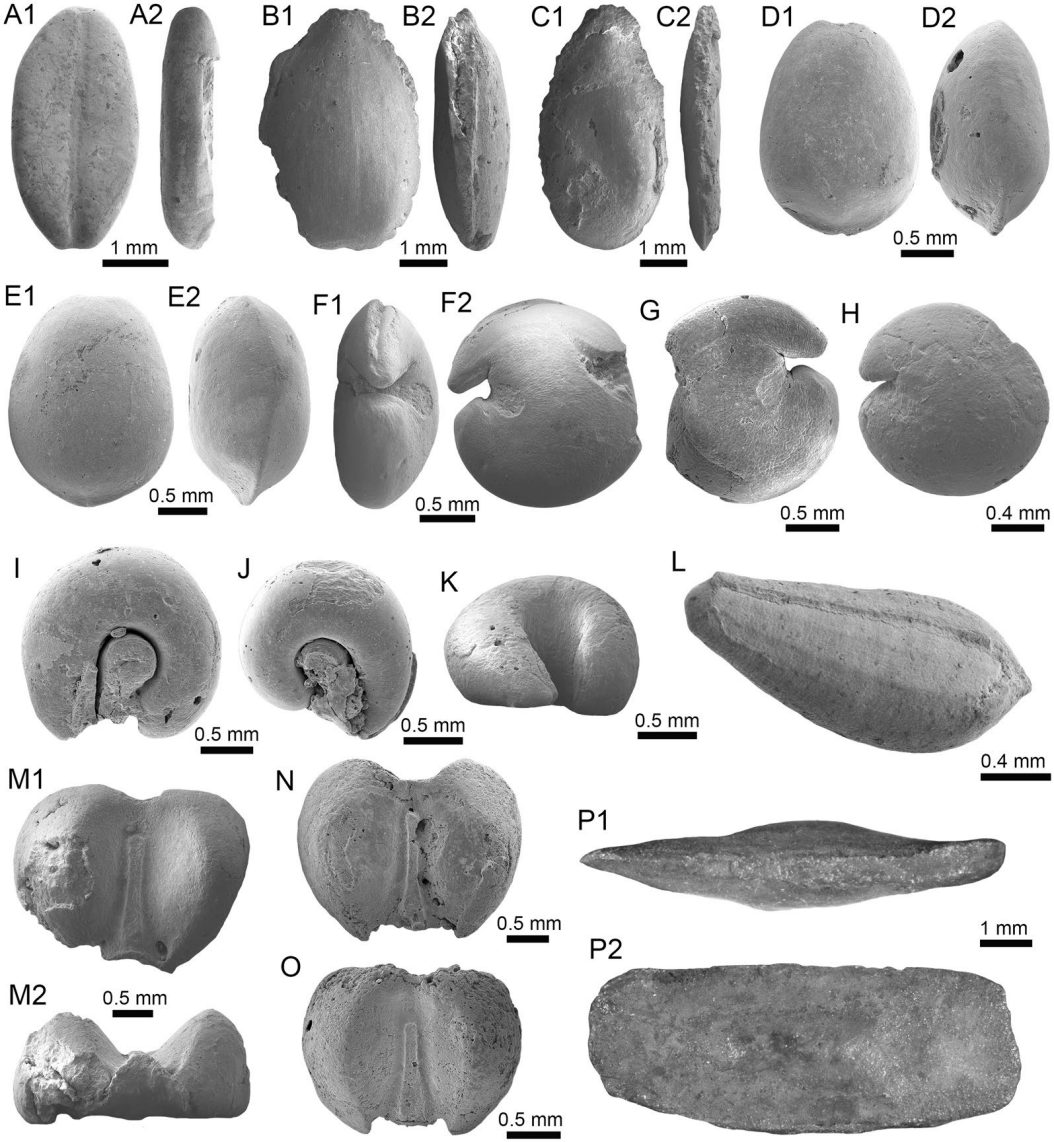
Internal seed casts from the Norte Casa Chiguaje locality (Vergel Member). **A1**, **A2** Morphotype 1 (AMU-PB-02), Poaceae *aff*. Chloroideae. **B1**–**C2** Morphotype 2 (AMU-PB-03), Cucurbitaceae. **D1**–**E2** Morphotype 3 (AMU-PB-04), of indeterminate affinities. **F1**–**H** Morphotype 4 (AMU-PB-05), Amaranthaceae. **I**–**K** Morphotype 5 (AMU-PB-06), *aff*. Cleomaceae. **L** Morphotype 6 (AMU-PB-07), Asteraceae. **M1**–**O** Morphotype 7 (AMU-PB-09), *aff*. Vitaceae. **P1**–**P2** Morphotype 8 (AMU-PB-10) of indeterminate affinities

From the NCC locality, 140 fossil seeds and fruits were collected from the screen-washed sediments, and were grouped into eight morphotypes based on their distinct morphology (Fig. 33). These fossil remains were preserved as limestone casts of the seed/fruit interior, and in many cases, they carry imprints of the internal cellular pattern of the seed coat. Despite the lack of internal anatomical preservation, distinct features and the overall morphology of six of these are sufficient to provide familial affiliations. Among these fossil seeds and fruits, we recognize Poaceae, identified from a caryopsis cast showing a dorsally placed embryo and a flat ventral face and needle-shaped hilum (Morphotype 1, Fig. 33A1, A2) that resembles various taxa of Chloroideae (Liu et al. 2005). Morphotype 2 (Fig. 33B1–C2) includes distinctly compressed, pyriform seeds with impressions of polygonal, elongated cells of the internal seed coat that are identified as Cucurbitaceae (Heneidak and Khalik 2015; Schaefer and Renner 2010). The rounded, subglobose seeds grouped into Morphotype 4 (Fig. 33F1–H) are identified as Amaranthaceae based on a distinct hilar notch and peripheral embryo that surrounds a well-differentiated perisperm, as seen on species of Chenopodioideae (Kühn et al. 1993; Townsend 1993). Asteraceae is also recognized within this assemblage based on an ovoidal, slightly curved and striated cypsela (Morphotype 6, Fig. 33L) with a distinct apical “neck” showing the pappus insertion site (Anderberg et al. 2007; Ghimire et al. 2018). Two seed cast types with limited morphological preservation have tentative affinities to Cleomaceae and Vitaceae. Morphotype 5 (Fig. 33I–K) is interpreted as the cast of a strongly curved, reniform, or horseshoe-shaped seed with an incurved embryo and is identified as *aff*. Cleomaceae, whose seeds have strongly incurved embryos and a deep invagination of the testa (Iltis et al. 2011). Morphotype 7 (Fig. 33M1–O) is identified as *aff*. Vitaceae based on a distinctly elongated scar that resembles the chalazal knot seen in seeds of this family (Chen and Manchester 2011). Morphotype 3 (Fig. 33D1–E2) and morphotype 8 (Fig. 33P1, P2) so far could not be identified.

## Discussion

Neogene continental vertebrate faunas of the Urumaco region come from a successive geological sequence that includes the Socorro, Urumaco, Codore, and San Gregorio formations. Their lithological, taphonomic, and paleoenvironmental features are different from each other (see Quiroz and Jaramillo 2010). The new continental vertebrate fauna described here from the Vergel and Cocuiza members of the San Gregorio Formation provide novel data about the late Neogene diversity in northernmost South America, and the geographical and temporal range of several lineages during the transitional stage that preceded the major climatic shift of the Quaternary.

## Age of the San Gregorio Formation

Dating of the San Gregorio Formation has been a long-standing challenge. Several studies estimated its age by stratigraphic position or correlation with other formations (González de Juana et al. 1980; Audemard 2001). Only two previous studies have provided biostratigraphic data: an unpublished MS thesis (Rey 1990) that reported several molluscan taxa of little biostratigraphic value (e.g., *Crassostrea*, *Argopecten*, *Amusium*, *Placuanomia*, *Pecten*, *Anomia*, *Ostrea*, *Anadara*, *Dosinia*, *Chione*, *Solecurtus*, *Macom*a, *Trachycardium*, *Florimetis*, *Conus*, *Epitonium* and *Turritella*), and a palynological study reporting †*Stephanocolpites evansi* in the Cocuiza Member (Hambalek et al. 1994), which indicates that this member cannot be older than late Miocene (Lorente 1986). Additionally, a late Pliocene age has been assigned for the Vergel Member based on its mammalian associations (see Vucetich et al. 2010). A Pleistocene age is suggested for the San Gregorio Formation, based on a compilation derived from multiple foraminifera, nannoplankton, and magnetostratigraphic studies (Carrillo et al. 2018, Fig. 28).

*Vergel Member*: The last appearance datum (LAD) of †*Bombacacidites nacimientoensis* (senior synonym of *B*. *bellus* of Lorente 1986), †*Retitrescolpites*? *irregularis*, and †*Rhoipites guianensis* at the top of the Vergel Member indicates the top of zone VIII-c (interval zone of Echitricolporites–Alnipollenites), as ~ 1.8 Ma (Lorente 1986). It is important to note that Lorente’s definition of the Pliocene–Pleistocene boundary followed the pre-2012 definition that had the boundary at the base of the Calabrian (1.81 Ma), while currently it is placed at the base of the Gelasian (2.59 Ma) (Hilgen et al. 2012). The presence of the pampatheriid *Plaina* in the NCC locality, approximately 180 m below the Vergel/Cocuiza contact (Fig. 3A), suggests an age no younger than Pliocene (Fig. 30) as *Plaina* has a biochron that spans from the late Miocene to Pliocene (Gois 2013). Therefore, in the NCH section at least the upper ~ 50 m of the Vergel formation (that is 230 m thick in the NCH section; Fig. 3A, Additional file 1) would correspond to the Early Pleistocene, within the upper zone VIII-c (Fig. 30), while the Pliocene–Pleistocene boundary could be somewhere between stratigraphic meters 130 and 180 of the Vergel Member (Fig. 30).

*Cocuiza Member*: The ^86^Sr/^88^Sr dating of two samples rendered ages of 1.38 Ma (1.33 to 1.44, ^87^Sr/^86^Sr = 0.709112) and 1.59 Ma (1.54 to 1.65 Ma, 87Sr/86Sr = 0.709100) (Additional file 5), indicating a Calabrian age for the Cocuiza Member (Fig. 30). This age is also supported by the record of nannoplankton. The stratigraphic range of †*Pseudoemiliania lacunosa* is restricted to biozones NN15-NN19 of Martini (1971), which are dated as Pliocene–Pleistocene (Hilgen et al. 2012). The extinction of this species was astronomically calibrated in the eastern equatorial Atlantic, yielding an age of 0.4 Ma (Shackleton and Crowhurst 1997; Backman et al. 2012). The occurrence of †*Helicosphaera sellii* and *Gephyrocapsa* spp. (Additional file 4) also supports a Pleistocene age. The foraminifera, in contrast, indicate an older age, as *Ammonia beccarii*, *Elphidium poeyanum*, and *Melonis barleeanum* are common in the upper Miocene–Pliocene *Ammonia becarii* zone in northern South America (Duque 1975; Duque Caro et al. 1996). *Globoturborotalita* cf. *woodii* and *Globoturborotalita* cf. *rubescens* are found in biozones N19–N21 [Zanclean and Gelasian after Kennett & Srinivasan (Kennett and Stott 1991)]. However, the foraminifera assemblage is poorly preserved and it has a large number of reworked taxa. Therefore, our confidence in the age derived on foraminifera is much lower compared to both the Strontium and nannoplankton dating.

The SGOP locality section “S2” of the Cocuiza Member (Figs. 1A and 3C) is characterized by the presence of conglomeratic layers (Fig. 2G, H) that could correspond with those terrigenous sediments reported for the unit by Rey (1990) and Hambalek et al. (1994). It was not possible to correlate the SGOP locality to either the NCH (Figs. 1A and 3A; Additional file 1) or SGRS (Fig. 1A; Additional file 2) sections. Although a more detailed stratigraphic section would be necessary in the future in order to correlate SGOP locality with NCH or SGRS sections, our field observations tentatively suggest that the SGOP locality may belong to the middle section of the Cocuiza Member, which was deposited during the Early Pleistocene (Fig. 30).

## Paleodiversity and biostratigraphic affinities

Terrestrial and freshwater vertebrates from the San Gregorio Formation had been reported exclusively from the Vergel Member (Table 1). Previous reports include indeterminate catfishes (Aguilera et al. 2013), crocodylians (Scheyer et al. 2013), terrestrial sloths, glyptodontids, pampatheriids, dasypodid armadillos (Carlini et al. 2008c, 2018; Carlini and Zurita 2010; Vucetich et al. 2010; Zurita et al. 2011; Castro et al. 2014), notoungulates (Carrillo et al. 2018), a procyonid (Forasiepi et al. 2014), and caviomorph rodents (Vucetich et al. 2010). The exception is †*Crocodylus falconensis* (Scheyer et al. 2013), whose fossiliferous locality is located a few meters above the NCC locality in the NCH stratigraphic section (Fig. 3A). Due to the geographical proximity between *C. falconensis* and NCC localities, Scheyer et al. (2013) recognized the former locality as within the NCC locality area.

Forty-nine aquatic and terrestrial taxa are here reported for the NCC locality (Table 1), where fishes and mammals are the most diverse and abundant groups (Fig. 31). To our knowledge, no other continental late Pliocene deposit in northern South America has shown such a diverse continental aquatic/terrestrial taxonomic richness. Late Pliocene units with aquatic and terrestrial faunas comparable to those of the NCC locality include the Ware Formation in the Cocinetas Basin (Guajira Peninsula, Colombia) in northern South America. Although less diverse, the Ware Formation is characterized by at least eight species of fishes, three reptile taxa, one bird, and 13 mammalian taxa (Aguilera et al. 2013; Moreno et al. 2015; Moreno-Bernal et al. 2016; Carrillo et al. 2018). Hendy et al. (2015) reported a late Pliocene age (mean age of 3.2 Ma) for the Ware Formation, based on ^87^Sr/^86^Sr ratios of the shell bed at the top of the unit. The mammalian assemblage of the Ware Formation is characterized by a diversity of herbivores, including sloths (Amson et al. 2016), cingulates, caviomorph rodents (Pérez et al. 2017), toxodontids, and a proterotheriid. It also includes a procyonid (Forasiepi et al. 2014) and a camelid, which are immigrants from North America (Carrillo et al. 2018). The mammalian assemblage of the Ware Formation has a higher richness of terrestrial sloths (with at least five different taxa; see Amson et al. 2016) than the assemblage from NCC (Table 1). In contrast, the NCC locality is more taxon-rich than the Ware assemblage in other mammalian groups, such as cingulates, meridiungulates, and rodents. However, this difference in diversity between both units could be related to taphonomic or sampling biases.

The Ware and San Gregorio formations outcrops are geographically close, less than 140 km in a northernmost portion of South America and probably were part of the same biogeographic province during the Pliocene–Pleistocene. The presence of ?*Hydrochoeropsis wayuu* in both Ware Formation (Pérez et al. 2017) and Vergel Member supports the biostratigraphic correlation between these two geological units, as suggested by Moreno et al. (2015). The mammalian assemblages in the Ware Formation and at the Vergel Member are characterized by a predominance of South American native taxa (Carrillo et al. 2018) (Table 1). Immigrants from North America are scarce in both units in spite of their age and proximity to the Isthmus of Panama (Carrillo et al. 2018). Until now, the North American immigrants in the Ware assemblage include the procyonid *Chapalmalania* (Forasiepi et al. 2014), and one of the oldest well-dated camelids in South America (Carrillo et al. 2018). The San Gregorio Formation also includes *Chapalmalania* and an indeterminate camelid from the SGOP locality, and *Cyonasua* (Forasiepi et al. 2014) and some Cricetidae rodents with boreal affinities (Ulyses Pardiñas, personal communication, March 2020, which are currently under study and reported in Table 1) from NCC locality. The South American cricetids possibly differentiated from other lineages by the middle/late Miocene (see Parada et al. 2013; Leite et al. 2014). Nevertheless, the Neogene fossil record was restricted to fossiliferous localities in Argentina, with a putative record in late Miocene strata (Nasif et al. 2009), and confident records from Pliocene deposits (see Reig 1978; Pardiñas and Tonni 2014; Pardiñas et al. 2002; Verzi and Montalvo 2008; Prevosti and Pardiñas 2009).

Carrillo et al. (2018) placed the Ware Formation and its fauna within the first migration pulse of the Great American Biotic Interchange (GABI, Woodburne 2010), and the San Gregorio Formation (without any differentiation of members) might have overlapped with the second and third migratory pulse, named GABI 2 and GABI 3 (Carrillo et al. 2018, fig. 1). With the late Pliocene age proposed here for most of the Vergel Member (Figs. 3A, 30), the NCC assemblage would have to be reinterpreted within GABI 1 (see Carrillo et al. 2018, fig. 1).

Sampling in the geographic location of the Guajira Peninsula and Falcón region is critical to improving the understanding of the first GABI phases and the timing of the appearance of immigrants from North America into South America. For example, NCC locality is the only northern Neogene unit in which fossils of caviomorphs and cricetids (under study) are found in the same levels. Since about 41 million years, caviomorphs were the only clade of rodents in South America (Antoine et al 2012), generating a wide diversification of disparate lineages in morphology, body size, and ecology. After the latest Miocene/early Pliocene several groups of caviomorphs disappeared (e.g., large dinomyids and neoepiblemids) (Vucetich et al. 2010, 2015; Kerber et al. 2020), while cricetids arrived from North America. The co-occurrence of caviomorphs, including the last neoepiblemids, and cricetids in the NCC fauna, offer an opportunity to better understand the dispersal of cricetids in the tropics of South America as well as the extinction of some caviomorph lineages.

The current and precise temporal allocation of NCC and SGOP localities of the San Gregorio Formation agree with the hypothesis proposed by Carlini et al. (2006a, b, 2008b), and Carlini and Zurita (2010), about possible migration times to Central America. In addition, the San Gregorio Xenarthra show anatomical features that are plesiomorphic if compared to those of the late Pliocene–Early Pleistocene taxa recorded in North America and Mexico.

Another fossil-rich (tar pit) locality called El Breal de Orocual “ORS16,” in Monagas State, northeast of Venezuela, yields an assemblage of terrestrial taxa that exceeds 30 spp. (mostly mammals) (Rincón et al. 2009; Solórzano et al. 2015). The Orocual fauna is tentatively assigned to the late Pliocene–Pleistocene, based on the biochron of †*Smilodon gracilis*, †*Pachyarmatherium leiseyi*, and the rodents †*Phugatherium* sp., (Vucetich et al. 2012 considers †*Chapalmatherium* as synonymous of *Phugatherium*), †*Neocavia* sp., and a tetrastylines (Rincón et al. 2009; Solórzano et al. 2015; Czaplewski and Rincón 2020). Additional dating is necessary to confirm the age of the Orocual assemblage because the biochronology of several mammalian taxa in the tropics is poorly known and it may differ from that of temperate regions. For example, *Chapalmalania* is recorded is Buenos Aires (late Pliocene) and Catamarca (?early Pliocene) in Argentina, and the Guajira in Colombia (late Pliocene) (Ameghino 1908; Reguero and Candela 2011; Forasiepi et al. 2014; Prevosti and Forasiepi 2018). Our field observations suggest that the SGOP locality may belong to the middle section of the Cocuiza Member (and therefore have a Calabrian age, Fig. 30). Therefore, the *Chapalmalania* record in the SGOP would represent the youngest known for the taxon, expanding the biochron of this genus into the Pleistocene (Calabrian).

A wide range of fossil fishes, reptiles, and mammals from the Urumaco sequence (Lundberg et al. 2010; Sánchez-Villagra et al. 2010; Aguilera et al. 2013; Scheyer et al. 2013; Aguirre-Fernández et al. 2017a, b) have been used as unequivocal evidence to support a system with hydrographic connections between western Amazonia and the Proto-Caribbean Sea during the Miocene (e.g., Díaz de Gamero 1996; Hoorn et al. 2010). However, by the late Miocene to early Pliocene, extreme environmental changes and a faunal turnover took place in the region (Sánchez-Villagra et al. 2010; Scheyer et al. 2013). This process has been linked to a major hydrographic restructuring as a consequence of the northern Andes uplift (Mora et al. 2010; Albert et al. 2018), and may have led to the complete isolation of northern peripheral drainages from those of western Amazonia triggering a direct impact (e.g., extinction/extirpation) in fishes, crocodylians, turtles, and some putatively semiaquatic mammals (e.g., rodents) (Lundberg et al. 1998, 2010; Sánchez-Villagra et al. 2010; Scheyer et al. 2013; Cadena et al. 2020). These major changes in the dynamics of the sedimentary and environmental conditions of the Falcón region are documented during the deposition of the Codore Formation (Quiroz and Jaramillo 2010).

Vucetich et al. (2010) proposed the Vergel Member as a “reservoir” for rodent taxa that had gone extinct in southern South America. According to Vucetich et al. (2010), this survival of taxa would be related to the persistence of fluvial environments under warm conditions. Although Vucetich et al. (2010) reported the presence of *Neoepiblema* sp. for the NCC locality, that specimen (UNEFM-VF-54) was in too poor condition for a reliable identification. The new evidence presented here confirms the survival of this lineage at least until the late Pliocene. However, a “reservoir” hypothesis during the late Pliocene in the Falcón region must be viewed with caution, as there are no other Pliocene–Early Pleistocene fossiliferous localities in the region that can validate or discard this attribution. Additionally, the occurrence of small notoungulates (Interatheriidae and Typotheria inc. sed) in the Pliocene of the Vergel Member (Table 1) represents the first record in the region and suggests the existence of ghost lineages inhabiting tropical areas since the Paleogene.

Thorny catfishes, such as cf. *Amblydoras* and cf. *Scorpiodoras*, have living representative species inhabiting exclusively the cis-Andean (Eastern-slope) rivers from the Orinoco and Amazon basins (Sousa and Birindelli 2011; van der Sleen and Albert 2018). Other freshwater taxa from the NCC locality, such as *Potamotrygon* sp., cf. *Megaleporinus* sp., *Schizodon* cf*. S*. *corti*, *Mylossoma* sp., and cf. *Platysilurus* sp., were extirpated from the Falcón region. Living representatives of the above-mentioned fishes still inhabit both the cis-Andean (van der Sleen and Albert 2018) and trans-Andean basins, being restricted in the later exclusively to the Magdalena and Lake Maracaibo basins (Pérez and Taphorn 1993; Rodríguez-Olarte et al. 2009). The presence of the above-mentioned stingray, thorny catfishes, and characiform taxa in NCC locality suggests fluvial conditions during the Pliocene time that contrast with those prevailing today in the Falcón region. Aguilera et al. (2013), based on paleoichthyological evidence from the Urumaco and Guajira Peninsula regions, suggested a possible last connection between the Orinoco/Amazon basins and those of the Caribbean region for the Pliocene. However, new geological models support a complete hydrographic isolation between western Amazonia and the Caribbean basins during the Pliocene (Albert et al. 2018).

Extant matamata turtles are represented by two species inhabiting exclusively the Orinoco and Amazon basins (Vargas-Ramírez et al. 2020), whereas that the taxonomic status of some records from the Lake Maracaibo basin is unresolved (Trebbau and Pritchard 2016). The fossil record of matamatas is well known from the late Miocene of Urumaco and the Cocinetas basin in Colombia (Sánchez-Villagra et al. 2010; Cadena and Jaramillo 2015). The presence of this taxon can now be extended into the Pliocene of Falcón.

Only a limited number of isolated snake vertebrae have been recovered from the San Gregorio Formation. Nevertheless, these vertebrae provide a glimpse into the ophidian fauna and its evolution in the area. The NCC snake assemblage comprises at least four different species, while the younger Cocuiza Member has yielded only a single vertebra. Among the NCC remains, the presence of *Anilius scytale* is notable, considering that this cryptic taxon occurs in the extant herpetofauna of northern South America, including parts of Venezuela, but is currently absent from Falcón State and other Caribbean basins (Mijares-Urrutia and Arends 2000). This single NCC record represents, to our knowledge, the sole known fossil occurrence of *Anilius scytale* and can thus offer a fossil calibration point for this species into the late Pliocene. Furthermore, the fact that this Pliocene record lies outside the current geographic range of the species implies a post-Pliocene local extirpation. Boidae in NCC are represented by at least two forms, one of which is attributed to the extant genus *Corallus*, which is widespread in the Neotropics (Wallach et al. 2014; Reynolds and Henderson 2018), including the Falcón State (Mijares-Urrutia and Arends 2000). *Corallus* represents a relatively ancient lineage, already recorded since the early Cenozoic, as attested by the extinct species †*Corallus priscus* Rage, 2001, from the early Eocene of Itaborai, Brazil (Rage 2001; see Smith and Georgalis in press). The NCC vertebra (AMU-CURS-1158) that bears some resemblance with *Colombophis* is so far inadequate for determining whether this Neogene Neotropic genus was indeed present in the area or if the vertebra belongs to some other anilioid or boid form. As for Colubroides, this is the dominant and most speciose lineage of snakes in extant herpetofaunas, not only in the Neotropics but also all over the globe (Zaher et al. 2009). As such, the single, fragmentary vertebra from NCC that we assigned to Colubroides cannot offer any more precise assignment to any of the numerous taxa of that lineage that currently inhabit that area. On the other hand, the single vertebra from the Early Pleistocene of the Cocuiza Member evidences the presence of the giant snake anaconda, i.e., *Eunectes*. This genus is currently absent from the area, and therefore, its herein documentation in the early Quaternary implies a relatively recent extirpation from the region. Based on this limited fossil anaconda material, it is not possible to determine whether this new Venezuelan taxon is phylogenetically closer to the older taxon *Eunectes stirtoni* from the Miocene of Colombia (Hoffstetter and Rage 1977) or to the extant *Eunectes* spp. that currently inhabit the Orinoco and Amazon basins.

Given the fragmentary nature of most crocodylian remains from the San Gregorio Formation, there is little information so far about the connectivity of these fossils and their faunal composition in comparison to the modern fauna of South America. In the case of the cranial remains identified as *Caiman* aff. *C. yacare* and of the well-preserved skull of the *Crocodylus falconensis* (Scheyer et al. 2013), however, some broader implications are warranted. The extant *Caiman yacare*, the southern or Paraguayan spectacled caiman, has a distribution restricted to the southern hemisphere, with a northernmost extension into Bolivia and central/southwestern Brazil (Grigg and Kirschner 2015). The identification of AMU-CURS-1328 as *Caiman* aff. *C. yacare* from the Cocuiza Member tentatively implies that the distribution of the southern spectacled caiman could have had a more extensive northward distribution up to Caribbean, even until the Early Pleistocene (Calabrian). A fossil representative of the northern spectacled caiman, *Caiman crocodilus*, was recently reported from the Pliocene–Pleistocene El Breal de Orocual tar pit in northeastern Venezuela (Cidade et al. 2019b). The overlap of the northern with the southern species of spectacled caimans might thus have been more extensive in the recent past in comparison to today’s restricted overlap zone along the northern border of Bolivia with Brazil (e.g., Andrade et al. 2020).

Crocodylidae are represented in South America today only by the American crocodile, *Crocodylus acutus*, ranging from Central America to Colombia and throughout the Caribbean, and the critically endangered Orinoco crocodile, *Crocodylus intermedius*, restricted to Colombia and Venezuela (Grigg and Kirschner 2015). Scheyer et al. (2013) recovered *C. falconensis* from the Pliocene Vergel Member as sister taxon to all New world crocodiles, including the two extant crocodile species. Together with lower jaw remains identified as *Crocodylus* sp. from the Pliocene Ware Formation of Colombia (Moreno-Bernal et al. 2016), *C. falconensis* is still considered the oldest well-known record of true crocodylids in South America (Cidade et al. 2019a). A recent phylogenetic analysis refined the position of *Crocodylus falconensis* to within the extant New World crocodiles and *Crocodylus intermedius* being the sister taxon to the remaining species (Delfino et al. 2020).

## Paleoenvironments

*Vergel Member*: According to Hambalek et al. (1994), fluvial environments prevailed in the Urumaco region during the deposition of the Vergel Member with depositional environments characterized by alluvial plains and braided rivers (Rey 1990). These conditions were later replaced by a marine incursion that is represented by the deposition of the overlying Cocuiza Member (Hambalek et al. 1994). Foraminifera with low-salinity tolerance indicate the presence of estuaries nearby (Smith et al. 2010).

The conglomeratic layer that bears most of the fossils from NCC Locality (Fig. 3B) is evidence of transportation and accumulation in river channels (Quiroz and Jaramillo 2010). The paleontological evidence, especially the aquatic faunal composition of the NCC assemblage (Table 1), supports this idea and is an unequivocal element supporting the presence of permanent watercourses. The ichthyofauna from NCC assemblage is characterized by a typical tropical-freshwater composition. The habitat preferences of extant taxa related with fossil characiforms (e.g., cf. *Megaleporinus*, *Schizodon* cf. *S*. *corti*, and *Mylossoma* sp.) and Siluriformes (e.g., cf. *Amblydoras* sp., cf. *Scorpiodoras* sp., cf. *Pimelodella* sp., cf. *Platysilurus* sp., and indeterminate pimeloids and loricariids) suggest flowing waters in a well-oxygenated environment (Additional file 7). The stingray *Potamotrygon* sp. is abundant in the fossil assemblage (Table 1) and although living species are found even in lakes and still waters, they are commonly associated with flowing rivers and particularly on sandy substrates (see Lasso et al. 2014). Although the fossil ichthyofauna from NCC assemblage supports the presence of flowing waters, other environments, such as swampy, ponds, and marginal areas associated with a flooding process, may have also existed. For example, the extant relatives of the armored Callichthyidae catfishes and the freshwater eel *Synbranchus* sp. (Table 1 and Additional file 7) are able to survive also in warm, anoxic, and temporary waters due to their aerial respiration capacity (Lundberg et al. 2010; van der Sleen and Albert 2018). Most of the fossil fish taxa from NCC assemblage have living representatives that exclusively inhabit freshwater environments. However, the presence of euryhaline species is feasible, especially due to the probable proximity to the marine area during the deposition of the Vergel Member (Hambalek et al. 1994; Smith et al. 2010). A clear example could be the presence of the Ariidae catfish cf. *Sciades*, a genus whose living species have the ability to migrate upstream (Marceniuk and Menezes 2007).

The presence of a pipid amphibian is also indicative of freshwater environments, as Pipidae anurans are strictly linked to aquatic conditions and rarely use terrestrial environments (Wells 2007). Crocodylian (teeth, osteoderms, and vertebrae) and podocnemidid turtle remains (shell fragments) are among the most abundant fossils of aquatic vertebrates in the NCC assemblage. The habitat preferences of their extant relatives are freshwater lakes, marshes, swamps, mangroves, and flowing waters (Additional file 7), supporting also the evidence of freshwater environments, although the presence of *Crocodylus falconensis* might imply the existence of estuarine settings nearby. Members of *Crocodylus* live in estuarine or mixed environments (in addition to rivers). Likewise, the environments in which both types of crocodiles (a caiman and a true crocodile) occur today in America are few. For example, the Orinoco or Magdalena River deltas are potentially habitats for both species and could give an idea of the characteristics of the Falcón area in the Pliocene–Pleistocene.

**Fig. 34:**
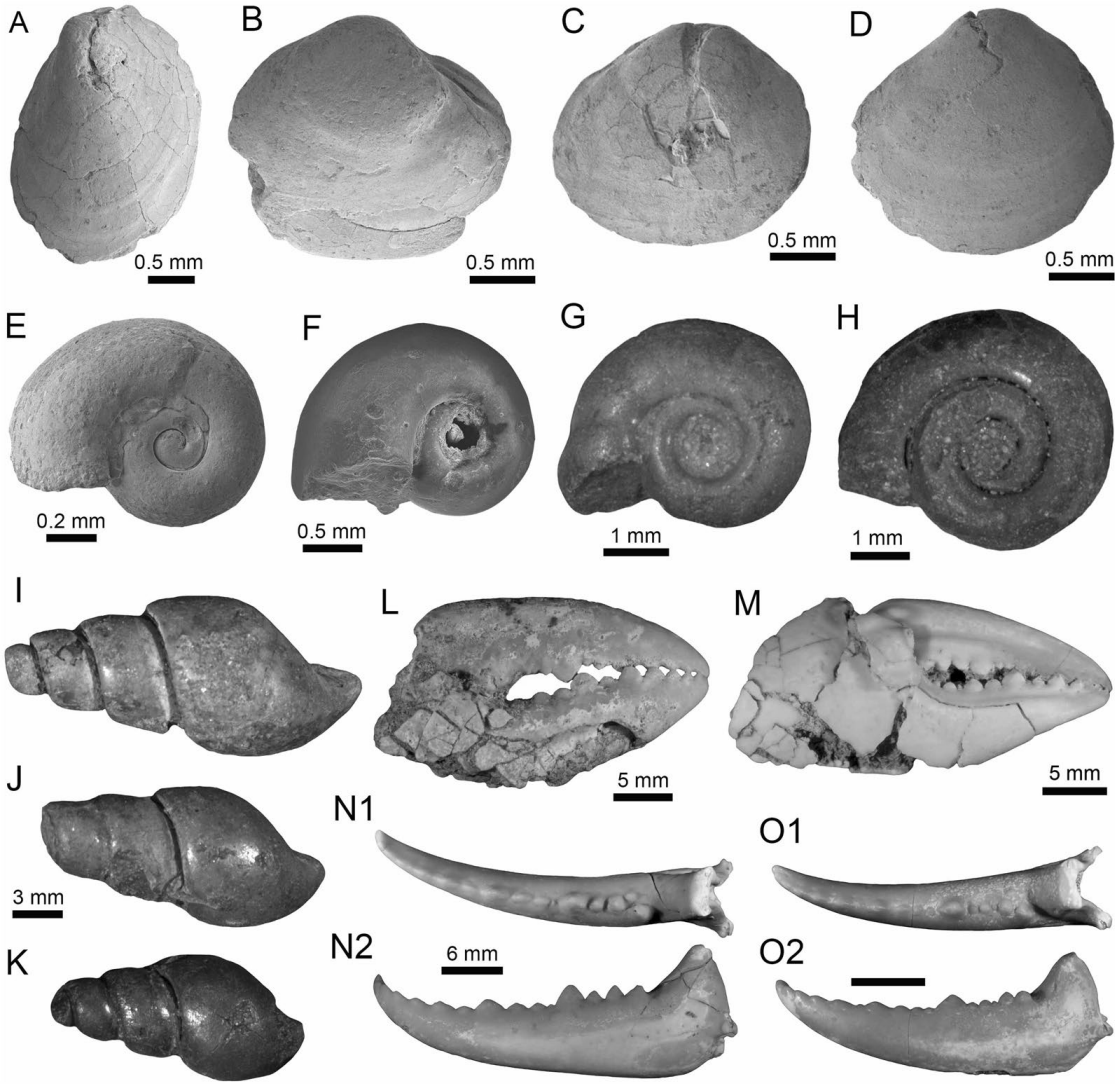
Freshwater molluscs and crustaceans from the Vergel Member. **A**–**D** Internal molds of indeterminate bivalves (AMU-PI-81). **E**–**H** Internal molds of gastropods (AMU-PI-82) presumably related to Planorbidae. **J**, **K** Internal molds of indeterminate gastropods (AMU-PI-83). **L**–**O2** Right chelae (**L**, **M**) and moveable fingers (dactyl: **N1**–**O2**) of indeterminate freshwater crabs Trichodactylidae (AMU-PI-15)

The matamata turtle (*Chelus*) is another example that supports the existence of freshwater environments. The extant species of *Chelus* are associated mainly with slow-moving waters, swamps, and marshes (Trebbau and Pritchard 2016). In addition to the aquatic vertebrate assemblage, the NCC locality yielded abundant remains of freshwater mollusks (Fig. 34A–K) and Trichodactylidae crabs (Fig. 34L–O2) (e.g., Rodríguez 1997). Although mollusks were preserved only by internal molds of bivalves and gastropods, some of the latter could be tentatively allocated to Planorbidae (Fig. 34E–H), which are characteristics of fresh water environments, including slow-moving rivers (Hanley 1980). As for the NCC snakes, *Anilius scytale* is a strictly fossorial form that requires humid soil and proximity to bodies of water (Martins and Oliveira 1999). The boid *Corallus*, on the other hand, is an efficient arboreal dweller and its occurrence indicates the presence of a forested environment (Martins and Oliveira 1999).

Neoepiblemids and hydrochoerines rodents show a fossil record associated with strata formed by permanent bodies of water, such as rivers, lakes, and marshes (Vucetich et al. 2010; Kerber et al. 2017). Their presence in the NCC assemblage is in accordance with wet and humid paleoenvironmental conditions. The presence of terrestrial sloths, glyptodontids, pampatheriids, and dasypodids in the NCC locality (Carlini et al. 2008c, 2018; Zurita et al. 2011; Vucetich et al. 2010; Castro et al. 2014) (Table 1), and their paleoecological and habitat preferences (e.g., Defler 2019) could suggest forested-grass land areas. This could be supported also by the presence of abundant remains of *Falcontoxodon* (Carrillo et al. 2018) (Table 1), a taxon that is closely related to other toxodontids characterized by a broad ecological flexibility and diet associated with grasslands and/or forested-grassland areas (MacFadden 2005).

**Fig. 35:**
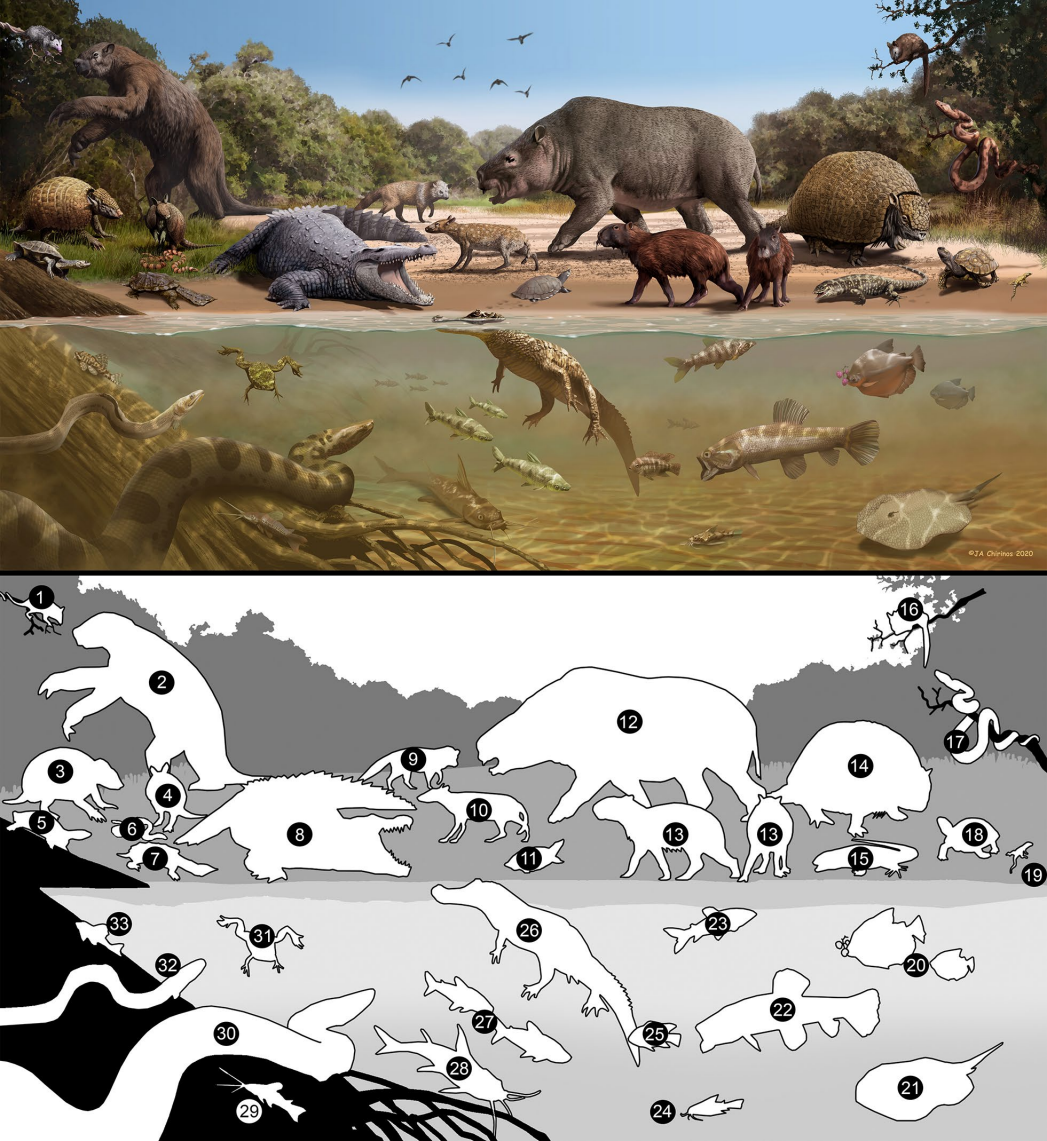
(Top) Life reconstruction of the San Gregorio Formation faunal assemblage, Falcón Basin, Venezuela. Artist: Jaime Chirinos. (Bottom) Key of the reconstruction. (1) cf. *Didelphis* sp. (2) cf. †*Proeremotherium* sp. (3) aff. †*Holmesina floridanus*. (4) †*Pliodasypus vergelianus*. (5) Podocnemididae indet. (6) *Anilius scytale*. (7) *Chelus* sp. (8) †*Crocodylus falconensis*. (9) †*Cyonasua* sp. (10) †Interatheriidae indet. (11) Podocnemididae indet. (12) †*Falcontoxodon* sp. (13) †?*Hydrochoeropsis wayuu*. (14) aff. †*Boreostemma* sp. (15) *Tupinambis* s.l. (16) †*Marisela gregoriana*. (17) *Corallus* sp. (18) *Chelonoidis* sp. (19) Lizard. (20) *Mylossoma* sp. (21) *Potamotrygon* sp. (22) *Hoplias* sp. (23) *Schizodon* cf. *S*. *corti*. (24) cf. *Amblydoras* sp. (25) Cichlidae indet. (26) Caimaninae indet. (27) cf. *Megaleporinus* sp. (28) cf. *Sciades* sp. (29) Callichthyidae indet. (30) *Eunectes* sp. (31) cf. *Pipa* sp. (32) *Synbranchus* sp. (33) Suckermouth catfish (Hypostominae)

The data published by Hambalek et al. (1994) are restricted to the marine Cocuiza Member, with no detailed studies on the Vergel Member palynoflora. Jaramillo et al. (2010) suggested that a xerophyte vegetation dominated the landscape during the accumulation of the Codore Formation in the Urumaco region. However, the palynoflora found in the middle Vergel Member indicates the presence of rainforest elements (Additional file 3), while the palynoflora in the Cocuiza Member, albeit scarce, lacks the rainforest taxa seen in the Vergel member, suggesting a major floristic change toward drier biomes in the Pliocene–Pleistocene transition that also seems to occur in many regions across the Neotropics (Jaramillo 2019). A much wetter precipitation regime for the Urumaco/Ware region had been observed since the early Miocene until at least the middle Pliocene (Scholz et al. 2020; Jaramillo et al. 2020; Pérez-Consuegra et al. 2018) and it is possible that the reduction in precipitation to modern levels occurred at the Pliocene–Pleistocene transition. The small seed and fruit sizes observed in the NCC locality (Fig. 33), in contrast to the pollen, suggest open vegetation environments. In living ecosystems, seeds of small size are most common among herbaceous plants and in grassland environments (Moles et al. 2007). Even though taphonomical processes and the specific depositional environments of the Vergel Member may have biased the selective preservation of small-sized seeds, the natural affinities of this assemblage are also indicative of a low-standing vegetation component in open or partly open environments. These paleobotanical interpretations, the abundance and diversity of freshwater vertebrates, and the ecological preferences of forest-dweller vertebrates described above suggest an environment with mixed forested-grassland areas during the deposition of the Vergel Member (Fig. 35).

*Cocuiza Member*: In contrast to the Vergel Member a continental paleoenvironment, the Cocuiza Member (the middle portion of the San Gregorio Formation) represents a marine environment associated with marine incursion (Hambalek et al. 1994). The presence of marine microfossils (Hambalek et al. 1994; Smith et al. 2010; Additional file 4), ostreid colonies and several other marine molluscan taxa (Rey 1990), echinoids and crustaceans (Aguilera et al. 2010; Mihaljević et al. 2010), and some sharks and bony fishes (Carrillo-Briceño et al. 2018b, table S6; Aguilera et al. 2020) suggests a low to moderate energy coastline environment with an influx of terrigenous sediments (see Rey 1990; Ministerio de Energía y Minas 1997).

The SGOP section (Fig. 3C) is characterized by an interbedding of marine layers (with abundant marine mollusk, crustaceans, rays, and sirenian remains) and lenticular conglomerates (Fig. 2G, H) bearing disarticulated terrestrial vertebrates, suggesting intermittent flows of terrigenous sediments into a littoral environment. For example, the presence of freshwater/terrestrial fauna (including abundant coprolites) in marine sediments (coquinoic limestones) of the Urumaco and Caujarao formations has been interpreted as the result of the input of streams and rivers from the backshore to the littoral marine environment (see Dentzien-Dias et al. 2018, table 2; Carrillo-Briceño et al. 2018a). Most of the cranial and postcranial remains from the conglomeratic layers of the SGOP locality were collected in situ already in fragmentary and isolated conditions, likely suggesting significant transport and deposition during high-energy episodes. A presumed crocodylian coprolite (Fig. 29H1, H2) was collected in the fine sandstone underlying the conglomeratic layer (Fig. 3C). Although it is difficult to infer the distance of origin and the continental paleoenvironments based on allochthonous terrestrial fossils recovered from marine sediments, the presence of terrestrial sloths, glyptodontids, pampatheriids, toxodontids, camelids, and procyonids in the SGOP locality (Additional file 7) suggests their association with grasslands and/or forested-grassland areas (MacFadden 2005; Defler 2019, and references there in). In contrast, the presence of *Caiman* aff. *C. yacare*, *Eunectes*, and podocnemidid turtle remains provides evidence for wetland environments and permanent waters.

## Conclusions

A late Pliocene age is here suggested for the Vergel Member based on its stratigraphical position, palynological content, and the presence of the pampatheriid *Plaina*. A Pleistocene age (Calabrian) is suggested for the Cocuiza Member based on its nannoplankton content and the ^86^Sr/^88^Sr dating.

There are fossils of at least 55 aquatic and terrestrial taxa from two different localities of the San Gregorio Formation: 49 taxa from the Vergel Member and 9 taxa from the Cocuiza Member. From the overall paleodiversity, 28 and 18 fossil taxa are reported for the first time in the fossil record of the Urumaco sequence and Venezuela, respectively. Among them are the first fossil records of the freshwater taxa cf. *Megaleporinus*, *Schizodon*, cf. *Amblydoras*, cf. *Scorpiodoras*, and the pipesnake *Anilius scytale*, all from Pliocene strata of the Vergel Member.

The San Gregorio Formation preserves a diverse assemblage of taxa that lived in the Falcón region after the isolation of northern South American and western Amazon basins. Mixed open grassland/forest areas were surrounded by permanent freshwater systems, contrasting with the current dry environments in the Falcón region. The presence of the cis-Andean freshwater catfishes cf. *Amblydoras*, cf. *Scorpiodoras*, podocnemidid and *Chelus* sp. turtles, the *Eunectes* (anaconda) and pipesnake *Anilius scytale* snakes, as well as some caviomorph neoepiblemid rodents supports the hypothesis that geographical contraction to their extant distribution in northern South America occurred rapidly during at least the last 1.5 Ma. This could suggest marked environmental changes in the region during the early Quaternary and a subsequent extinction/extirpation process related to major climatic drying.

The rodent fauna from San Gregorio Formation (NCC locality) is the only Neogene unit from northern South America documenting the coexistence of caviomorphs (Hydrochoerinae, Neoepiblemidae, and Octodontoidea) and cricetids. This fauna shows the last appearance datum of Neoepiblemidae. To date, no dinomyids were found in such strata, a group quite diverse and abundant during the middle-late Miocene, which suggests that they were possibly in decline when the fossiliferous levels of the Vergel Member were accumulated. Cingulate and pilosan (Phyllophaga) xenarthrans from the Vergel Member are more closely related to the earliest South American immigrant taxa in Central and North America around the time when the Panamá Isthmus was fully established. Carrillo et al. (2018) overlapped the mammalian assemblage of the Vergel Formation with the second and third migratory GABI pulse. However, with the late Pliocene age proposed here for the Vergel Member, this assemblage would have to be reinterpreted within GABI 1.

The occurrence of interatheriids in Pliocene sediments of the San Gregorio Formation could suggest that this mammalian group survived early Neogene climate change inhabiting patchy, likely forested areas in tropical South America. This longer persistence time for basal notoungulates might confirm that tropical areas of northern South America could have been "cradles and museums" of biodiversity (e.g., Jablonski et al. 2006). However, confirmation of these paleobiogeographic patterns requires intensive fieldwork in order to (1) collect, more complete and better-preserved specimens, (2) to increase drastically the sampling effort in the highly vegetated tropics, and (3) for isotaphonomic analysis to rule out any sampling biases in the Neotropics. Based on data from these suggested studies, we will be able to determinate whether the tropics were indeed refugia for different clades that inhabited South America.

## Supplementary Information

**Additional file 1.** Northward Chiguaje Hill section (NCH), San Gregorio Formation, Falcón state, Venezuela.

**Additional file 2.** San Gregorio Río Seco section (SGRS), San Gregorio Formation, Falcón state, Venezuela.

**Additional file 3.** Palynological samples from the Vergel Member, San Gregorio Formation.

**Additional file 4.** Micropaleontological samples from the Cocuiza Member, San Gregorio Formation.

**Additional file 5.** 87Sr/86Sr analyses for the Cocuiza Member of the San Gregorio Formation.

**Additional file 6.** Size of the lower first molar (m1) of AMU-CURS-1327, with respect to other fossil and extinct procyonid genera.

**Additional file 7.** Habitat preferences of San Gregorio aquatic freshwater taxa based on preferences of extant relatives. Localities: Norte Casa Chiguaje (NCC) and San Gregorio Oeste del Pueblo (SGOP). *Fr* freshwater, *Br* brackish, *Ma* marine.

## Data Availability

All the fossil specimens described here are available at the paleontological collections of the Alcaldía Bolivariana de Urumaco (AMU-CURS), Falcón State, Venezuela. All data generated or analyzed during this study are included in this published article and its Additional files.
