# Supplementary material for: A Pliocene–Pleistocene continental biota from Venezuela
Source: Swiss J Palaeontol. 2021 Apr 23;140(1):9. doi: 10.1186/s13358-020-00216-6 (PMC8550326; doi:10.1186/s13358-020-00216-6)
Supplement: Supplementary file 1 — Additional file 1. Northward Chiguaje Hill section (NCH), San Gregorio Formation, Falcón state, Venezuela. [file 13358_2020_216_MOESM1_ESM.pdf]

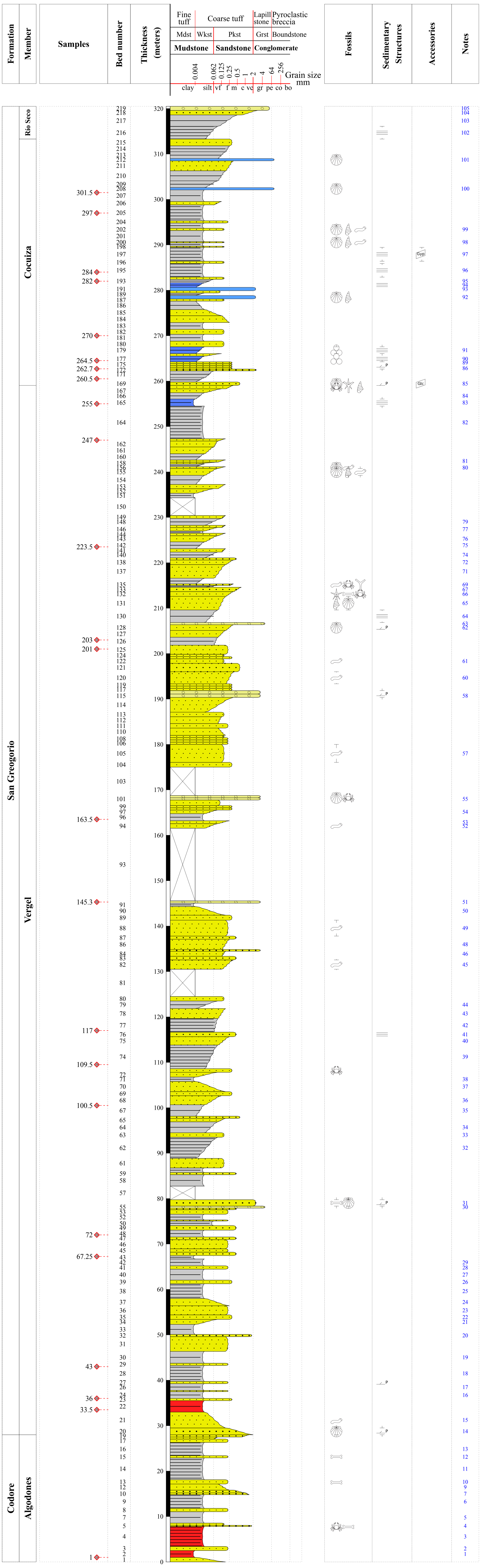

Locality Name

Northward Chiguaje Hill

Scale: 1: 500

Location

Latitude: 11.297480°

Longitude: −70.233207°

Elevation: 35m

Author

German Bayona, Andres Gómez, Carlos Jaramillo, Felipe Lamus, Sara Morón, Luis Quiróz, María C. Ruiz, Rodolfo Sánchez

printed by SDAR, Ortiz J. et al. 2015

LEGEND

Lithology

- mudstone
- sandstone
- conglomerate
- limestone
- Mottled mudstone
- Marl
- covered

Fossils

- crustaceans
- bryozoans
- echinoderms
- bivalves
- gastropods
- vertebrates
- burrows
- foraminifera

Sedimentary structures

- planar lamination
- planar cross bedding

Accessories elements

- glauconite
- gypsum

footnote	base_depth	top_depth	Comments / Notes
1	1	2.5	mottled mudstone interbeded with chanel sandstone
2	2.5	3.4	thick chanel sandstone bed
3	3.4	7.8	mottled mudstone interbeded with chanel sandstone
4	7.8	8.05	sandstone with fragments of fossil mammals, crabs and reptiles
5	8.55	11.05	massive sandy mudstone
6	11.8	14.8	massive sandy mudstone interbeded with thick sandstone beds
7	14.8	15.2	lenticular conglomeratic sandstone bed
8	15.2	15.8	thick sandstone bed with normal grading
9	15.8	17.15	muddy sandstone with normal grading. locally, vertebrates fragments
10	17.15	18.1	sandstone with vertebrates fragments
11	18.1	22.9	massive sandy mudstone interbeded with thick sandstone beds
12	22.9	23.4	thick sandstone bed with vertebrates fragments
13	23.4	26.3	massive sandy mudstone interbeded with thick sandstone beds
14	28	29.5	conglomeratic sandstone with cross−bedding and reworked fossils
15	29.5	33	very thick sandstone with biotrbation
16	36	37.5	brown sandy mudstone
17	37.75	39.25	brown sandy mudstone
18	39.75	43.25	brown sandy mudstone
19	43.75	46.35	massive sandy mudstone
20	49.6	50.1	lenticular conglomeratic sandstone bed
21	52.3	53.4	muddy sandstone with inverse grading
22	53.4	54.4	thin sandstone bed
23	54.4	56.4	thick muddy sandstone bed
24	56.4	58	medium sandstone bed with normal grading and planar cross−bedding
25	58	61.25	massive brown muddy sandstone
26	61.25	62.05	subtabular muddy sandstone
27	62.05	64.45	massive brown muddy sandstone
28	64.45	65.2	subtabular muddy sandstone
29	65.2	66.7	massive brown muddy sandstone
30	77.9	78.35	medium conglomerate bed with erosive base
31	78.35	79.75	thick sandstone with planar cross−bedding, reworked bivalves fragments and vertebrates fragments
32	88.85	93.5	massive brown mudstone with inverse grading
33	93.5	94.5	subtabular muddy sandstone bed
34	94.5	96.9	massive brown mudstone with inverse grading
35	98.15	100.65	massive brown mudstone
36	100.65	102.65	muddy sandstone with inverse grading
37	103.55	105.8	muddy sandstone with normal grading
38	105.8	106.75	massive brown mudstone
39	108.6	113.8	massive brown mudstone with inverse grading
40	113.8	115.65	massive muddy sandstone with inverse grading
41	115.65	116.65	thick sandstone bed with local planar lamination
42	116.65	119.65	massive brown mudstone with inverse grading
43	119.65	121.85	massive muddy sandstone with inverse grading
44	121.85	123.5	massive brown mudstone with inverse grading
45	130.5	132.55	massive muddy sandstone, locally bioturbated, with inverse grading
46	133.35	134.45	massive muddy sandstone with inverse grading
47	134.45	134.85	lenticular conglomeratic sandstone bed
48	134.85	137.1	massive muddy sandstone with inverse grading
49	137.8	141.3	massive muddy sandstone with local bioturbation
50	142.4	144.3	massive muddy sandstone with normal grading
51	145.05	145.55	conglomerate of granules with poor sorting
52	161.55	162.55	muddy sandstone with inverse grading and bioturbation
53	162.55	163.2	sandstone with inverse grading
54	164.75	165.55	muddy sandstone with inverse grading
55	167.75	168.25	thin planar conglomerate bed of sub−rounded granules, matrix supported, with fragments of reworked bivalves and crabs
56	168.25	168.75	thin planar conglomerate bed of sub−rounded granules, matrix supported, with fragments of reworked bivalves and crabs
57	176.05	180	very thick, massive, muddy sandstone bed, locally bioturbated
58	190.35	191.1	conglomerate of granules with planar cross−bedding
59	191.1	191.85	conglomerate of granules with planar cross−bedding
60	193.35	196	massive muddy sandstone with bioturbation, interbeded with thick sandstone beds
61	197.85	198.85	massive muddy sandstone with bioturbation, interbeded with thick sandstone beds
62	205.1	206.35	sandstone with planar cross−bedding and reworked bivalves
63	206.35	206.85	conglomerate of subrounded boulders
64	206.85	209.6	sandy mudstone with incipient planar lamination and inverse grading
65	209.6	212.6	massive muddy sandstone with inverse grading
66	212.6	213.65	subtabular sandstone bed with local calcareous cement and abundant fossils, showing inverse grading
67	213.65	214.6	subtabular sandstone bed with local cacareous cement and abundant fossils, showing inverse grading
68	214.6	215	mudstone bed with inverse grading
69	215	215.35	subtabular sandstone bed with abundant fossils and bioturbation, showing inverse grading
70	215.35	216.6	mudstone bed with inverse grading
71	216.6	219.6	muddy sandstone bed with inverse grading
72	219.6	220.6	subtabular sandstone bed with bivalves and gastropods and inverse grading
73	220.6	221.1	subtabular sandstone bed with fossils bivalves and gastropods and inverse grading
74	221.1	222.35	muddy sandstone with inverse grading
75	223.1	224.6	mudstone with fossil bivalves and inverse grading
76	224.6	225.95	muddy sandstone with bioturbation
77	227	227.75	muddy sandstone with crabs, briozoan and bioturbation; showing inverse grading
78	227.75	228.25	mudstone with inverse grading
79	228.25	229.75	sandstone with bernacles, bivalves and gastropods; showing inverse grading
80	240.75	241.15	sandstone with local calcareous cement and abundant fossils
81	242.15	242.65	sandstone with local calcareous cement and abundant fossils
82	247.3	254.5	brown sandy mudstone interbeded with laminated marl
83	254.5	256.05	laminated marl
84	256.05	257.45	brown sandy mudstone with inverse grading
85	259.05	259.8	conglomeratic sandstone with planar cross−bedding and abundadnt fossils, changing laterally to mollusk packestone
86	262.65	263.05	sandstone with planar cross−bedding
87	263.05	263.45	sandstone with planar cross−bedding
88	263.45	263.85	sandstone with planar cross−bedding
89	263.85	264.25	sandstone with planar cross−bedding
90	264.25	265.5	laminated marl with foraminifera
91	266.05	267.55	laminated marl with foraminifera
92	278.1	278.85	thick packestone of mollusks, locally with oysters in life position
93	279.9	280.65	thick packestone of mollusks, locally with oysters in life position
94	280.65	281.65	laminated marl
95	281.65	282.4	laminated mudstone
96	282.9	285.9	laminated mudstone
97	286.4	289.45	laminated mudstone, locally with gypsum in thin layers and fractures
98	290.45	290.7	medium sandstone, locally with mollusks and bioturbation
99	293.15	293.65	medium sandstone, locally with mollusks and bioturbation
100	302.1	302.6	very thick subtabular oyster bioherm up to 30cm long
101	308.5	309	very thick lenticular oyster bioherm up to 30cm long
102	313.3	316.1	lenticular brown mudstone with planar lamination and inverse grading
103	316.1	318.6	sandy mudstone with inverse grading
104	318.6	319.6	sandstone with inverse grading
105	319.6	320.5	lenticular conglomerate bed with erosive base
