# Supplementary material for: A Pliocene–Pleistocene continental biota from Venezuela
Source: Swiss J Palaeontol. 2021 Apr 23;140(1):9. doi: 10.1186/s13358-020-00216-6 (PMC8550326; doi:10.1186/s13358-020-00216-6)
Supplement: Supplementary file 2 — Additional file 2. San Gregorio Río Seco section (SGRS), San Gregorio Formation, Falcón state, Venezuela. [file 13358_2020_216_MOESM2_ESM.pdf]

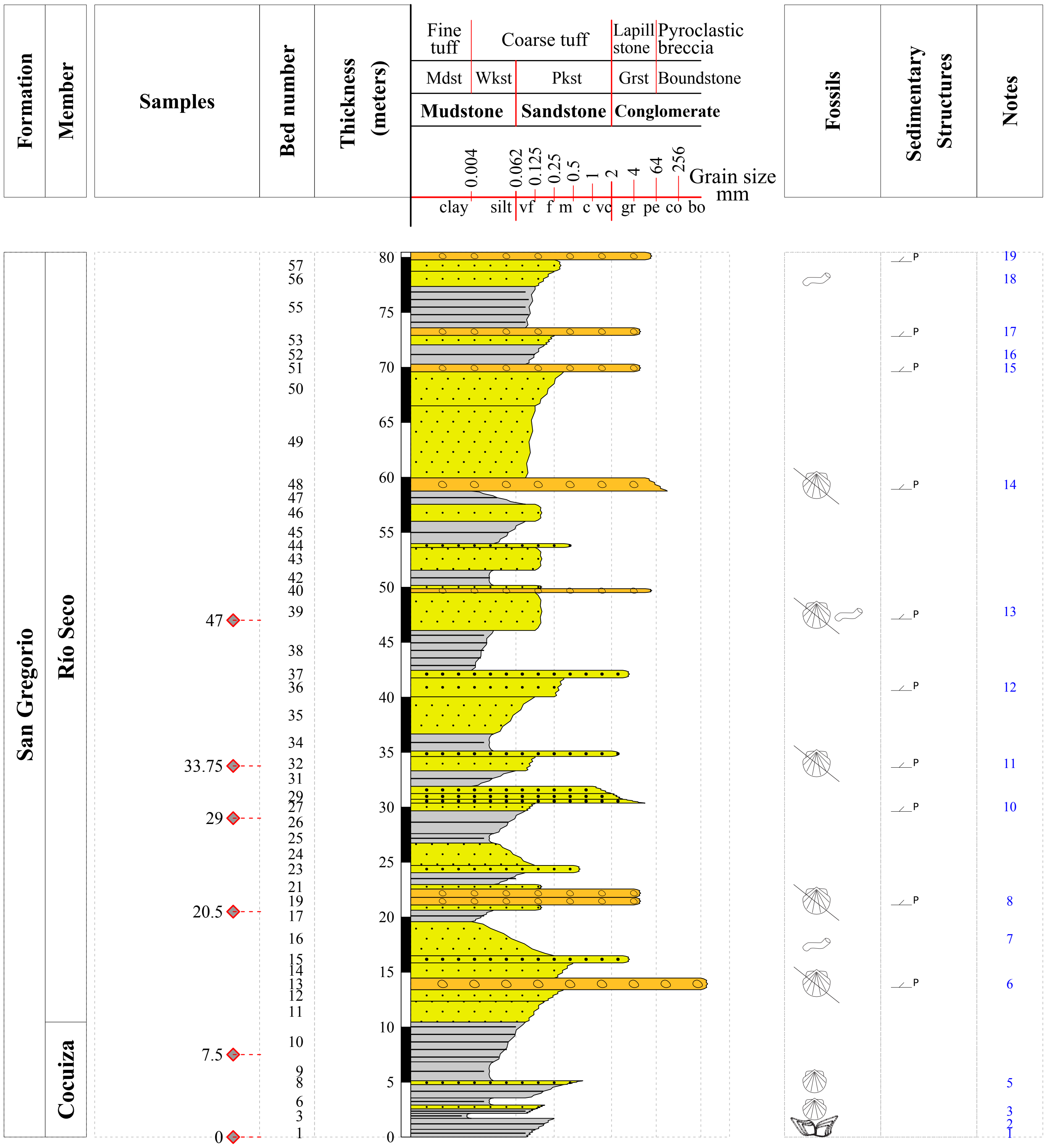

Locality Name

San Gregorio Río Seco

Scale: 1: 500

Location

Latitude: 11.308250°

Longitude: −70.185417°

Elevation: 45m

Author

German Bayona, Andrés Gómez, Carlos Jaramillo, Felipe Lamus, Sara Morón, Luis Quiróz, María C. Ruiz, Rodolfo Sánchez

printed by SDAR, Ortiz J. et al. 2015

LEGEND

Lithology

- mudstone
- sandstone
- conglomerate

Fossils

- bivalves
- burrows
- reworked molluscs
- oyster bioherm

Sedimentary structures

- P planar cross bedding

footnote	base_depth	top_depth	**Comments / Notes**
1	0	0.7	marl, oyster bioherm
2	0.7	1.7	sandy mudstone with limestone at the top
3	2.15	2.56	sandy mudstone with mollusks
4	2.56	2.91	coarsening upward succession with very fine sand at the base and fine sand at the top
5	4.77	5.12	muddy sandstone with fossil mollusks
6	13.4	14.46	conglomerate of subrounded clasts with planar cross−bedding, reworked fossils and erosive base
7	16.49	19.59	muddy sandstone with normal grading and bioturbation
8	21.11	21.8	lenticular conglomerate of subrounded boulders, planar cross−bedding, reworked fossils and erosive surface
9	21.8	22.56	lenticular conglomerate of subrounded boulders, planar crossed stratification, reworked fossils, erossive surface
10	29.67	30.37	muddy sandstone with inverse grading, showing planar cross−bedding
11	33.31	34.61	muddy sandstone with inverse grading, planar cross−bedding, reworked fossils and erosive surface
12	40.04	41.76	sandstone showing inverse grading from fine to fine/medium grain size, with planar cross−bedding and erosive surface
13	46.07	49.52	muddy sandstone with bioturbation, planar cross−bedding, reworked fossils and erosive base
14	58.75	59.95	conglomerate of subrounded clasts, with planar cross−bedding, reworked fossils and erosive surface
15	69.6	70.29	conglomerate of subrounded clasts with planar cross−bedding and erosive surface
16	70.29	72.05	sandy mudstone with inverse grading
17	72.91	73.6	conglomerate with subrounded granules, showing planar cross−bedding and erosive base
18	77.36	78.74	muddy sandstone with inverse grading, showing bioturbation
19	79.78	80.47	conglomerate of subrounded clasts with planar cross−bedding and erosive surface
