# Supplementary figures and images for: A Pliocene–Pleistocene continental biota from Venezuela
Source: Swiss J Palaeontol. 2021 Apr 23;140(1):9. doi: 10.1186/s13358-020-00216-6 (PMC8550326; doi:10.1186/s13358-020-00216-6)

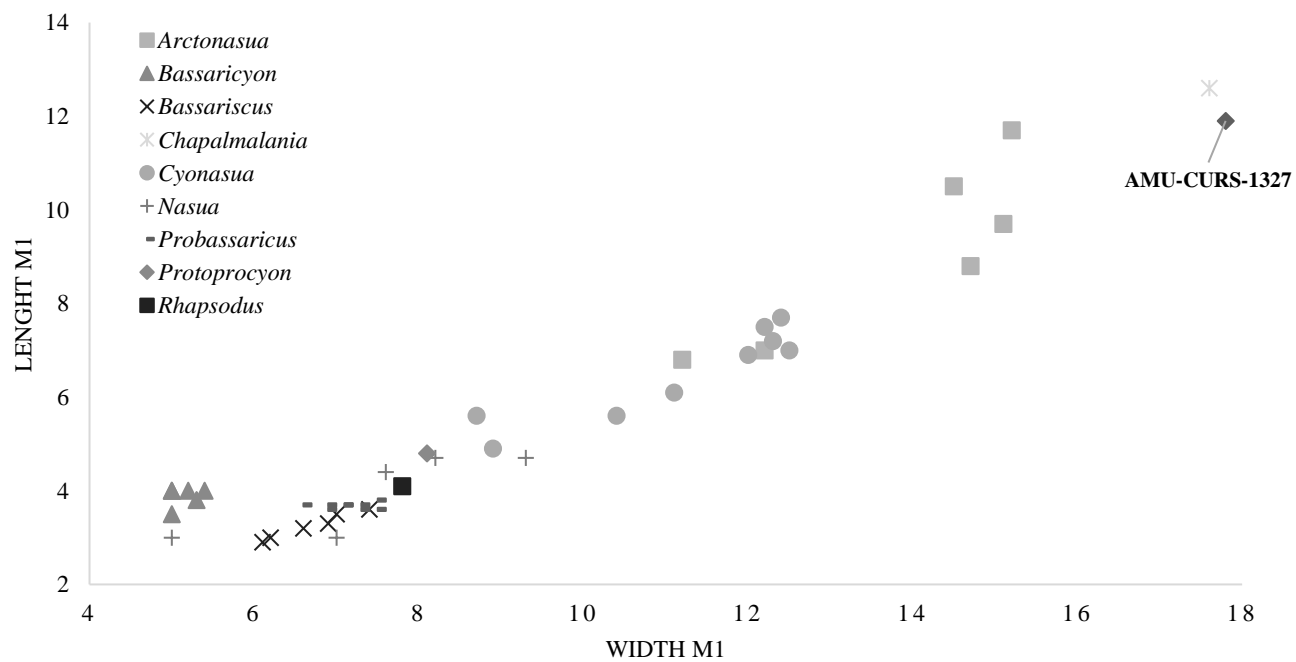

Supplement: Supplementary file 6 — Additional file 6. Size of the lower first molar (m1) of AMU-CURS-1327, with respect to other fossil and extinct procyonid genera. [file 13358_2020_216_MOESM6_ESM.pdf]
